# Following Flavin’s Vibrational Modes to Probe Anharmonicities and Low-Lying Conical Intersections

**DOI:** 10.1021/acs.jpcb.5c05589

**Published:** 2025-12-06

**Authors:** Vy Vu, Samer Gozem

**Affiliations:** Department of Chemistry, 1373 Georgia State University, Atlanta, Georgia 30303, United States

## Abstract

Flavins (e.g., riboflavin, FMN, and FAD) are biologically ubiquitous molecules widely studied using spectroscopic techniques such as ultraviolet–visible (UV/vis), Fourier-transform infrared (FTIR), and Raman spectroscopies. Those spectroscopic methods typically involve the excitation of flavin’s vibrational modes either on the ground or excited state potential energy surfaces. In the case of UV/vis spectroscopy, this vibrational excitation is coupled to an electronic excitation and leads to a Franck–Condon progression that gives flavin its UV/vis absorption profile. To support experimental spectroscopy measurements, it is desirable to use computations that can accurately predict the vibrational frequencies for flavin’s ground and excited electronic states. However, commonly used computational approaches often rely on a harmonic approximation, which may lead to errors for modes that are not harmonic. By using time-dependent density functional theory (TD-DFT) on lumiflavin, a simplified model system to represent flavins, we mapped the ground- and excited-state potential energy surfaces computed along 3N-6 vibrational modes near the ground equilibrium structure and computed the corresponding excited-state energies along those modes. We fitted these computed potentials with second-order polynomials and used goodness-of-fit statistics as indicators of the harmonicity of vibrational modes. Among the modes that display anharmonic behavior, several are Franck–Condon active modes, which can help explain why flavin’s vertical excitation energies reported in the literature do not typically match well with the experimental wavelength of maximum absorption (λ_max_). These calculations help inform future computational and experimental UV/vis, FTIR, and resonance Raman studies of flavins. Some of those potential scans also revealed low-lying conical intersections between the first and second singlet excited states. These intersections constitute potential photophysical deactivation channels that can compete with fluorescence, intersystem crossing, or photoredox chemistry when flavin is in a nonpolar environment.

## Graphical abstract

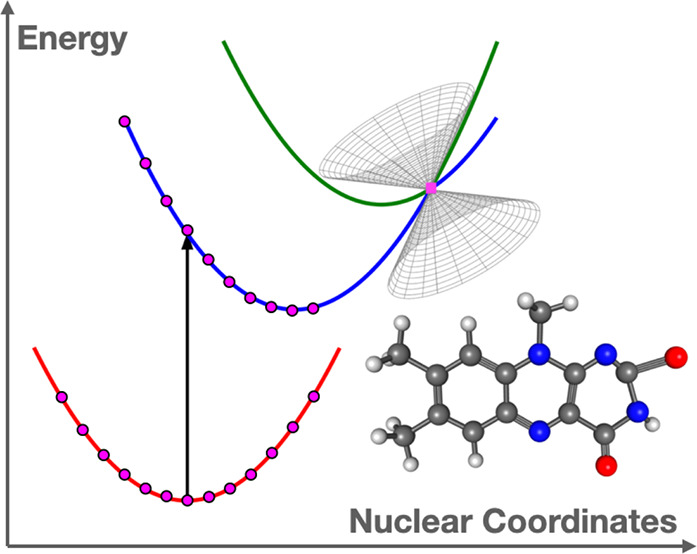

## 1 Introduction

### 1.1 The Flavin Family

Flavins are ubiquitous in nature. Riboflavin (RF, also known as vitamin B_2_) is an essential water-soluble vitamin and has two major coenzyme derivatives: flavin mononucleotide (FMN) and flavin adenine dinucleotide (FAD).^1–3^ These redox-active coenzymes are in bacteria, animals, and plants.^4,5^ RF, FMN, and FAD all share a common dimethylisoalloxazine core structure, which is also common to a smaller model system, lumiflavin (LF, Figure 1).^6^

**Figure 1:**
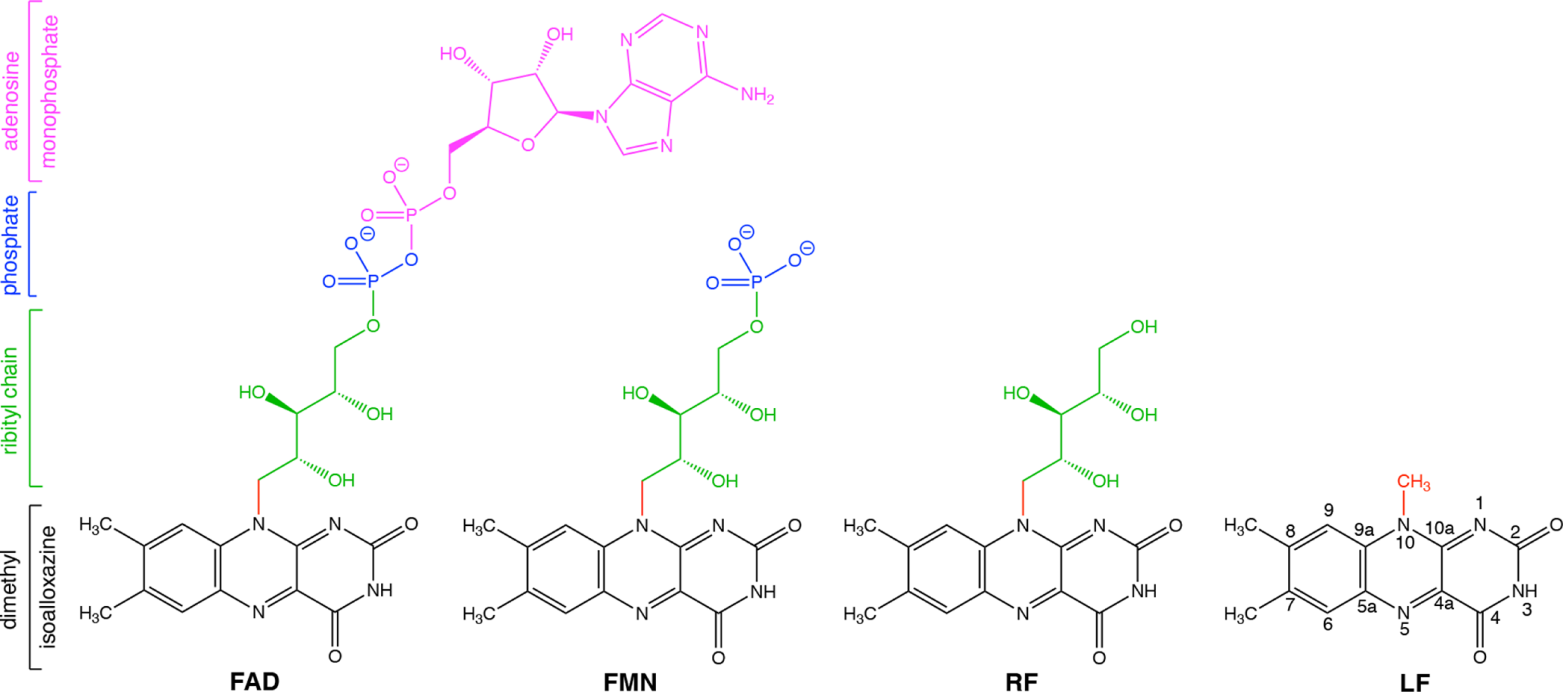
Structure of flavin adenine dinucleotide (FAD), flavin mononucleotide (FMN), riboflavin (RF), and lumiflavin (LF).

Due to their π-electron conjugated isoalloxazine ring, flavins in their oxidized form are chromophores that absorb blue light and appear yellow.^7,8^ This feature of flavins is harnessed by several classes of flavin-binding photoreceptors, such as LOV, BLUF, and cryptochromes, to convert flavin’s photoexcitation energy into a biologically useful signal.^7,9–13^ Flavin’s characteristic ultraviolet–visible (UV/vis) absorption is also widely used to detect and characterize flavins.^8,14,15^ Time-resolved spectroscopies, either in the ultrafast regime or at the millisecond time scale accessible to stopped-flow experiments, are often used to study the dynamics and properties of flavoproteins.^16–22^

Flavin’s absorption spectrum is relatively insensitive to the surrounding protein environment. In contrast to, for instance, the retinal protonated Schiff base (rPSB, a vitamin A derivative) that can absorb at wavelengths ranging from blue to red depending on the protein they are bound to,^23–25^ flavins in their oxidized form always absorb blue light and appear yellow unless they undergo a chemical, charge transfer, or redox transformation. The reason for this has to do with the different electronic characters of the first singlet (S_1_) excited state of those two chromophores; while rPSB undergoes a significant charge transfer upon excitation,^26–29^ flavin instead undergoes a relatively modest rearrangement of atomic charges when excited to its S_1_ state, with charges staying largely localized to the isoalloxazine.^8,30,31^ Still, in flavins, subtle changes in the UV/vis spectrum encode potentially valuable information about the surrounding protein environment. A small shift in absorption can be explained by the presence of a substrate or a protein conformational change.^25,32,33^ FTIR and resonance Raman spectra are also sensitive to hydrogen bonding and other noncovalent interactions between flavin and its environment.^34–38^ This is motivation to develop computational models that can accurately simulate flavin’s UV/vis, FTIR, and Raman spectra while properly accounting for the effect of a protein or solvent environment. Several computational studies have modeled such spectra in the gas phase, in solution, and in protein models.^8,20,37–61^ The latter typically employ hybrid quantum mechanical/molecular mechanical (QM/MM) models.^62–65^ In the case of UV/vis simulations, several computational studies have demonstrated that a quantitative agreement between theory and experiments required models that properly account for both vibrational as well as electronic transitions through the calculation of Franck–Condon factors (FCFs).^43–46,57–59^ In other words, a commonly used vertical approximation, which suggests that the vertical excitation energy should give a reasonable estimate of the most intense vibrational-electron transition, appears to not be valid in flavins. However, the studies cited above have not explored why the vertical approximation does not hold for flavin’s first excited state.

It is generally well recognized that the vertical approximation is, exactly as its name suggests, an approximation, and therefore can fail when underlying assumptions are not reasonable.^66–68^ For instance, the vertical approximation may not hold when excited state relaxation involves small changes along several vibrational modes instead of being dominated by a single mode.^69^ Alternatively, the approximation may also fail when there is a large displacement along a single Franck–Condon (FC) active mode, since the vertical excitation reaches a point on the excited state potential energy surface that is far from the excited state equilibrium and therefore not as well described by approximate electronic structure methods.^68,70^ Such regions of the excited state are especially sensitive to anharmonicity effects.

It would be informative to find out which of the scenarios in the above discussion applies to flavin. We start with Section 1.2 that defines some of the main concepts and terms relevant to this work. This discussion is kept mostly qualitative; for a more detailed discussion, we refer the reader to ref.^69^ and other references therein. This is followed by Sections 2, 3, and 4, where we present the results of computing potential energy scans (PESs) for flavin’s ground and first excited states along ground-state vibrational normal modes local to its dimethylisoalloxazine ring. Those PESs are fit using quadratic functions. Since modeling of molecular vibrations is typically carried out within the harmonic approximation, this exercise is used to identify vibrational normal modes relevant to flavin’s spectroscopy and photophysics and to probe their harmonicity. Deviations from harmonicity are discussed.

While there are other methods that account for anharmonic effects,^71–78^ their routine application to ground and excited states can be prohibitively time-consuming. The approach used in this work does not capture anharmonic coupling between different vibrational modes of flavin but instead gives an indication of which normal modes display a deviation from harmonicity, which is already relevant to UV/vis, FTIR, and (resonance) Raman spectroscopy modeling in flavins.

### 1.2 Background

Figure 2 presents a few key definitions and concepts related to the shape and vibrational broadening of the UV–vis absorption spectra. Excitation from the cold (*v* = 0) ground electronic state (S_0_, red) occurs to one of the vibrational states of the first singlet excited state (S_1_, blue) upon absorption of a photon. This electronic excitation is accompanied by excitation of a vibrational mode of the S_1_ state, resulting in a combined electronic-vibrational excitation. Within the FC principle, which assumes that the transition dipole moment is independent of nuclear motion, the probability of exciting a particular vibrational mode of the S_1_ state depends on the overlap integral of the ground and excited electronic state vibrational wave functions, ⟨χ_1_|χ_0_⟩, known as the FCF:

$$\mathrm{FCF}=\langle \chi_{1}|\chi_{0}\rangle =\int \chi_{v^{\prime },1}^{*}\chi_{v,0}d\tau_{N}$$

**Figure 2:**
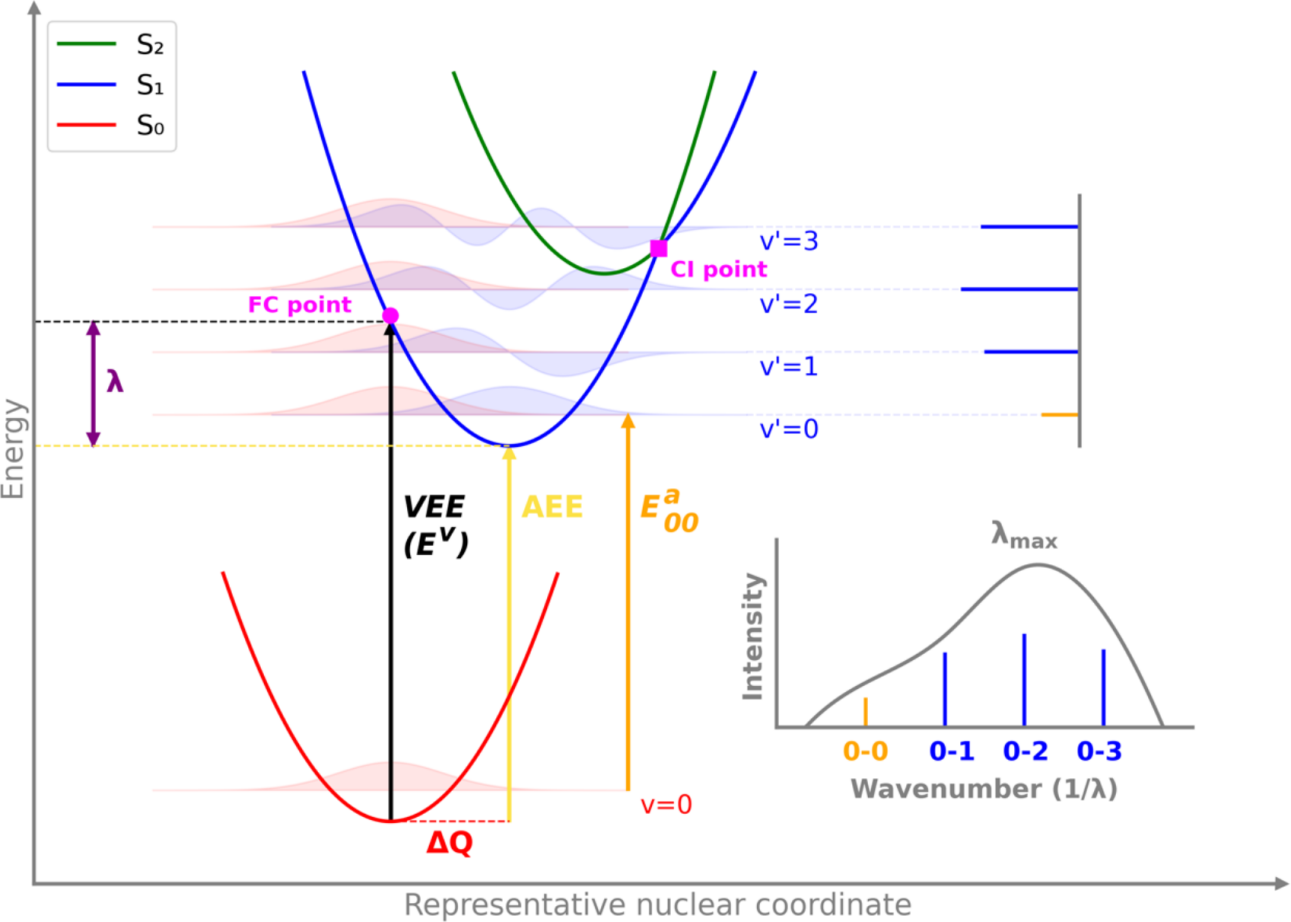
Scheme of ground state (S_0_), first singlet excited state (S_1_), and second singlet excited state (S_2_) potential energy surfaces along a single nuclear coordinate (e.g., bond distance in a diatomic molecule). Vibrational wave functions are shown for the lowest vibrational state of S_0_ and the first four vibrational states of S_1_. The overlaps of the vibrational wave functions of the ground electronic state (red) and the first excited electronic state (blue) determine the intensities of transitions from *v* = 0 of the ground state to *v*′ = 0, 1, 2, 3 of the first singlet excited state. The features labeled are the vertical excitation energy (VEE or *E^v^*), adiabatic excitation energy (AEE), adiabatic 0–0 excitation energy $\left(E_{00}^{a}\right)$ , geometry displacement between the S_1_ and S_0_ minima (Δ*Q*), relaxation energy (λ), Franck–Condon point (FC point), and conical intersection point (CI point). For polyatomic molecules, the AEE, $E_{00}^{a}$ , Δ*Q*, and λ labels are only valid if Δ*Q* involves displacement of a single representative vibrational normal mode that is identical in the ground and excited electronic states. Adapted with permission from ref.^46^ Copyright (2019) Physical Chemistry Chemical Physics and ref.^69^ Copyright (2022) WIREs Computational Molecular Science.

The square of this FCF integral is proportional to the transition intensity. Therefore, the greater this overlap (i.e., the greater the resemblance of the ground and excited state vibrational wave functions), the greater the intensity for a given transition.^79^ Schematically, this can be represented by the overlap between the ground state wave function (red shading) and each of the excited state wave functions (blue shading) in Figure 2; starting from the lowest vibrational state of the electronic ground state (initial wave function), which is the center of the bell-shaped potential, one can compute the overlap with the vibrational wave functions of the electronic excited state (final wave function).^46,79,80^ An accurate description of excitation must account for this coupling between the electronic and vibrational motions, either through sampling of ground state nuclear coordinates or by computing FCFs using vibrational wave functions.

The vertical excitation energy (VEE or *E* ^*v*^) is the energy required to promote an electron from the ground state to an excited state without any change in nuclear geometry (see black arrow in Figure 2). The adiabatic excitation energies (AEE and $E_{00}^{a}$ ) are energy differences between the ground state and excited state minima before and after accounting for zero-point vibrational energies. The latter corresponds to the band origin in the experimental spectra. The change in nuclear coordinates between S_1_ and S_0_ is indicated as Δ*Q* and reflects the extent of geometric reorganization upon excitation.^81^ The position where the vertical excitation energy reaches the excited energy level is known as the FC point.

Figure 2 suggests that the VEE should be close in energy to the vibrational-electronic transition that has the strongest nuclear wave function overlap between the ground and excited state (compare the length of the black arrow to the difference in energy between *v* = 0 and *v*′ = 2 in Figure 2). Therefore, a widely used approximation is that the theoretical VEE should match reasonably well with the experimental λ_max_. However, as discussed in the introduction, there are cases where the vertical approximation fails.

An electronically excited state may intersect with another state having a similar energy. This possibility is also schematically illustrated in Figure 2 where the second excited state (S_2_, green) crosses the S_1_ state at a conical intersection (CI). At such a point, some of the approximations used in the Franck–Condon principle are no longer valid due to the nonadiabatic nature of these crossing points.

It is possible to simulate FCFs using software such as *ez*FCF^69,82^ and FCClasses.^83^ Flavin’s spectra are well-reproduced using TD-B3LYP when computing the AEE and simulating FCFs (e.g., see Figure 3).^33,41,43–47,84^ However, VEEs for the S_1_ state computed using the same TD-B3LYP method do not match well with the experimental λ_max_. The experimental λ_max_ for the S_1_ state is observed near 450 nm, while the VEE $\left(E_{\left(S_{1}\right)}^{v}\right)$ is blue-shifted at 414 nm (dashed line in Figure 3). On the other hand, the vertical excitation approximation appears to work well for the next bright excited state (S_4_ in Figure 3). The S_1_ and S_4_ states both have π,π* character, with two dark states (S_2_ and S_3_) between them.

**Figure 3:**
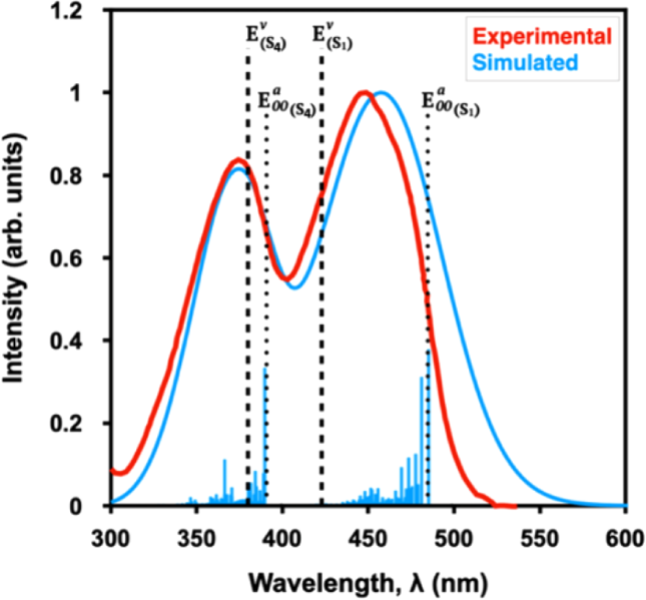
Experimental (red)^85^ and simulated (blue)^46^ UV/vis spectrum for flavin in water. The FCFs were computed for a gas-phase flavin model with B3LYP/cc-pVTZ and using ezFCF^69^ with Duschinsky rotations^86,87^ (blue stick spectra). The adiabatic $\left(E_{00}^{a}\right)$ and vertical (*E* ^*v*^) excitation energies for the first (S_1_) and fourth (S_4_) excited states are indicated by vertical dotted and dashed lines, respectively, using the same level of theory. Adapted with permission from ref.^46^ Copyright (2019) Physical Chemistry Chemical Physics and ref.^85^ Copyright (1974) Biochemistry.

## 2 Methodology

### 2.1 TD-DFT Energy Calculations

This study employs LF (see Figure 1) as a minimal computational model for flavins since it contains the core dimethylisoalloxazine moiety that gives flavin its spectroscopic signature in the visible region,^88^ as well as in the diagnostic FTIR region, where intense C=O, C=C, and C=N bands appear.^89–92^ Compared to FMN and FAD, LF’s smaller size reduces the computational cost of the calculations by reducing the time required for each single-point calculation and lowering the system’s dimensionality; lumiflavin has 87 vibrational modes compared to FMN’s 144 and FAD’s 246.

LF was optimized at the B3LYP/6-31+G* level of theory, followed by a frequency calculation to confirm that the optimized lumiflavin geometry lies on a potential energy minimum along all 87 of its vibrational normal modes. The computed frequencies, IR intensities, and molar extinction coefficients are tabulated in Table S1 in the Supporting Information (SI). The *x*, *y*, and *z* Cartesian vectors describing each normal mode were scaled by a constant factor (*i*) and added to or subtracted from the *x*, *y*, and *z* Cartesian coordinates of the LF equilibrium structure to generate new molecular geometries displaced along each of the 87 vibrational modes. For each vibrational mode, a total of 21 geometries were obtained: the equilibrium structure and 10 structures displaced on each side of the minimum. Initially, the scaling factor *i* was varied using a constant step size from −1 to 1 in increments of 0.1 (i.e., *i* = −1.0, −0.9, −0.8, ... + 1.0). However, that resulted in a distortion that was too large for some normal modes, so we instead used −0.1 ≤ *i* ≤ 0.1 with an increment of 0.01. In total, 1740 (20 × 87) new geometries were created in this way. Those geometries were used for time-dependent density functional theory (TD-DFT) single-point energy calculations using B3LYP/6-31+G*. All DFT and TD-DFT calculations were carried out with Gaussian16.^93^ The choice of functional and basis set is based on earlier testing that has shown that (TD)-B3LYP can describe flavin’s vibrational frequencies, Franck–Condon factors, and excitation energies to the spectroscopic S_1_ state.^37,38,46,84^ Specifically, excitation energies of low-lying excited states computed with TD-B3LYP were benchmarked against multireference and equation-of-motion coupled cluster methods.^84^

### 2.2 Statistical Analysis

For each vibrational mode, the energies of the ground state and the first excited state were tabulated and plotted by using Python 3 scripts. The ground state (S_0_) and first singlet excited state (S_1_) were fitted with a second-degree polynomial function, *y* = *ax* ^2^ + *bx* + *c*. The *x*-axis represents the degree of distortion represented by the scaling factor *i*, and the *y*-values represent the ground and excited state energies. From each of S_0_ and S_1_ along the 87 normal modes, we tabulated the three coefficients obtained by the polynomial fit (*a*, *b*, and *c*). The effects of coefficients *a*, *b*, and *c* in the standard quadratic function *y* = *ax* ^2^ + *bx* + *c* are illustrated in Figure S1, where the parabola is plotted with its vertex at the origin (orange curve). Coefficient *a* determines the parabola’s curvature, while coefficients *b* and *c* govern horizontal and vertical shifts relative to the origin, respectively.

In addition to the *a*, *b*, and *c* coefficients, the coefficient of determination (*R* ^2^) and root-mean-square deviation (RMSD) were calculated relative to the fit. The analysis presented in Section 3 is focused primarily on coefficients *a* and *b*, along with RMSD values, as, in the absence of higher-order polynomial terms used for fitting, these parameters can probe anharmonic characteristics in vibrational modes. Detailed polynomial fits for S_0_, S_1_, and S_2_ curves of lumiflavin, as well as RMSD and *R* ^2^ values, are shown in Figure S5. We also computed the ratio of the RMSD to the average energy (RMSD/*E*_average_) using the equations below:

$$\mathrm{For ground states}:,RMSD/E_{\mathrm{average}}=\mathrm{RMSD}÷\frac{\overset{n}{\underset{i=1}{\sum }}y_{i}}{n}$$

$$\mathrm{For excited states}:,RMSD/E_{\mathrm{average}}=\mathrm{RMSD}÷\left(\frac{\overset{n}{\underset{i=1}{\sum }}y_{i}}{n}-2.9934006\right)$$

where *n* is the total number of data points (21 per normal mode in this study), *y*_*i*_ are the individual energy values, and 2.9934006 is the gas-phase TD-DFT excitation energy in eV. This ratio is used to report the RMSD in relative terms by normalizing them to the average energy. In this way, errors associated with normal modes that induce a large change in the energy do not also appear to have large associated errors. The ΔRMSD/*E*_average_ is then computed as the difference of ΔRMSD/*E*_average_ between S_1_ and S_0_.

The vibration normal modes for each frequency were categorized using the Vibrational Energy Distribution Analysis program (VEDA4xx).^94^ In the vibrational distribution analysis, normal modes are typically represented as linear combinations of internal coordinates corresponding to specific stretching (STRE), bending (BEND), torsional (TORS), or out-of-plane (OUT) local modes. These molecular motions are illustrated in Figure S2. VEDA analyzes the Potential Energy Distribution in percentages (%PED), which quantifies the contributions of various internal coordinates to each normal mode of vibration.

An overview of the protocol followed for the TD-DFT potential energy scans (PESs) and statistical analysis is summarized in Figure 4.

**Figure 4:**
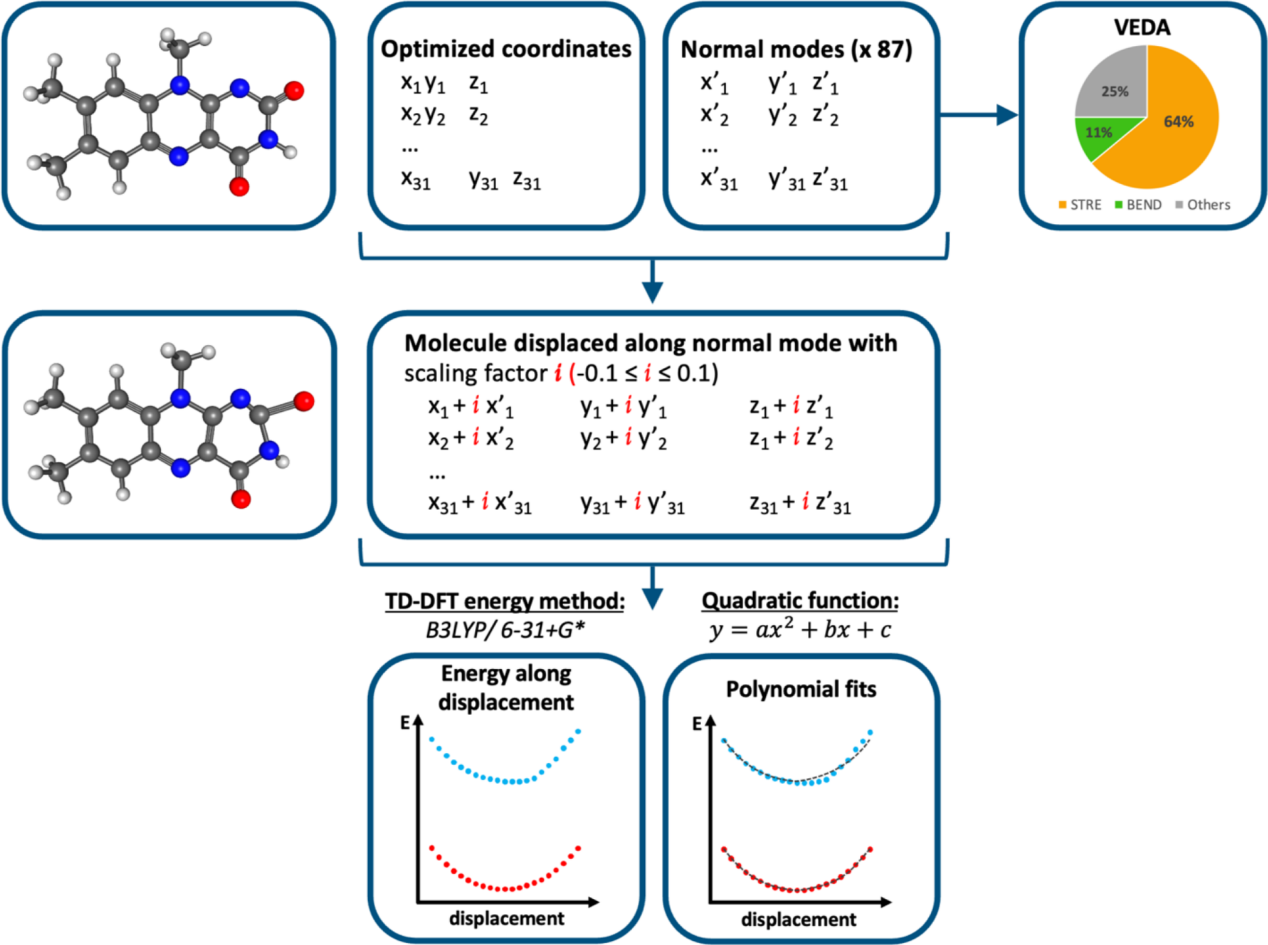
Computational protocol for generating ground and excited state potential energy scans (PESs) near the Franck–Condon point along 87 vibrational modes. For each mode, a polynomial fit is performed, as shown in the last panel. Vibrational Energy Distribution Analysis (VEDA) is carried out to categorize the normal modes.

## 3 Results

Four vibrational modes (modes 31, 34, 74, and 75) initially exhibited a very strong apparent deviation from harmonicity in the S_1_ state (Figure S3 in the SI). Further analysis revealed that this was caused by crossing with the S_2_ state along the PES. Figure 5 displays a schematic representation of such a crossing and reports the energy differences between the encountered CI points and the minimum of the S_1_ PES along each mode (Δ*E*_CI/S1min_). All four modes involve either improper torsion or stretching of the carbonyl groups located on flavin’s pyrimidine ring.

**Figure 5:**
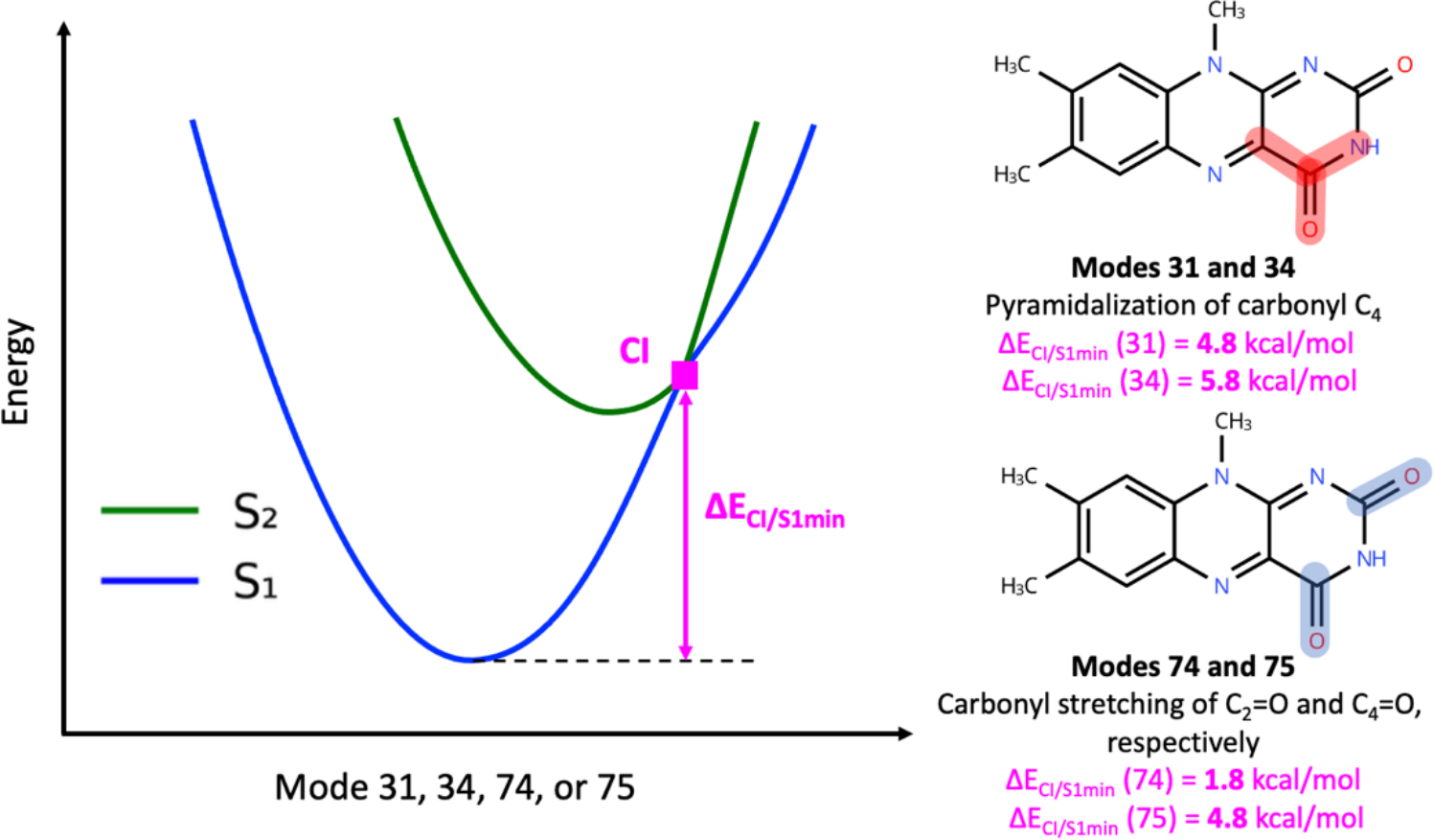
Left: Schematic representation of CI crossings encountered along 4 normal mode PESs. Right: A description of the main internal coordinates involved in those normal modes (determined using VEDA) and the relative energy of the CI point encountered along those modes relative to the S_1_ minimum (Δ*E*_CI/S1min_).

For those four modes, we repeated the quadratic fitting by following the state with the same electronic character past the CI point (i.e., following the diabatic state that gives a continuous energy surface with a consistent electronic character instead of simply following the adiabatic state with the lowest energy). In Figure 5, this would mean using the S_2_ state (green line) to the right of the CI instead of continuing to use the S_1_ state (blue line) for the fitting. The RMSD using those new fits was significantly reduced compared to the fits that only considered the energetic ordering of the states (Figure S4). Detailed polynomial fits for S_0_, S_1_, and S_2_ curves, as well as RMSD and *R* ^2^ values after fitting S_1_ and S_2_ at the CI point for modes 31, 34, 74, and 75, are shown in Figure S4.

Figure 6a presents RMSD calculations indicating the goodness of fit of a quadratic polynomial function for each of the S_1_ and S_0_ potential energy surfaces along lumiflavin’s 87 vibrational modes. Generally, the trends for the S_0_ states and S_1_ states are similar; if a particular ground state is anharmonic (i.e., not well fit using a second-order polynomial), the same is usually true for the excited state (S_1_) scan along the same mode. Examples of modes that are almost equally anharmonic in both the ground and excited states are 37, 39, 84, and 87. However, it is also informative to look at *differences* in RMSD (ΔRMSD) between the ground and first excited state. These differences are plotted in Figure 6b to highlight modes where the excited state shows larger deviations from harmonicity in comparison to the ground state. Additionally, in Figure 6b we use colors to include an analysis of the vibrational motions for each frequency using the %PED categories from VEDA. The vibrational motions were categorized into six types: torsional (TORS), bending (BEND), out-of-plane (OUT), stretching (STRE) with heavy atoms (such as nitrogen and oxygen atoms), stretching (STRE) with hydrogen atoms, and MIX for motions involving more than one type of %PED. The resulting graphical representations highlight the vibrational modes that are affected by excitation to the S_1_ state: mode 31 (mixing between out-of-plane and torsion), 33, 34 (out-of-plane), 70, 73 (mixing between bending and stretching with heavy atoms), and 74 (stretching with heavy atoms).

**Figure 6:**
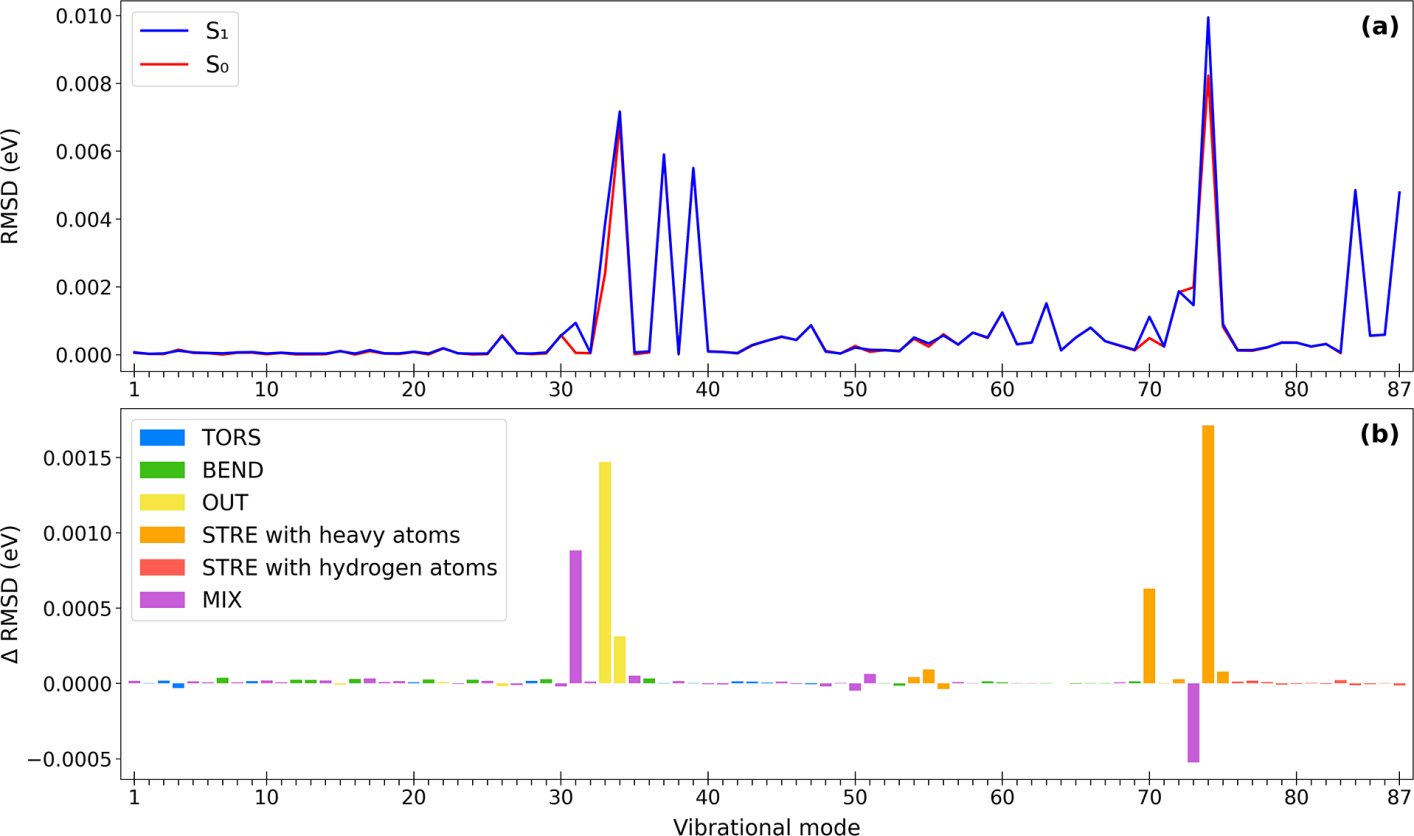
(a) RMSD values of S_1_ and S_0_ and (b) ΔRMSD values categorized by their vibrational modes along the PES of each of the 87 frequencies of lumiflavin.

In Figure 7a,b, we provide a further breakdown of the differences in the ground and excited states’ parabolic functions in terms of the coefficients *a* and *b*. The differences in *a* between the first excited state and the ground state are all negative, indicating that all modes have smaller curvature in the excited state compared to the ground state. Coefficient *b*, governing horizontal vertex shifts along normal modes, can either indicate Franck–Condon activity (displacement of the excited state relative to the ground state equilibrium) or be an indicator of anharmonicity.

**Figure 7:**
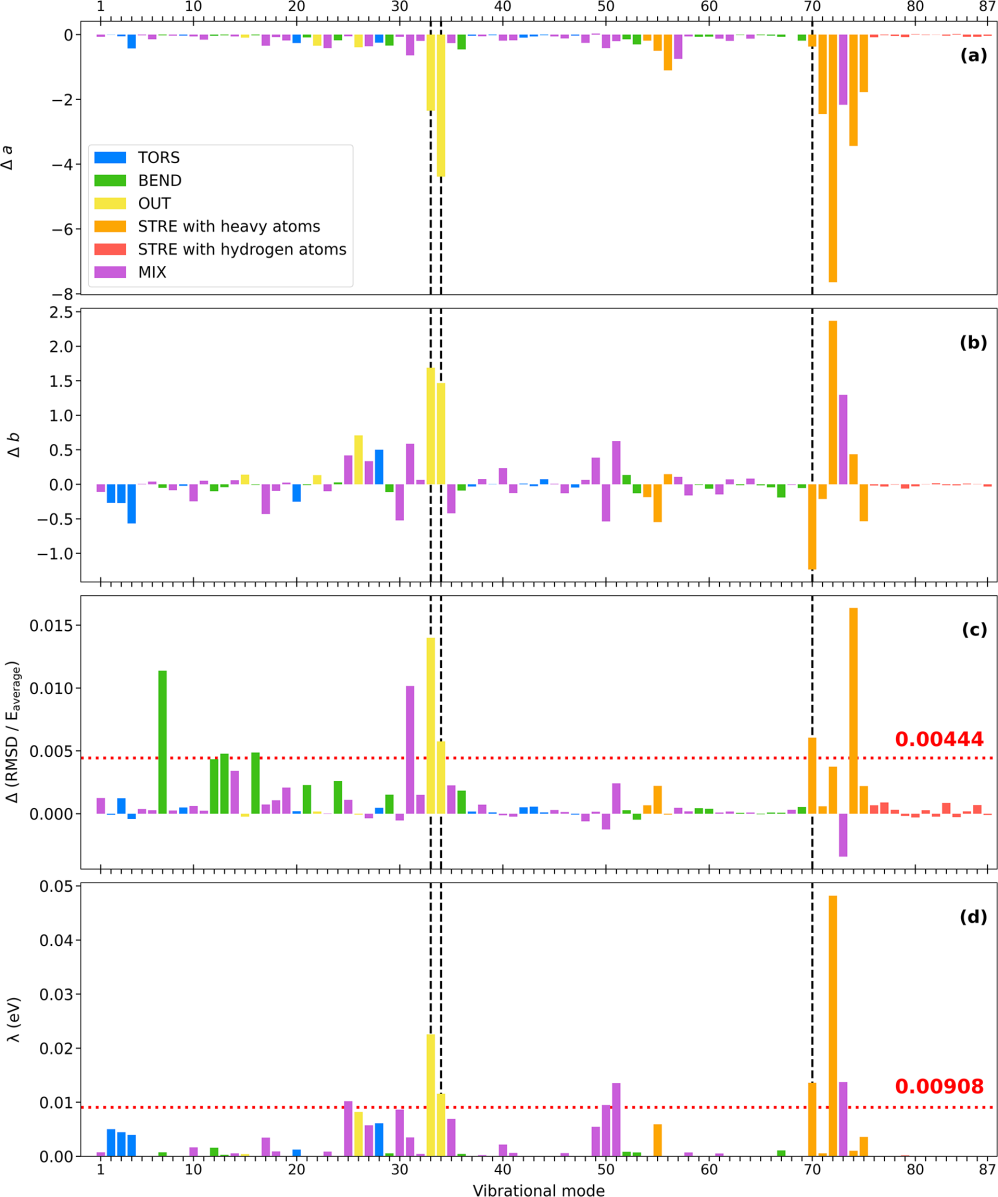
Comparison between (a) Δ*a*, (b) Δ*b*, (c) ΔRMSD/*E*_average_, and (d) relaxation energy (λ) along each of the 87 ground state normal modes of lumiflavin. The matching modes are represented in black dashed lines.

By default, each vibrational mode is normalized such that the sum of the squares of the normal mode vector in three dimensions is equal to 1. However, due to this vector normalization, not all normal modes affect energy to the same extent. For example, distortion along normal modes that are highly localized to a single bond vibration will result in much higher energies compared with a more delocalized mode. In the latter case, the vector represents a sum of smaller distortions that do not significantly perturb the system’s energy because no one mode goes too far from the equilibrium geometry. Because of this, localized and delocalized modes are not compared on an equal footing; it can be expected that localized normal modes deviate (energetically) the most from equilibrium and will also appear to display the highest deviation from harmonicity. Therefore, in Figure 7, we focus on reporting relative harmonicity metrics of the ground and excited states (Δ*a*, Δ*b*, etc.) rather than simply reporting on the “absolute” harmonicity metrics of the ground and excited states. The ΔRMSD is also not reported in absolute terms but is reported relative to the energy change caused by the distortion; in Figure 7c, we examine the differences between the ground and first excited state in terms of the ratio ΔRMSD/*E*_average_ using the formulas provided in Section 2. The important modes that stand out in Figure 7c are modes 7, 13, 16, 31, 33, 34, 70, and 74 (i.e., these modes have a value more than one standard deviation above the average, 0.00444; see Table S2). All those modes are more anharmonic in the excited state than in the ground state. Notably, these modes also generally have a large change in coefficient *b*.

A similar analysis is carried out, focused only on the ground state (S_0_) ΔRMSD/*E*_average_ in the SI. We found that modes 34, 37, 39, 60, 63, 74, 84, 85, 86, and 87 exhibit anharmonic behavior in the ground state (categorized based on having a value more than one standard deviation above the average, i.e., above 0.029659; see Table S3).

To determine modes that are FC active, Figure 7d reports the excited-state energy relaxation along the ground-state normal mode (λ, not to be confused with the earlier use of Lambda in the manuscript, where instead it represented wavelength). In other words, λ represents the difference in energy between the FC point and the lowest energy point on the excited state along each specific vibrational normal mode projected on the excited state. Duschinsky rotations, which can potentially be used to account for changes in the normal modes between two different electronic states, are not used here.^86,87^

Modes that are FC active will have a larger λ. In Figure 7d, we find that modes 25, 33, 34, 50, 51, 70, 72, and 73 stand out as having λ values that are larger than one standard deviation above the average λ (i.e., above 0.00908 eV; see Table S4). Therefore, the total relaxation energy (represented as the collective λ in Figure 2 which represents the overall difference between the electronic AEE and VEE) is distributed over a few key normal modes.

The results of additional tests using the larger scaling factors, results before accounting for state flipping at the CI, and other statistical analyses are presented in ref.^95^

## 4 Discussion

Along 87 PESs following LF’s vibrational normal modes, 4 led to a crossing between the first (S_1_) and second (S_2_) excited singlet states. All four of those modes involve carbonyl stretching or pyramidalization modes. This is consistent with TD-DFT calculations indicating that LF’s S_1_ state has π,π* character and is a bright state, while the S_2_ state is a dark state that has n_0_,π* character involving excitation of the lone pair of the carbonyl oxygen.^55^ Here, we find that this n_0_,π* can be easily accessed *in vacuo* from the S_1_ π,π* state, with the CI encountered along mode 74 being as low as 1.8 kcal/mol (∼630 cm^–1^) relative to the S_1_ minimum. Benchmark studies on the relative energetics of π,π* and n_N_,π* suggest that this barrier may be underestimated with TD-DFT.^84^ On the other hand, this CI may not even be the minimum energy CI along the S_2_/S_1_ intersection seam. Therefore, the calculations suggest that the S_2_ state can be accessed from the S_1_ state even from the *v*′ = 0 vibrational energy level of the C=O stretching mode. The implications for flavin’s photophysics are that the S_2_ n_0_,π* state can serve as an additional channel for nonradiative decay that competes with other processes like fluorescence, intersystem crossing, or photoredox chemistry. However, this low-energy access to the S_2_ state is only expected to occur *in vacuo* or in nonpolar environments where the C=O is not hydrogen bonded; a hydrogen bonding or polar interaction with flavin’s carbonyl groups, such as in aqueous solvent or protein with polar interactions near the flavin active site, will destabilize the S_2_ n_0_,π* relative to the S_1_ π,π* state^55^ and will therefore raise the relative energy of the S_2_/S_1_ CI.

Figure 7 presented Δ*a*, Δ*b*, ΔRMSD/*E*_average_, and λ all in one figure. The goal was to determine if any of the FC active modes have decreased harmonicity upon excitation to the S_1_ state. Indeed, we found several such modes. The relationship between anharmonicity and FC activity can be categorized into eight cases, into which we place the normal modes. Modes are selected in each case based on the magnitude of their ΔRMSD/*E*_average_ (as a probe of anharmonicity) and on λ (as a probe for equilibrium shift/FC activity):

- Case 1 (mode 34): S_0_ and S_1_ PESs both display anharmonicity along this mode, but S_1_ is more anharmonic than S_0_, and the mode is FC active.
- Case 2 (mode 74): S_0_ and S_1_ PESs both display anharmonicity along this mode, but S_1_ is more anharmonic than S_0_, and the mode is FC inactive.
- Case 3 (no modes): S_0_ and S_1_ PESs both display similar anharmonicity, and the mode is FC active.
- Case 4 (modes 37, 39, 60, 63, 84, 85, 86, and 87): S_0_ and S_1_ PESs both display similar anharmonicity, and the modes are FC inactive.
- Case 5 (modes 33 and 70): S_0_ PES is relatively harmonic, S_1_ PES is relatively anharmonic, and the modes are FC active.
- Case 6 (modes 7, 13, 16, and 31): S_0_ PES is relatively harmonic, S_1_ PES is relatively anharmonic, and the modes are FC inactive.
- Case 7 (modes 25, 50, 51, 72, and 73): S_0_ and S_1_ PESs are both relatively harmonic, and the modes are FC active.
- Case 8 (all other modes not listed above): S_0_ and S_1_ PESs are both relatively harmonic, and the modes are FC inactive.

These cases are summarized in Figure 8. We specifically find that modes 33, 34, and 70 exhibit a larger deviation from harmonicity in their excited S_1_ state than in their ground state while also being FC active. The anharmonicity of the S_1_ state of mode 34 may be associated with its mixing with the S_2_ state (see Figure 5). Figure 9 provides a summary of the VEDA analysis, highlighting internal coordinates that most contribute to those normal modes. Modes 33 and 34 commonly exhibit out-of-plane pyramidalizations in the pyrimidine ring, centered at the C2 and C4 carbonyl atoms. In mode 34, pyramidalization of the C_10a_ atom is also involved. Mode 70 is instead characterized by stretching of the N_5_–C_4a_ and N_1_–C_10a_ bonds. Those two bonds formally have double-bond character in the ground state but gain single-bond character in the S_1_ state due to π,π* character of the excitation.^8,53,96–99^ Therefore, this mode is strongly tied to the electronic nature of the S_0_–S_1_ excitation, and its anharmonicity in the S_1_ state, along with the other two FC-active modes, may help explain why flavin’s VEE does not match well with experimental λ_max_ without the calculation of FC factors.^43–46,57–59^

**Figure 8:**
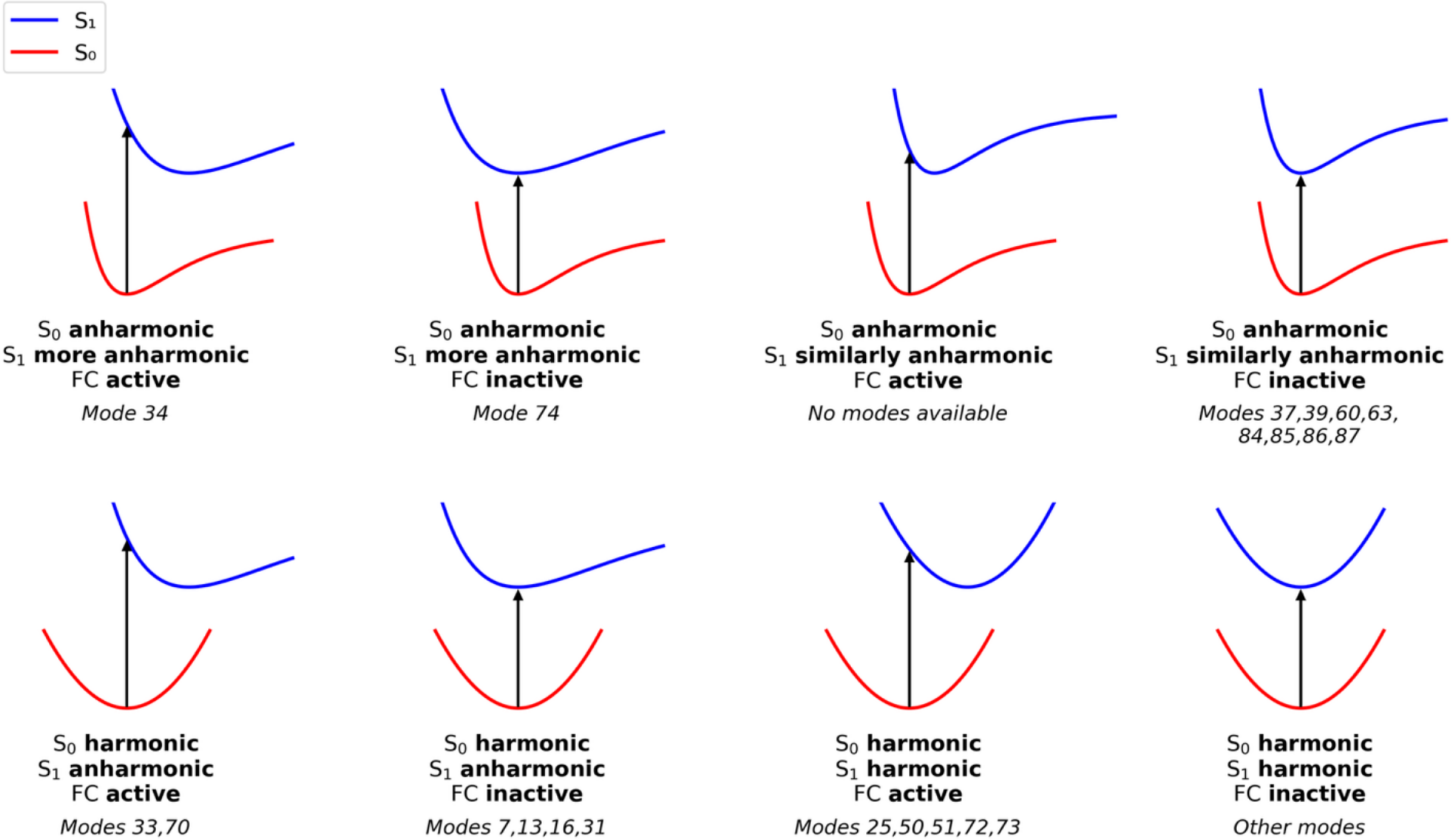
Eight categories showing the relationship between anharmonicity and FC activity with the correlated vibrational modes.

**Figure 9:**
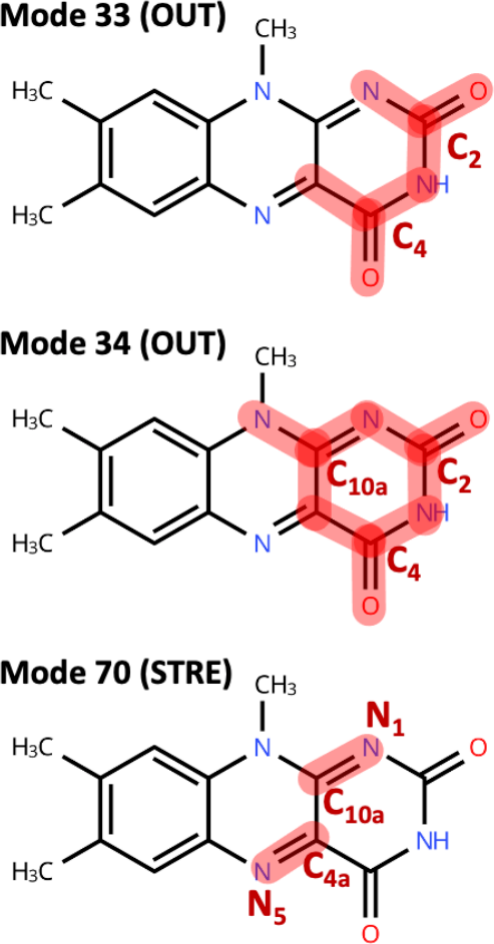
Main contributions to vibrational normal modes 33, 34, and 70 are based on VEDA.

Table S1 indicates that modes 71, 72, 74, and 75 are the four most intense bands in the so-called diagnostic window in the range from ca. 1300 to 1800 cm^–1^. Out of those, only mode 74, corresponding to the lower-frequency C=O carbonyl stretching mode, is listed as having anharmonic character in its ground state. This can explain why molecular dynamics is required along with QM or QM/MM calculations to reproduce its experimental band shape.^37,38,50–52^

## 5 Conclusion

In this work, we mapped the ground and first singlet excited state potential energy of LF along its 87 vibrational normal modes. Those potential energy scans were fit with parabolic functions to determine whether they are well described by a harmonic function or not. Those fits were also used to estimate the relaxation energy (λ) of the excited state along each normal mode to determine which modes are FC active.

Conical intersections with S_2_ were encountered along four of the S_1_ potential energy scans for normal modes associated with carbonyl pyramidalization and stretching, consistent with the n_0_,π* character of the S_2_ state. After fitting the diabatic states for those modes, the parabolic coefficients and RMSD of the harmonic function fit were used to detect the anharmonicity of all 87 normal modes in the ground and excited states. This approach does not capture anharmonic coupling between modes but would capture deviations from harmonicity along a single mode’s PES. We found three modes that are simultaneously FC active and that exhibited decreased harmonicity in the excited state. One of those modes involves the N_5_–C_4a_ and N_1_–C_10a_ bonds, which are strongly coupled to the changes in the electronic character of the S_0_–S_1_ excitation. Their deviation from harmonicity in the excited state helps explain why flavin does not appear to follow the vertical approximation.

The finding that specific vibrational modes relevant to flavin’s Franck–Condon profile and photophysics display anharmonicity has implications for flavin’s IR and Raman spectroscopy as well as its UV/vis absorption and photophysics. Standard IR and Raman simulations, which often rely on harmonic models, are likely to be less accurate for these anharmonic vibrational modes unless a model incorporates molecular dynamics for the calculation of vibrational frequencies and intensities. In the case of IR spectra, anharmonicity was detected in one of the more intense bands in the mid-IR range, corresponding to a C=O stretch.

## Supplementary Material



## Data Availability

The data underlying this study are available in the published article and its Supporting Information. Raw data files are available on GitHub at https://github.com/gozem-gsu/Lumiflavin-vibrational-PESs.

## Notes

Representation of coefficients *a*, *b*, and *c* in the standard quadratic function; definitions of internal coordinates used in VEDA; RMSD values of S_1_ and S_0_ before accounting for S_1_/S_2_ conical intersections; polynomial before and after accounting for S_1_/S_2_ conical intersections; computed potential energy scans and polynomial fits for 87 normal modes; vibrational frequencies and IR intensities for the 87 normal modes; statistical analyses for ΔRMSD/*E*_average_, S_0_ RMSD/*E*_average_, and relaxation energy (λ) (PDF)
