# Supplementary material for: Following Flavin’s Vibrational Modes to Probe Anharmonicities and Low-Lying Conical Intersections
Source: J Phys Chem B. 2025 Dec 6;129(50):12853–64. doi: 10.1021/acs.jpcb.5c05589 (PMC12720234; doi:10.1021/acs.jpcb.5c05589)
Supplement: Supplementary file 1 [file jp5c05589_si_001.pdf]

Supporting Information for:

Following Flavin's Vibrational Modes to Probe Anharmonicities and Low-lying Conical Intersections

Vy Vu, Samer Gozem*

Department of Chemistry, Georgia State University, Atlanta, Georgia

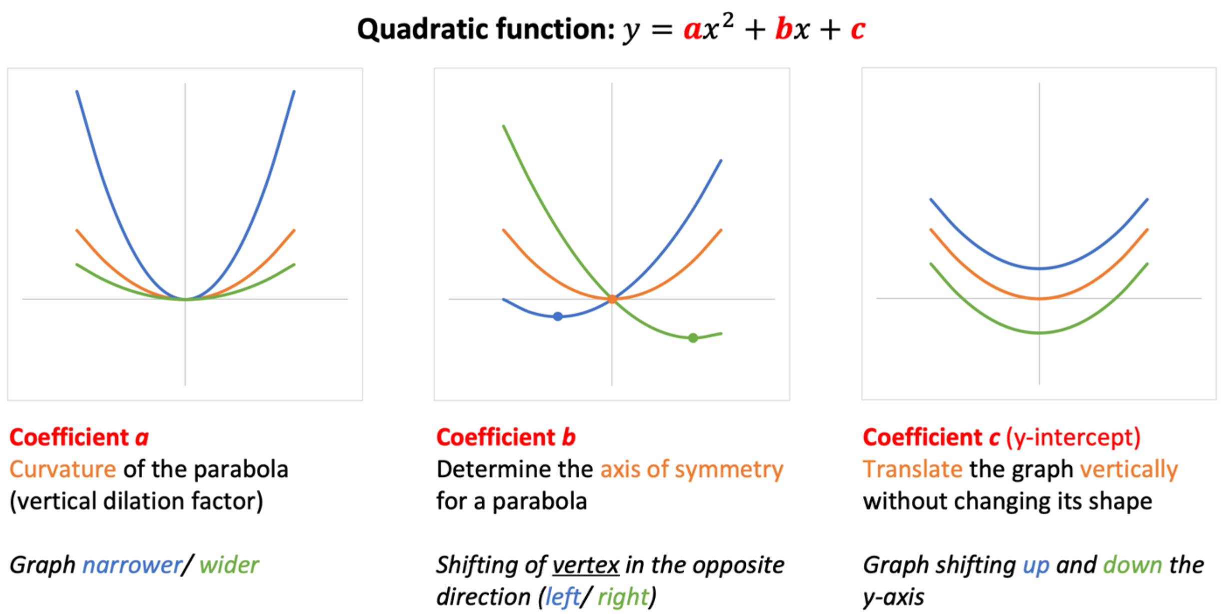

Fig. S1 *Interpretation of coefficient a, b, and c in the standard quadratic function. The standard curve is plotted with its vertex at the origin (orange curve) to show the effect when changing the values of a, b, and c (blue and green curves).*

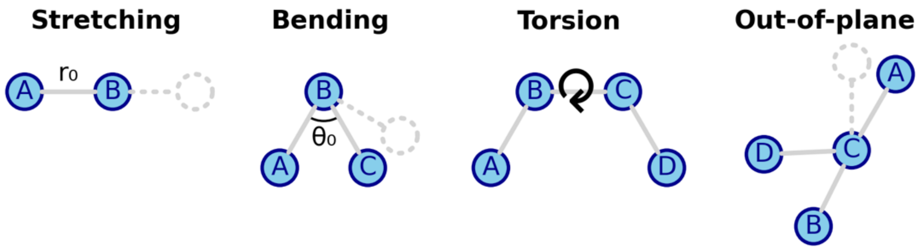

Fig. S2 *Interatomic interactions as categorized by VEDA.*

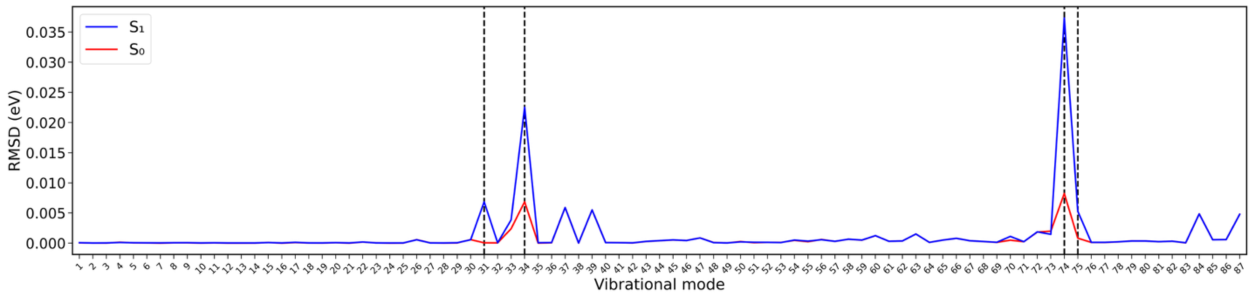

Fig. S3 *RMSD values of S_1 and S_0 and before fitting S_1 and S_2 at CI point along the potential energy scan of each of the 87 frequencies of lumiflavin. Modes having large gap between S_1 and S_0 are represented in black dash lines.*

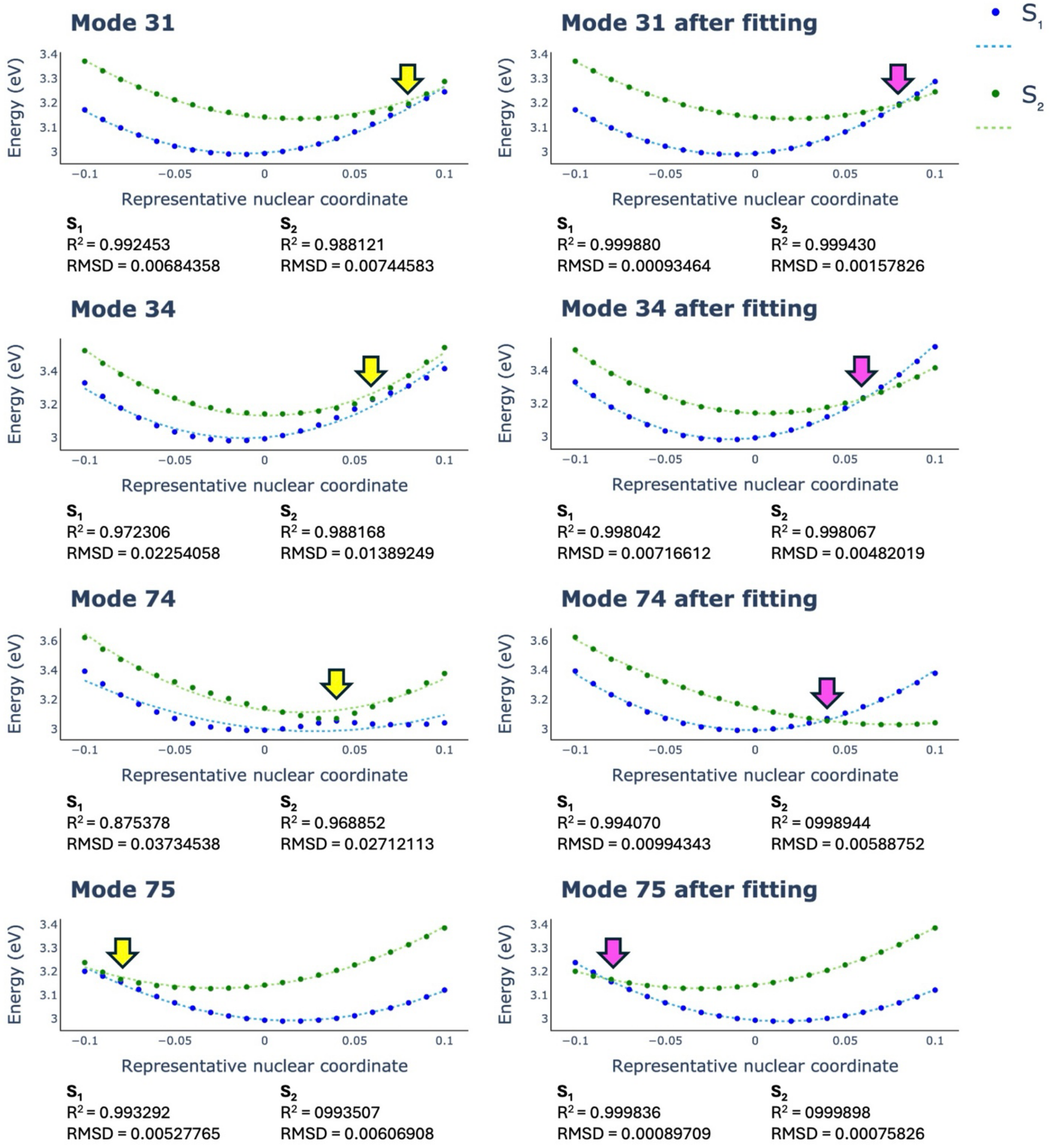

Fig. S4 *Fitting the energy levels of S_1 (blue) and S_2 (green) for vibrational modes 31, 34, 74, and 75 with CI points before the accounting for state flipping (yellow arrows) and after fitting the curves in a way that accounts for state flipping (pink arrows).*

Mode 1

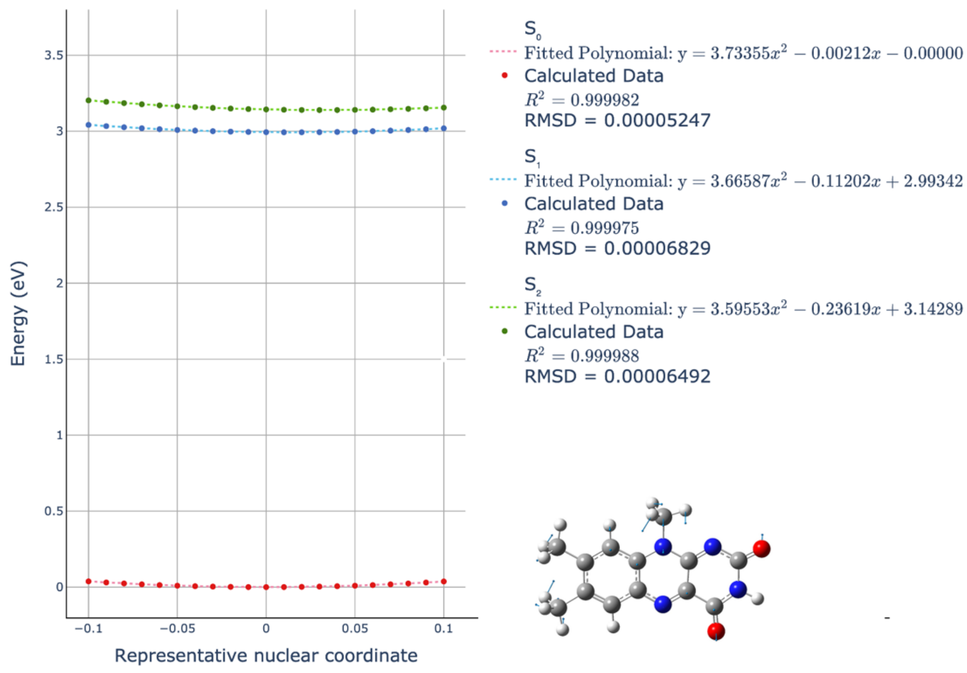

Mode 2

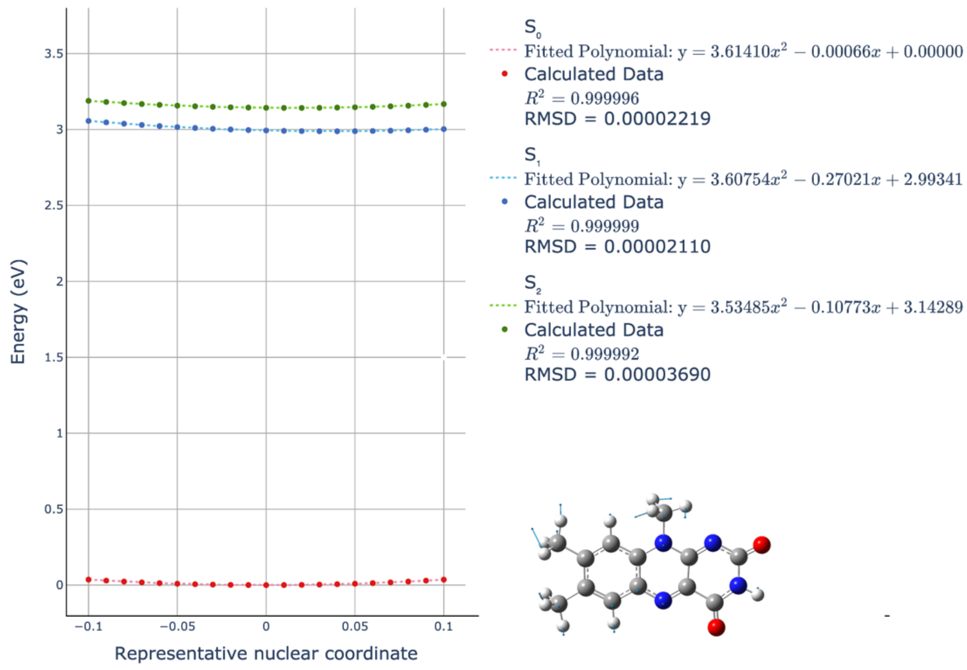

Fig. S5 *Lumiflavin potential energy scans (scaling factor -0.1 ≤ i ≤ 0.1) at ground state (S_0), first excited state (S_1), and second excited states (S_2) with vibrational motion, quadratic equation, R^2, and RMSD values for 87 vibrational modes.* *(continued on next page)*

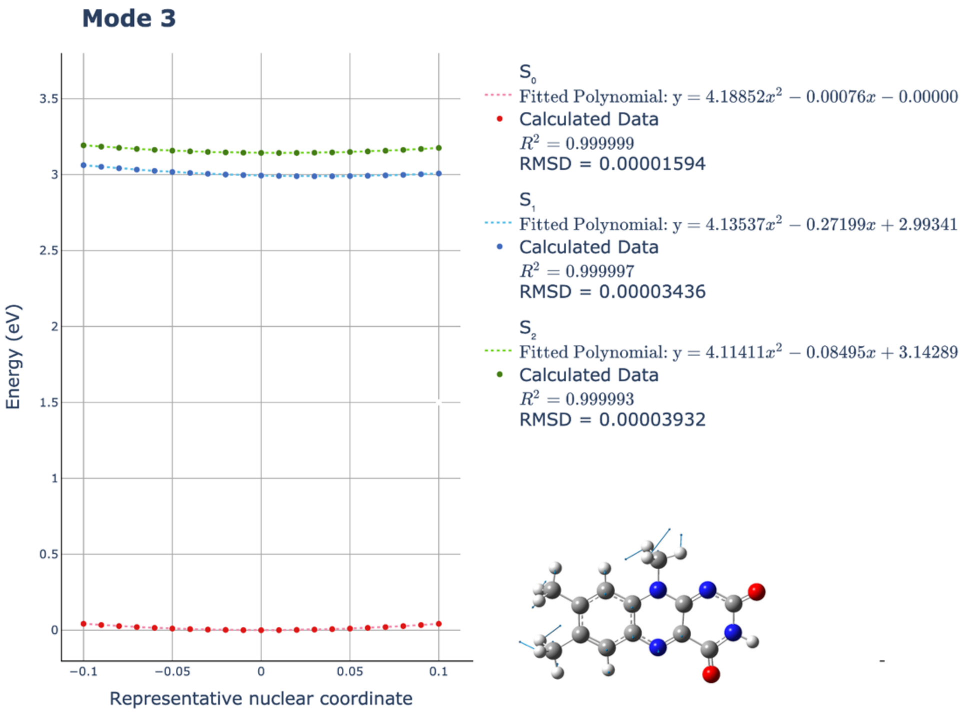

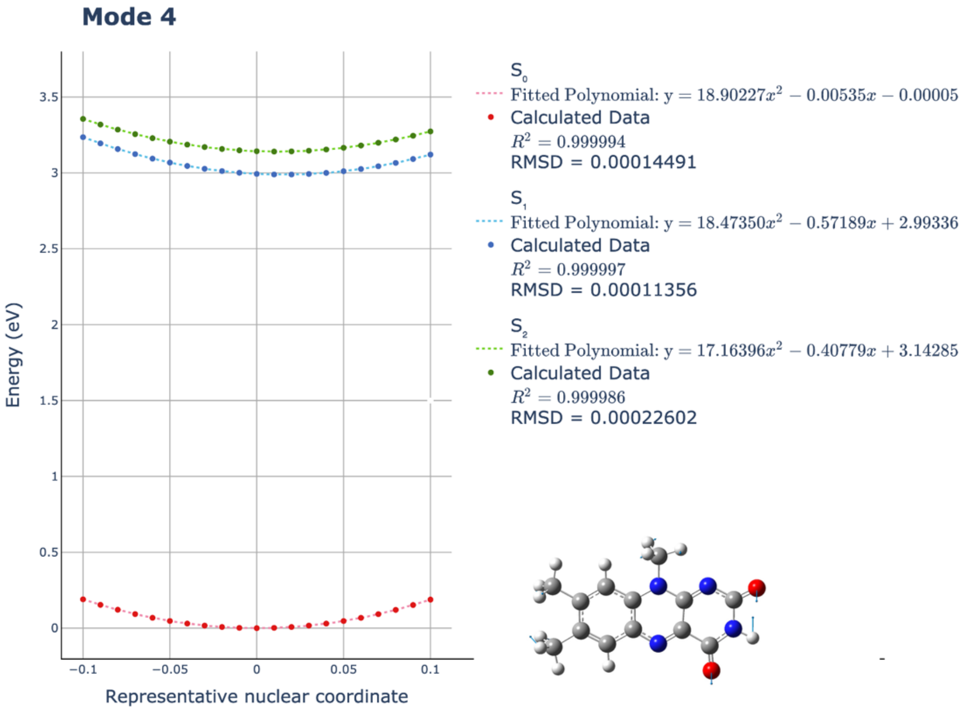

Fig. S5 (continued)

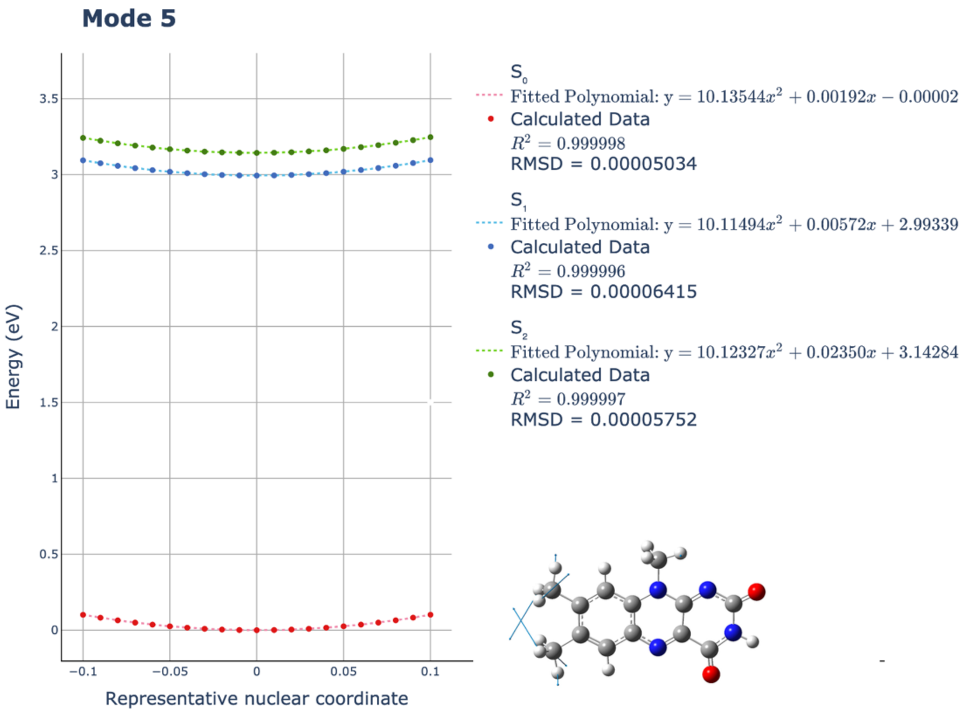

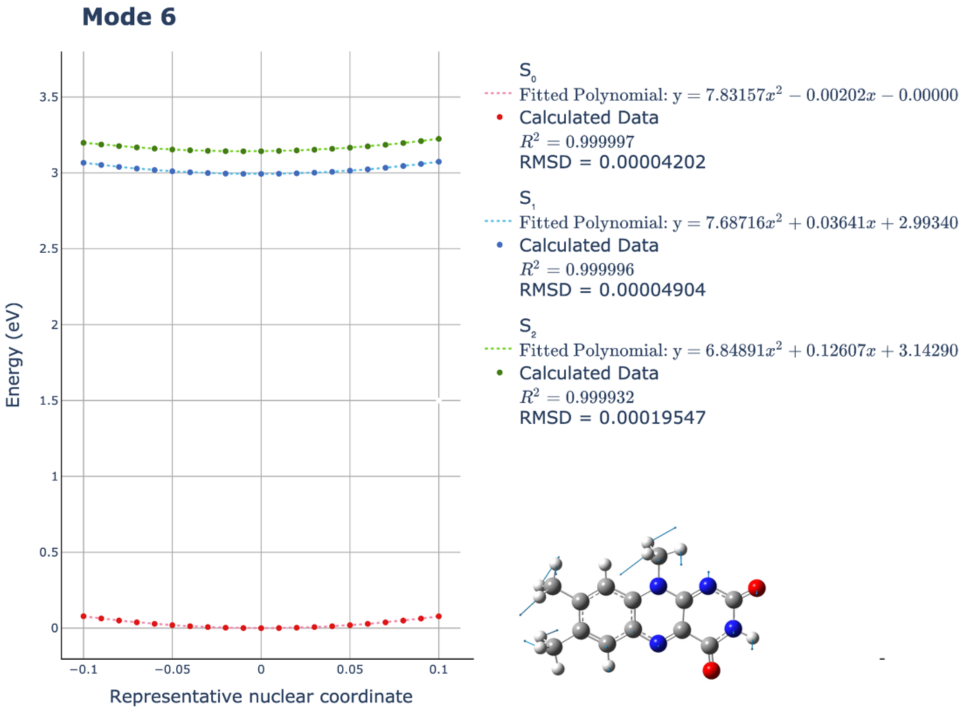

Fig. S5 (continued)

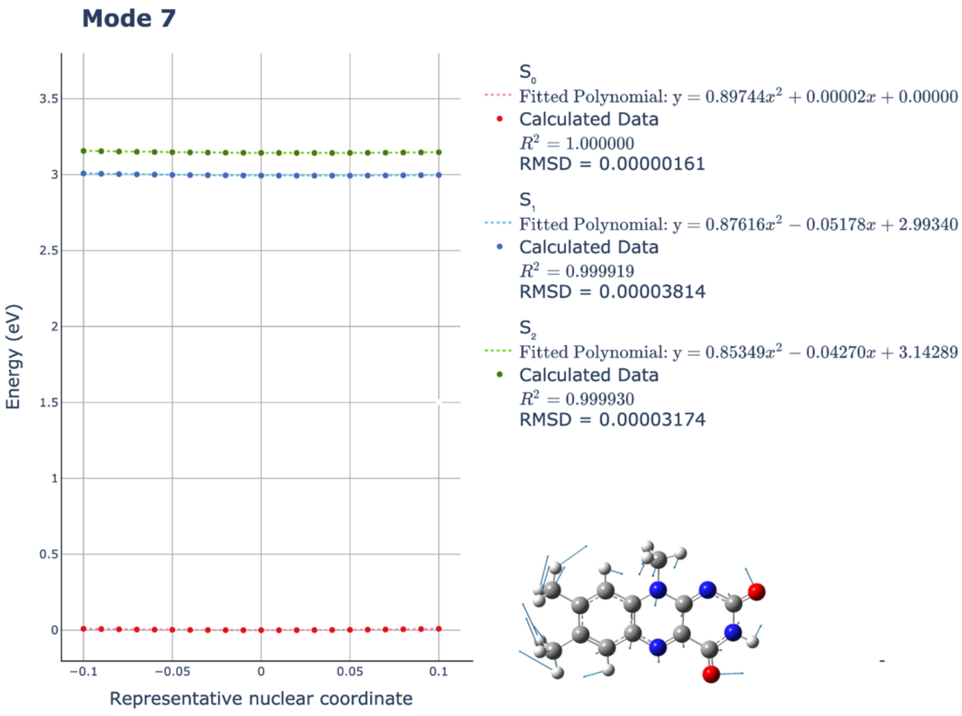

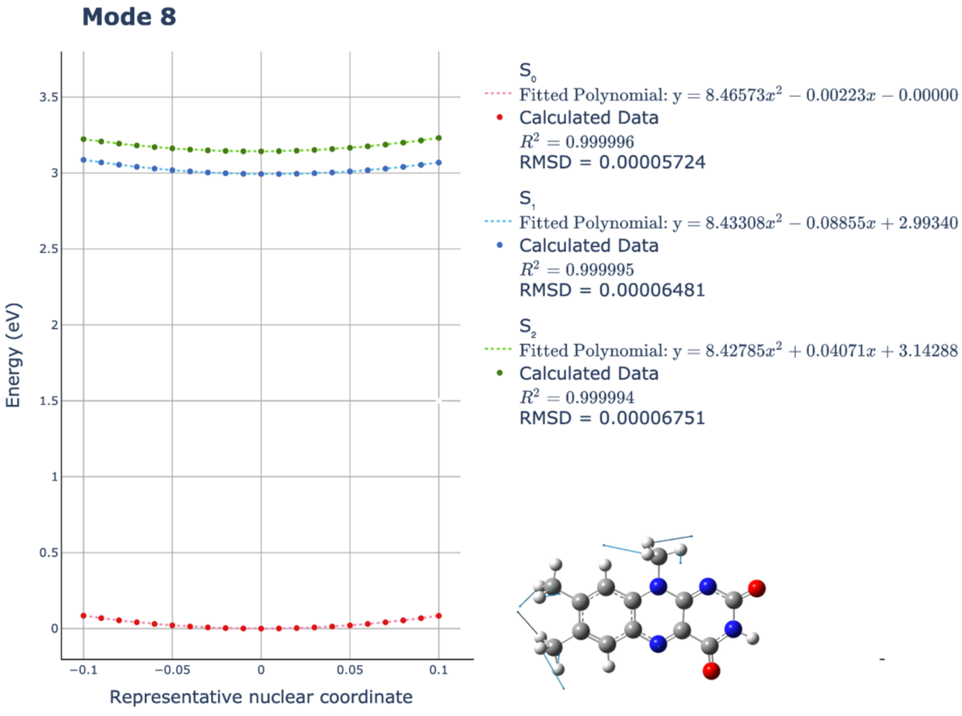

Fig. S5 (continued)

Mode 9

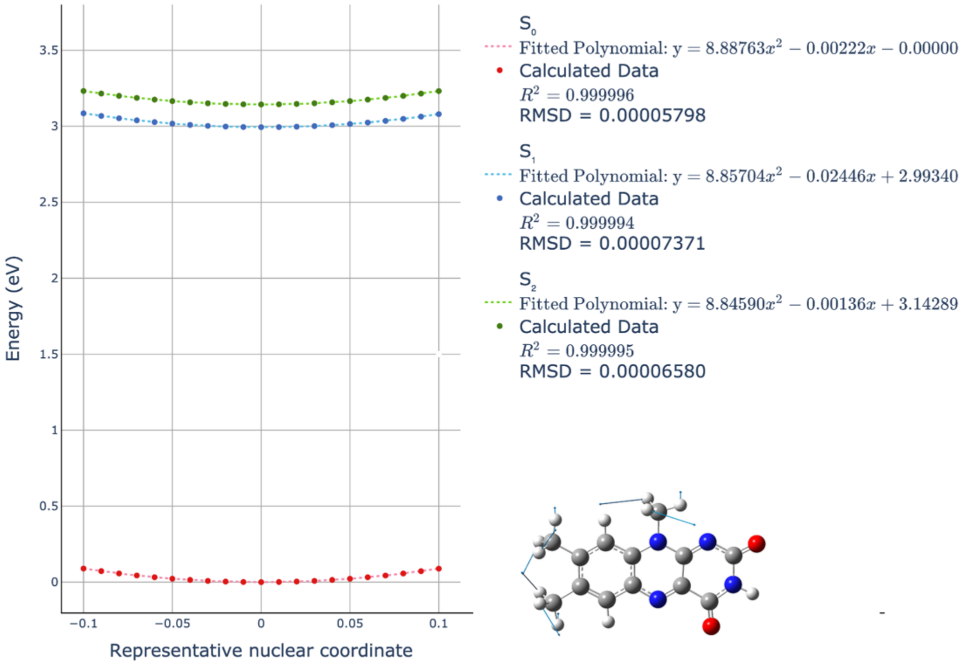

Mode 10

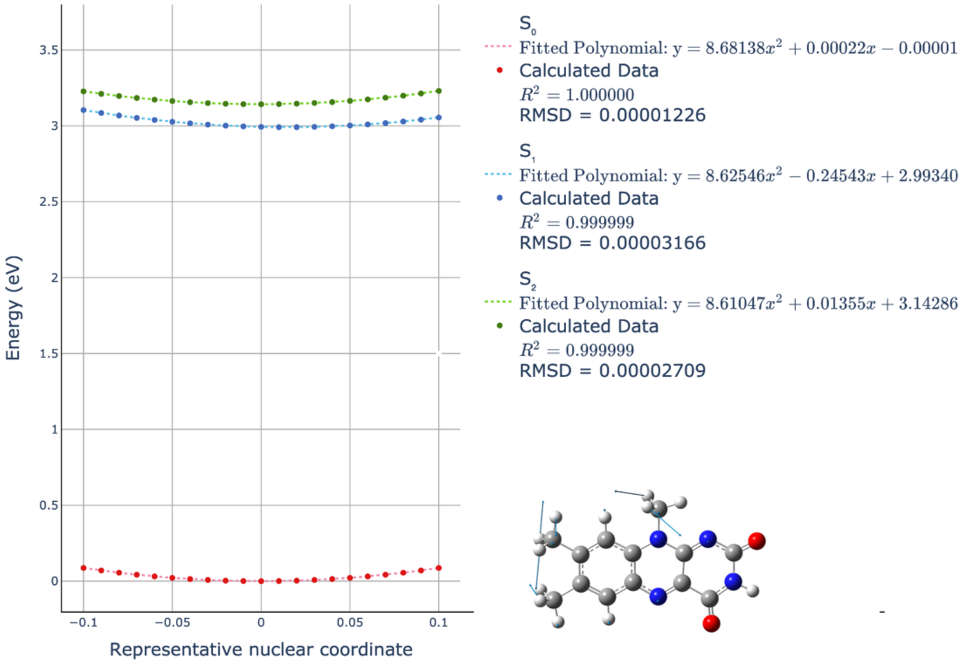

Fig. S5 (continued)

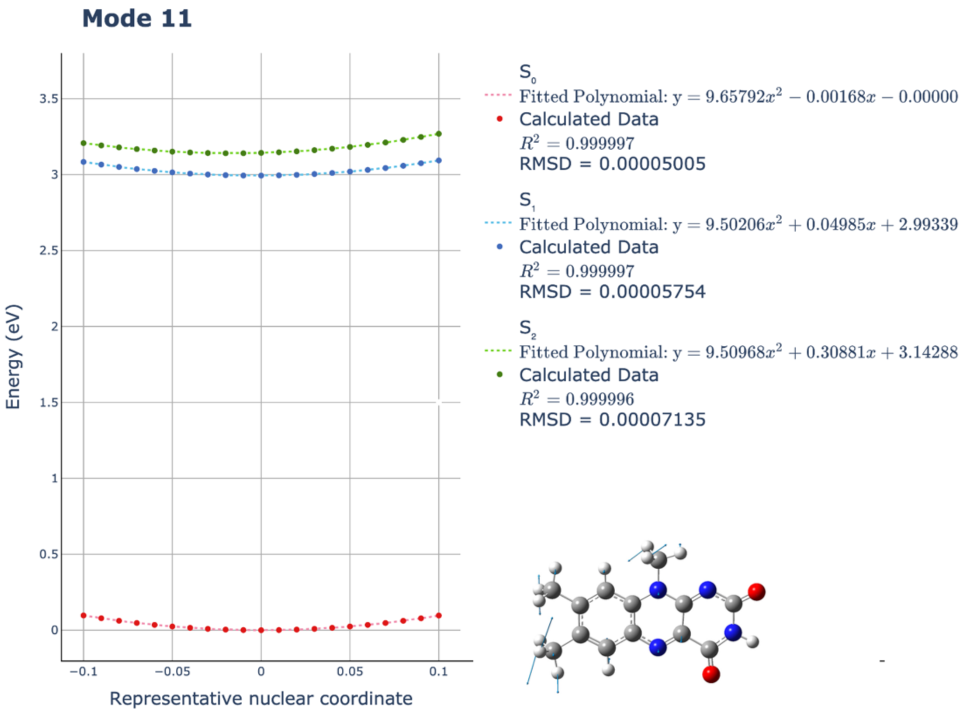

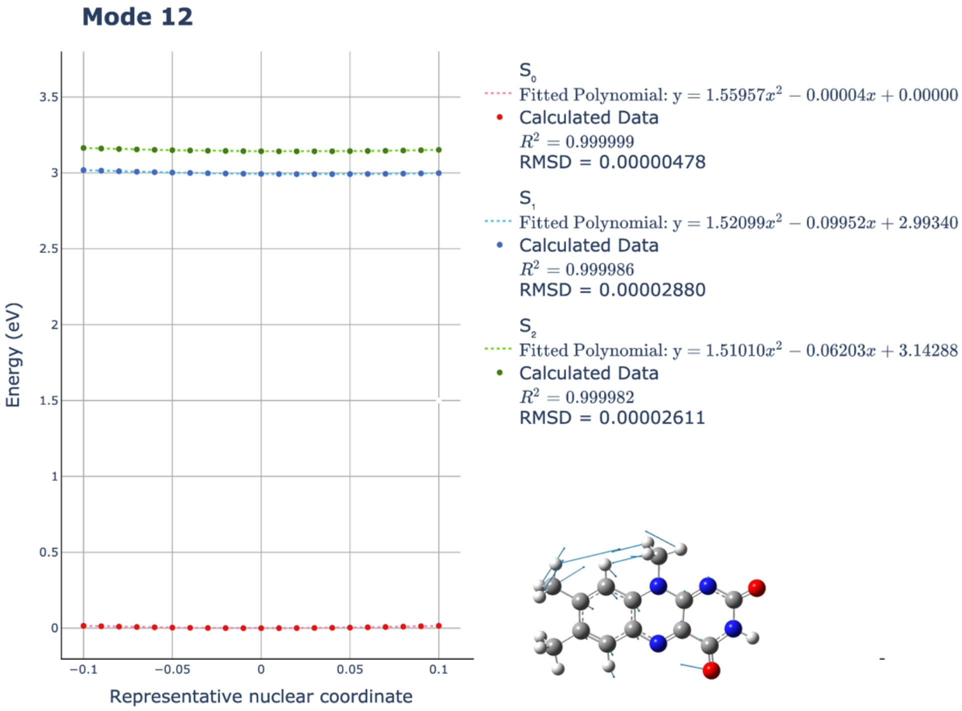

Fig. S5 (continued)

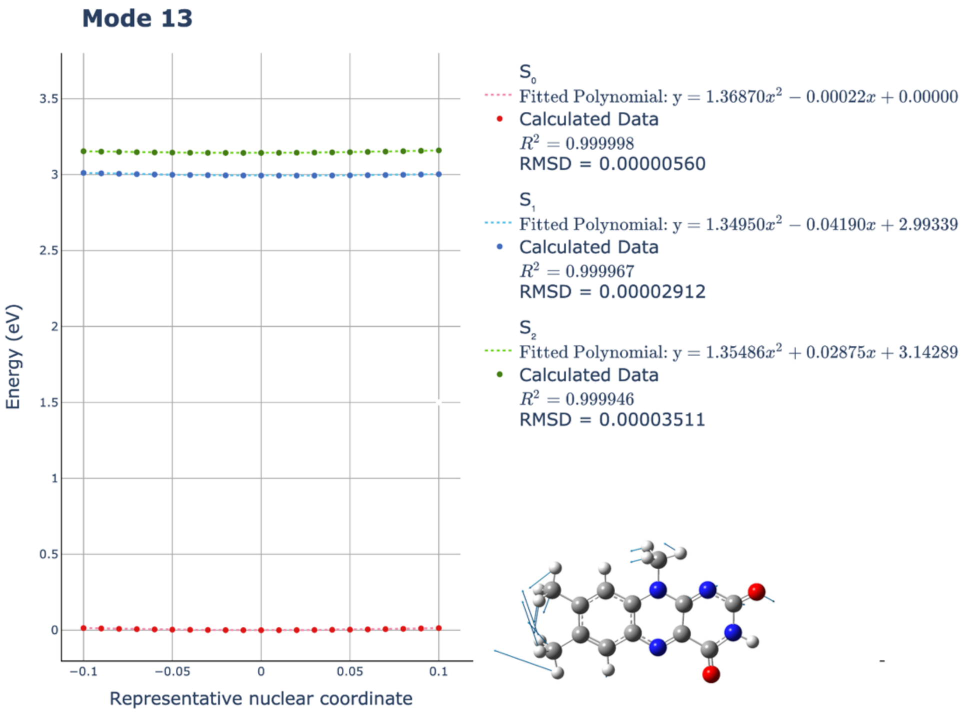

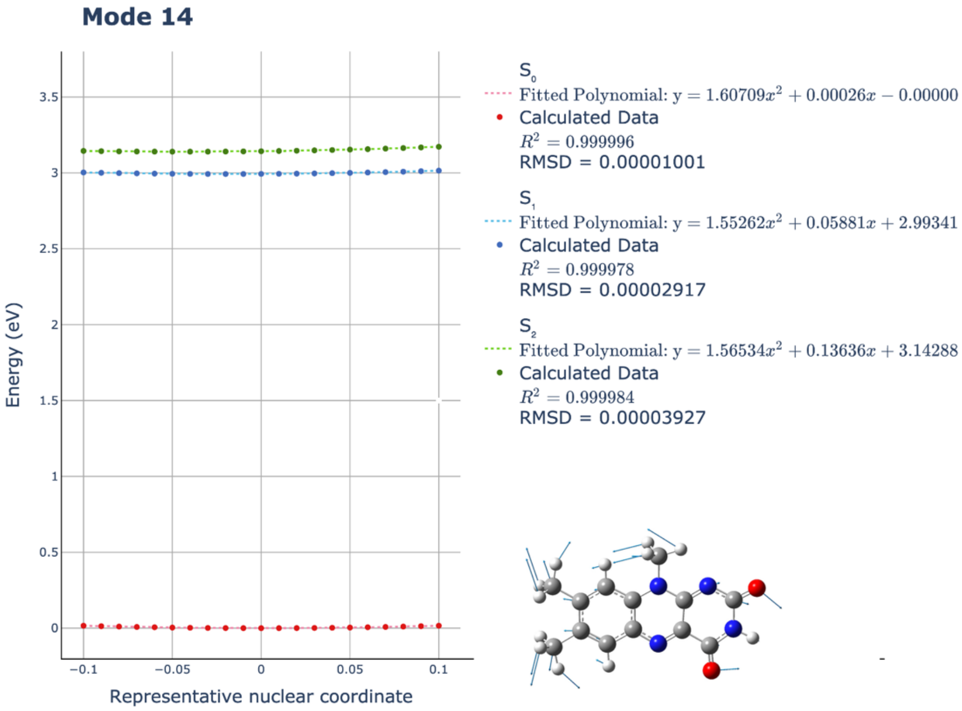

Fig. S5 (continued)

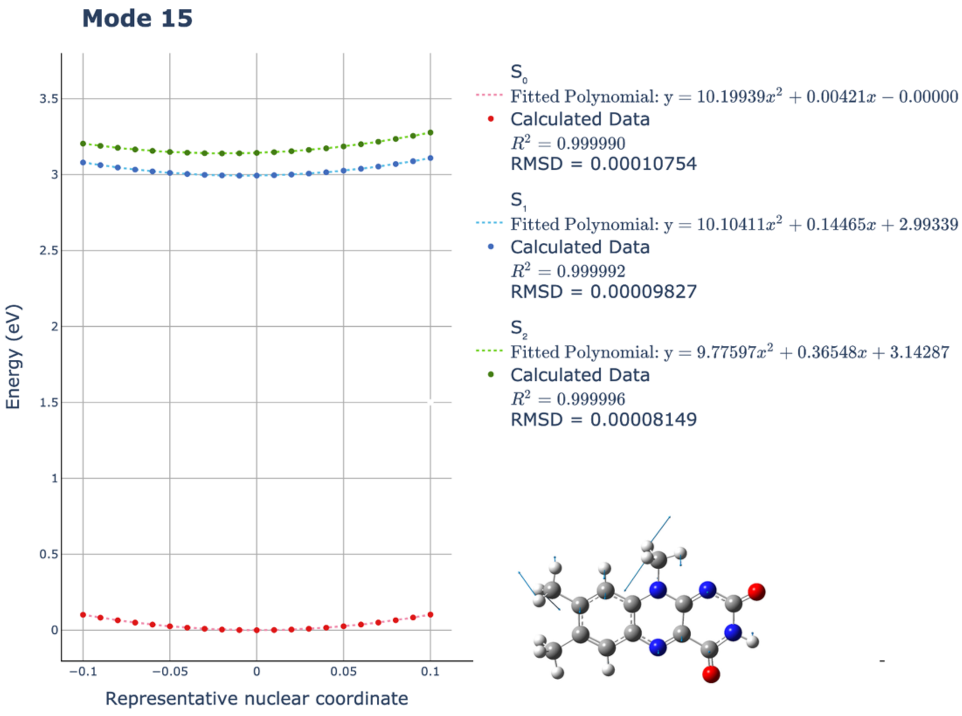

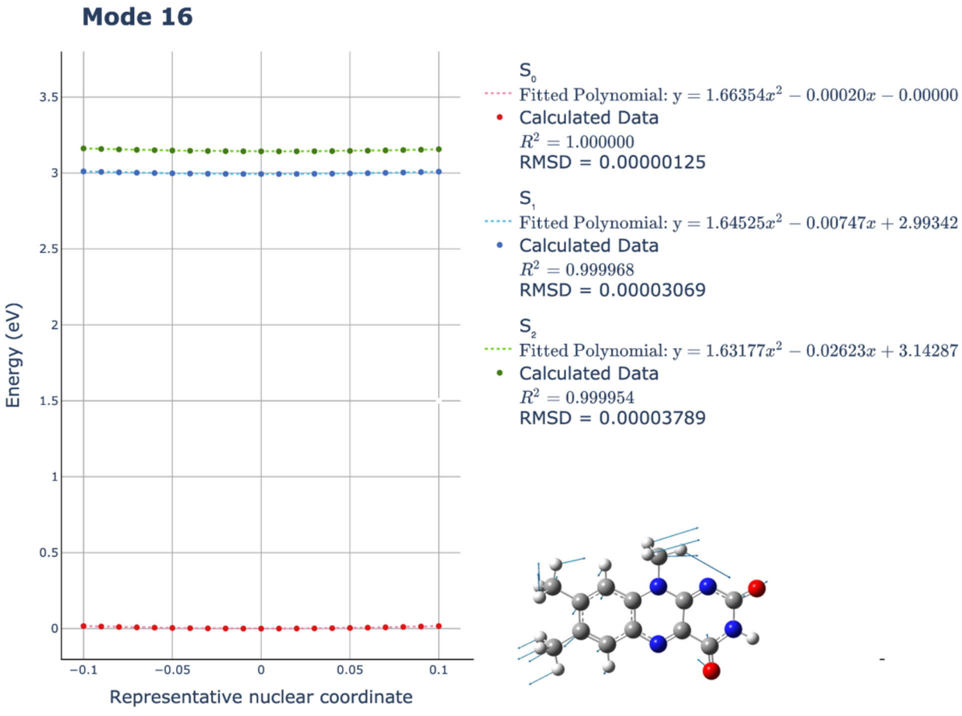

Fig. S5 (continued)

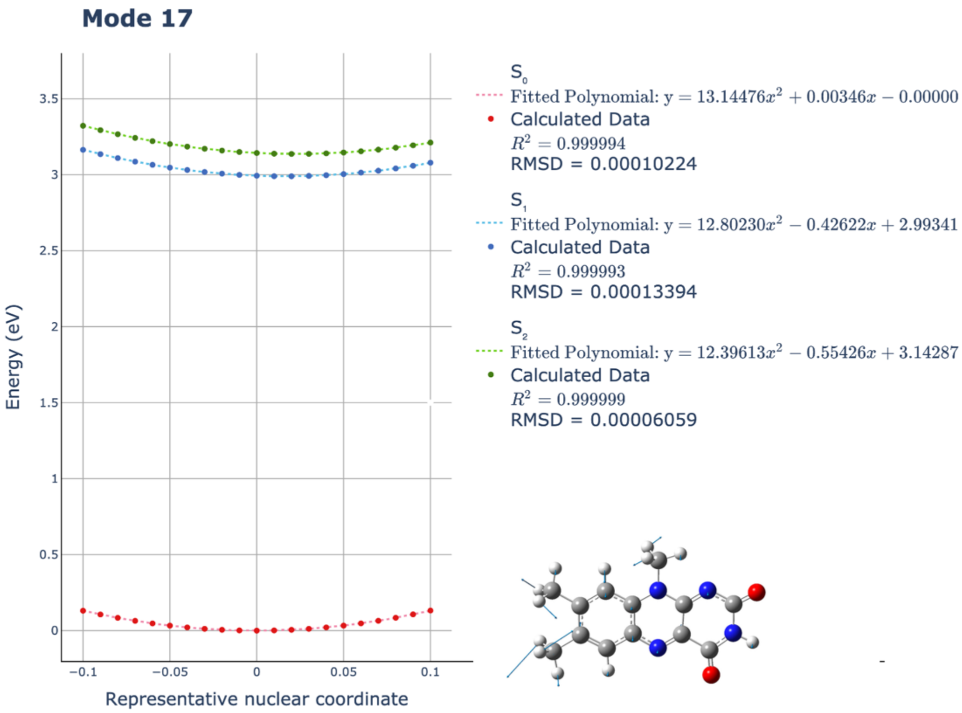

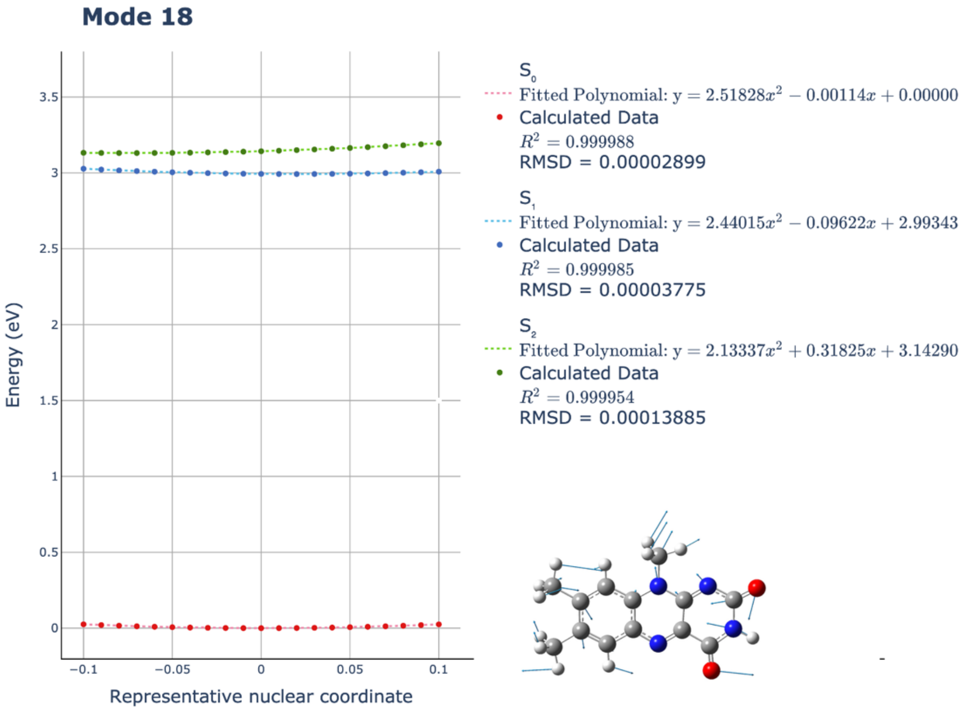

Fig. S5 (continued)

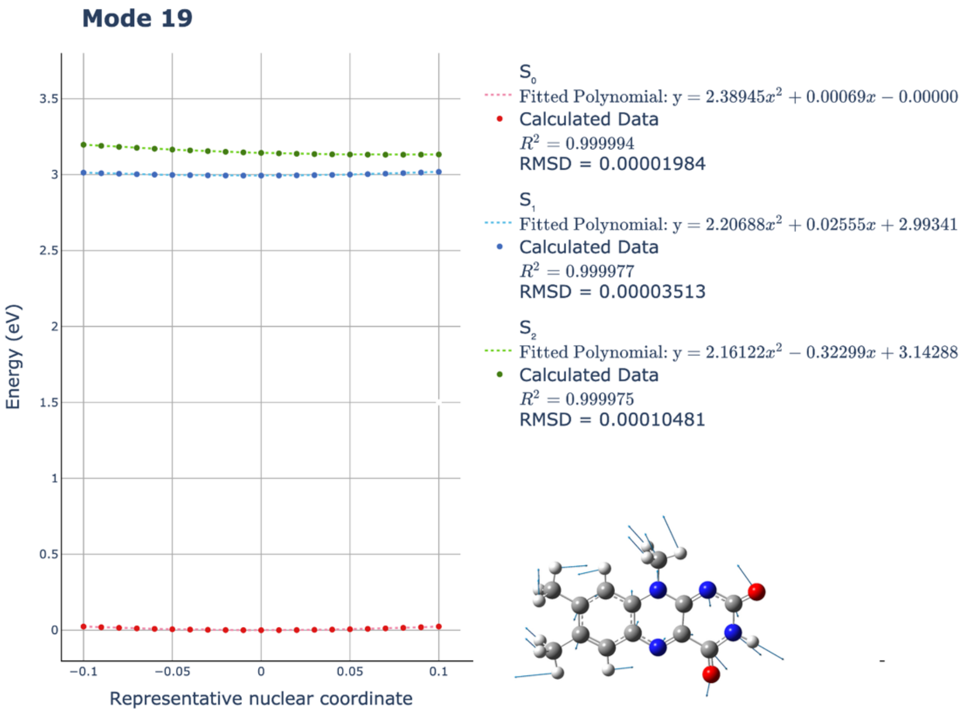

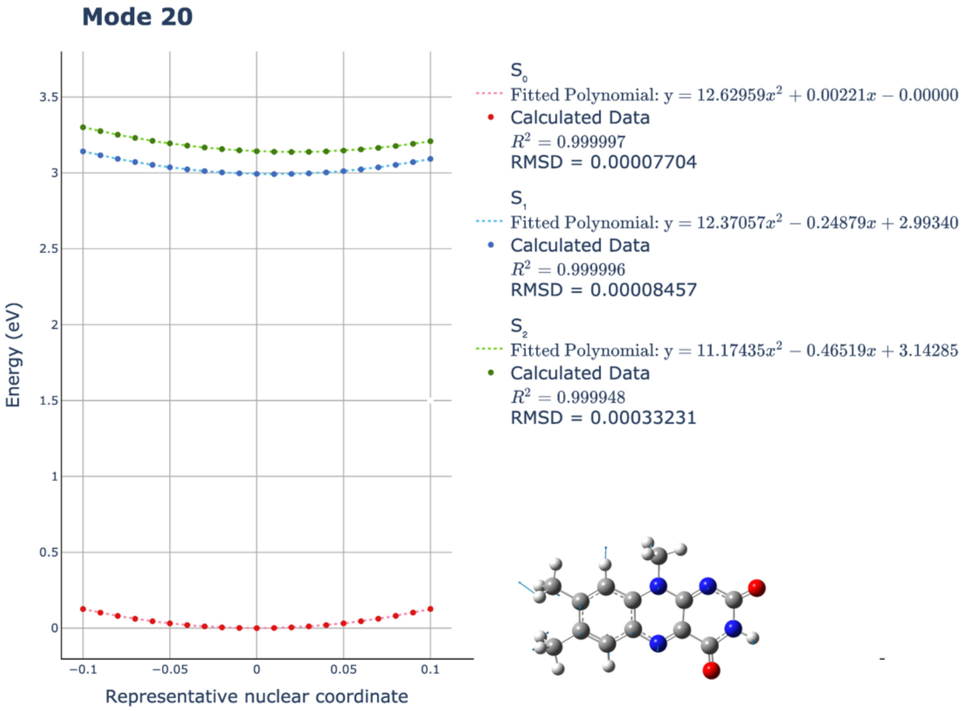

Fig. S5 (continued)

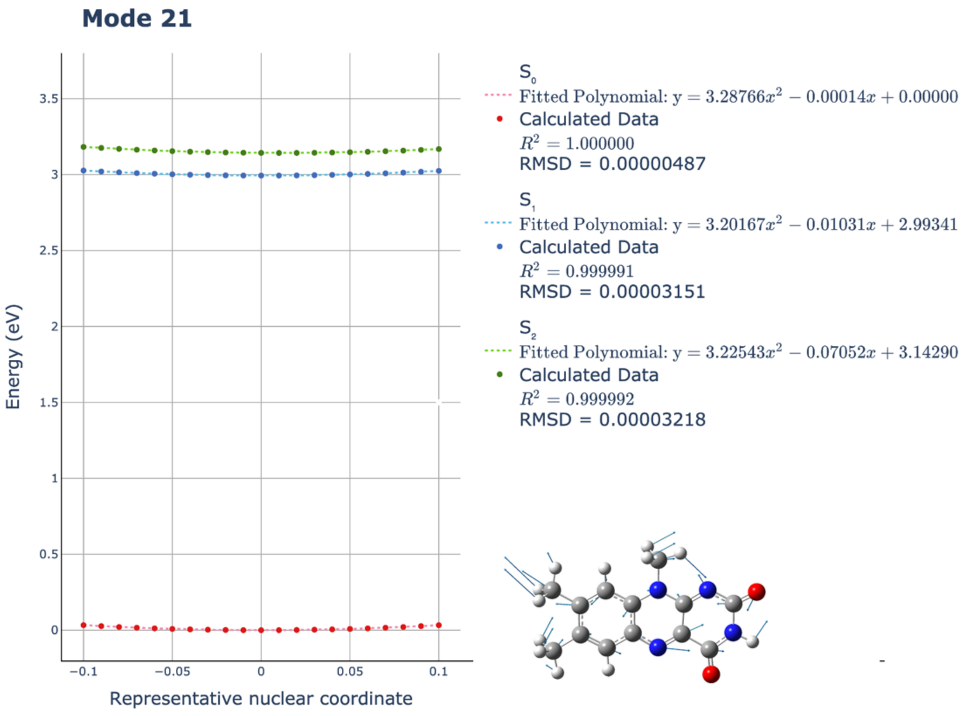

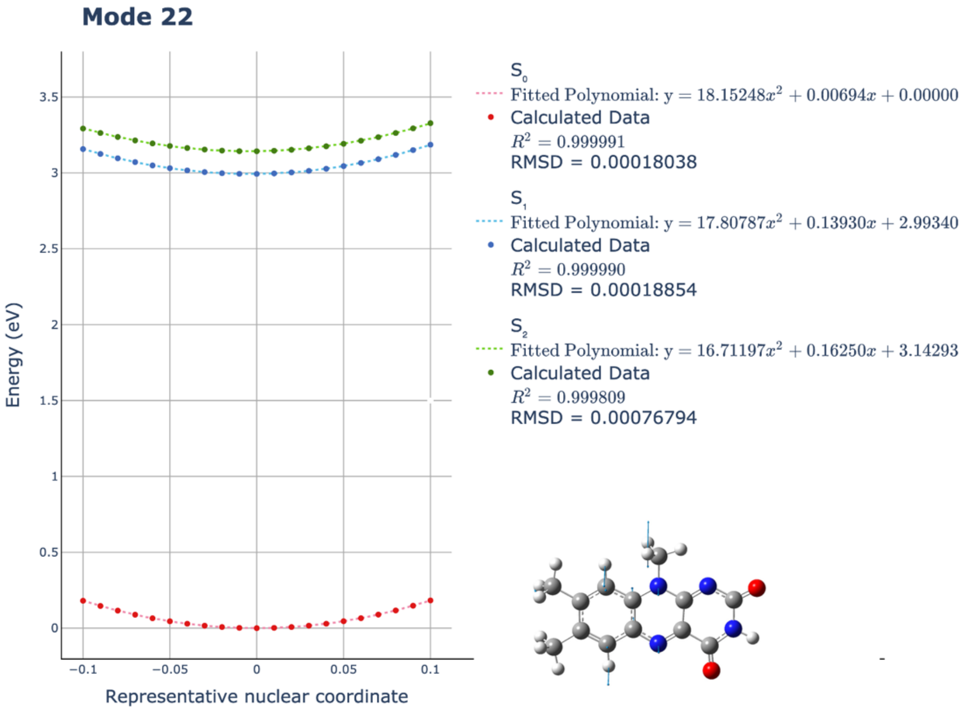

Fig. S5 (continued)

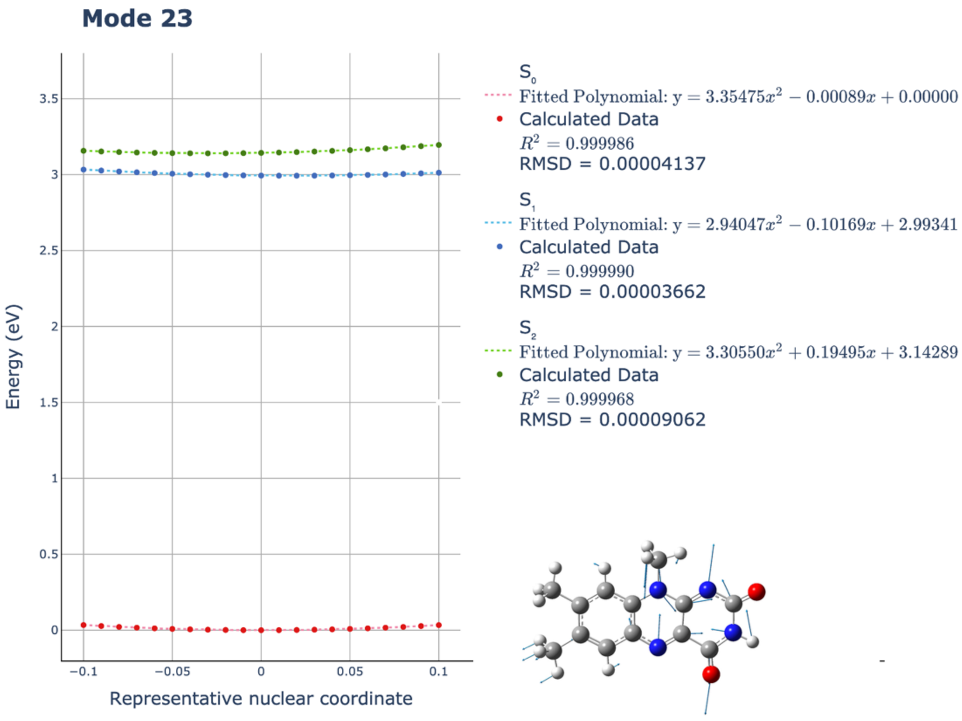

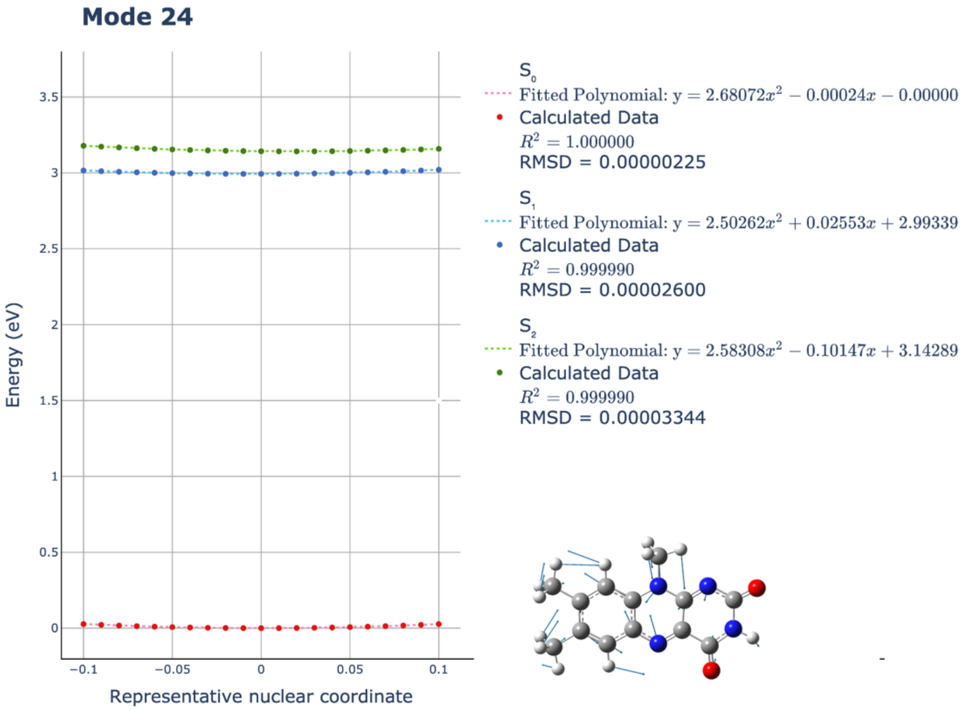

Fig. S5 (continued)

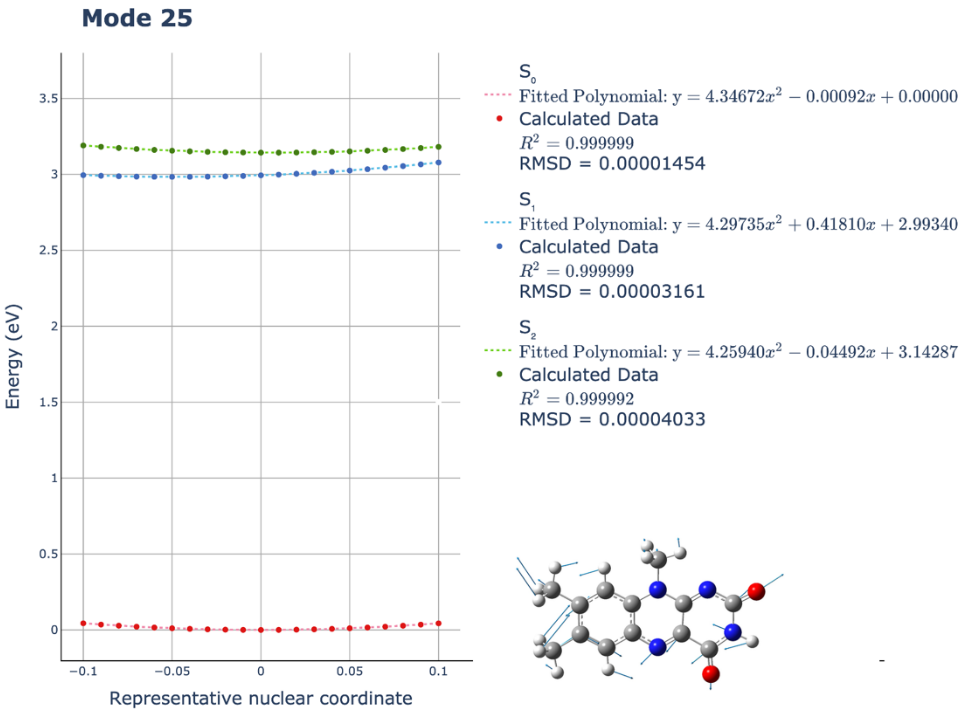

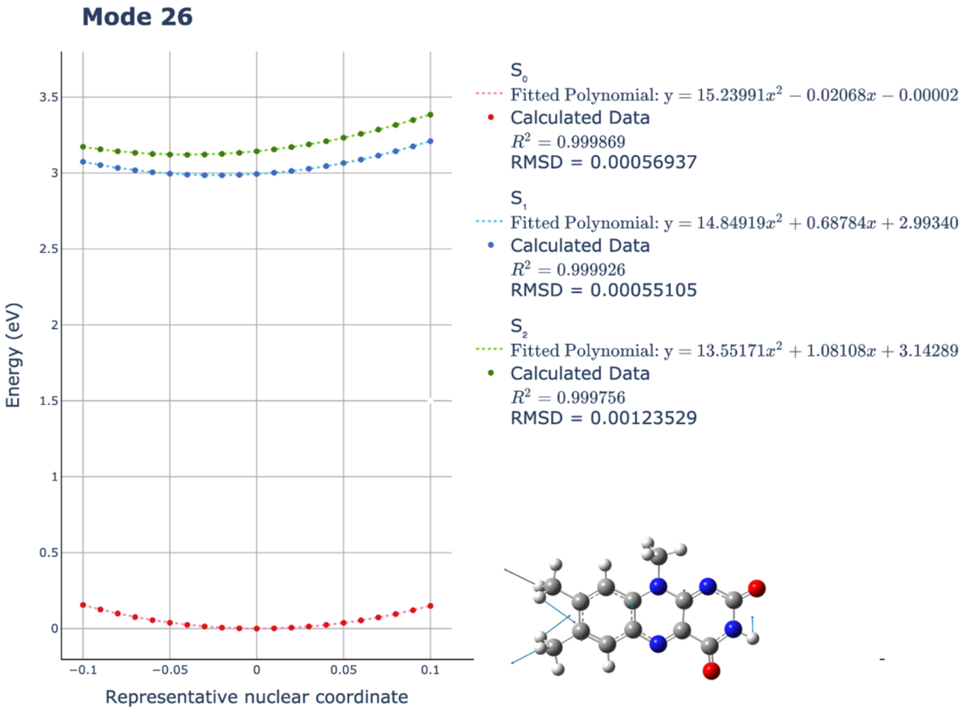

Fig. S5 (continued)

Mode 27

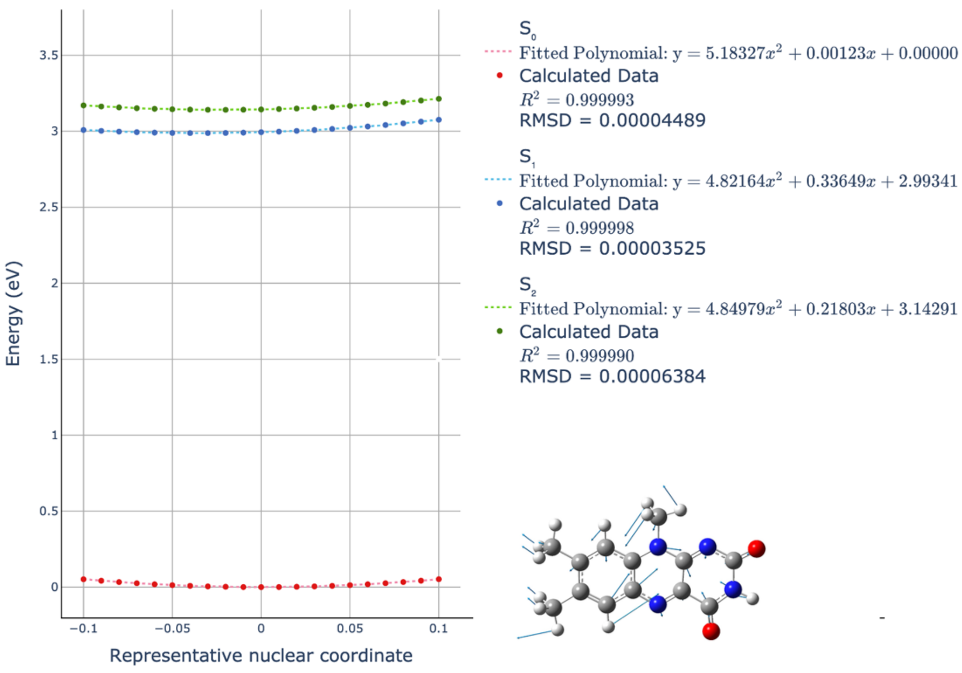

Mode 28

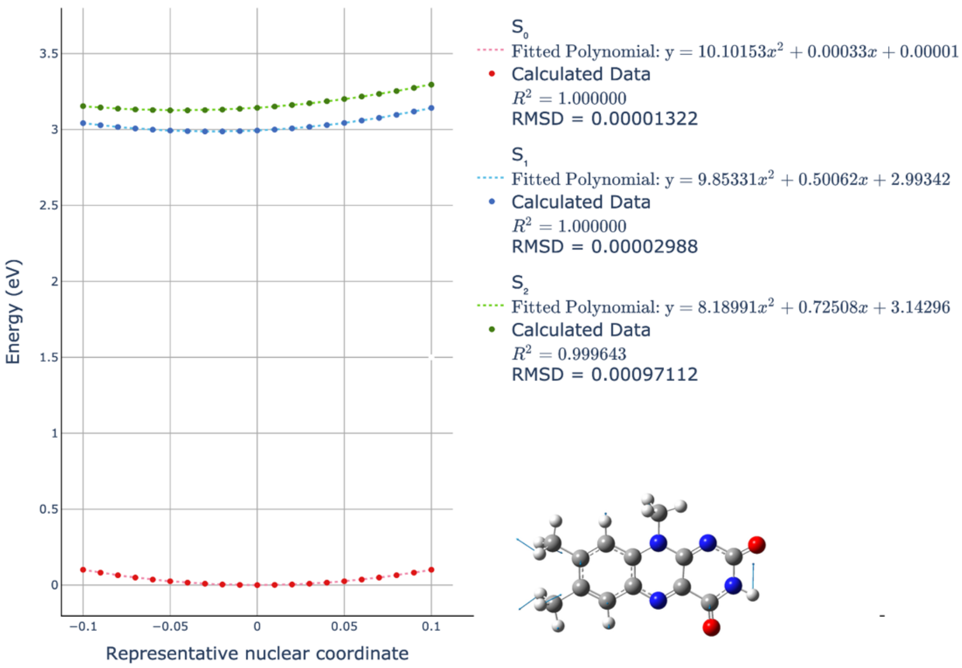

Fig. S5 (continued)

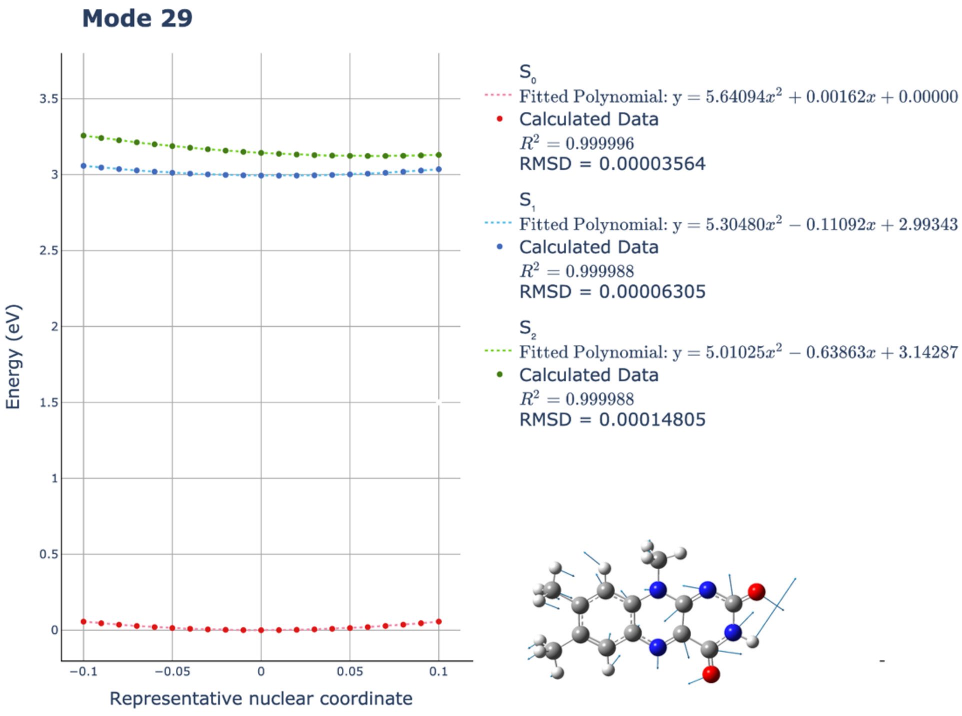

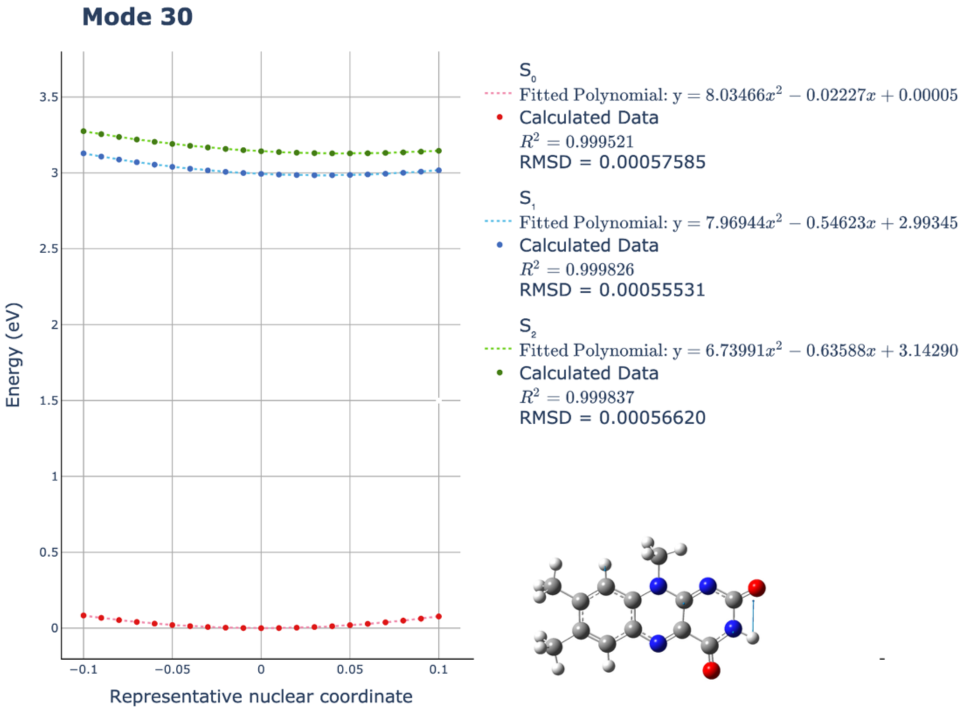

Fig. S5 (continued)

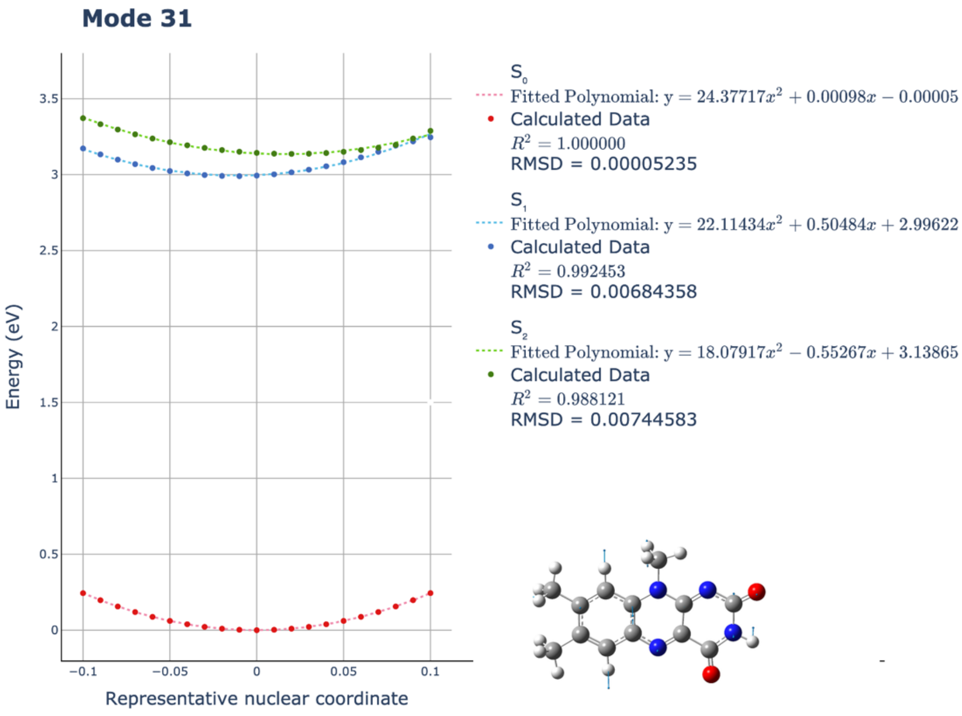

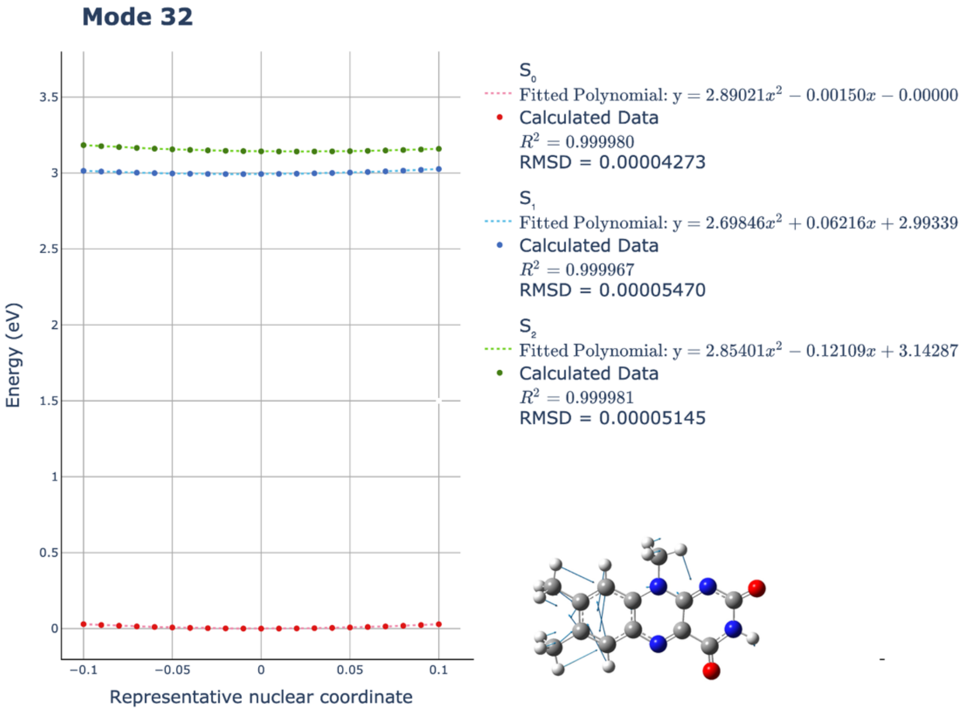

Fig. S5 (continued)

Mode 33

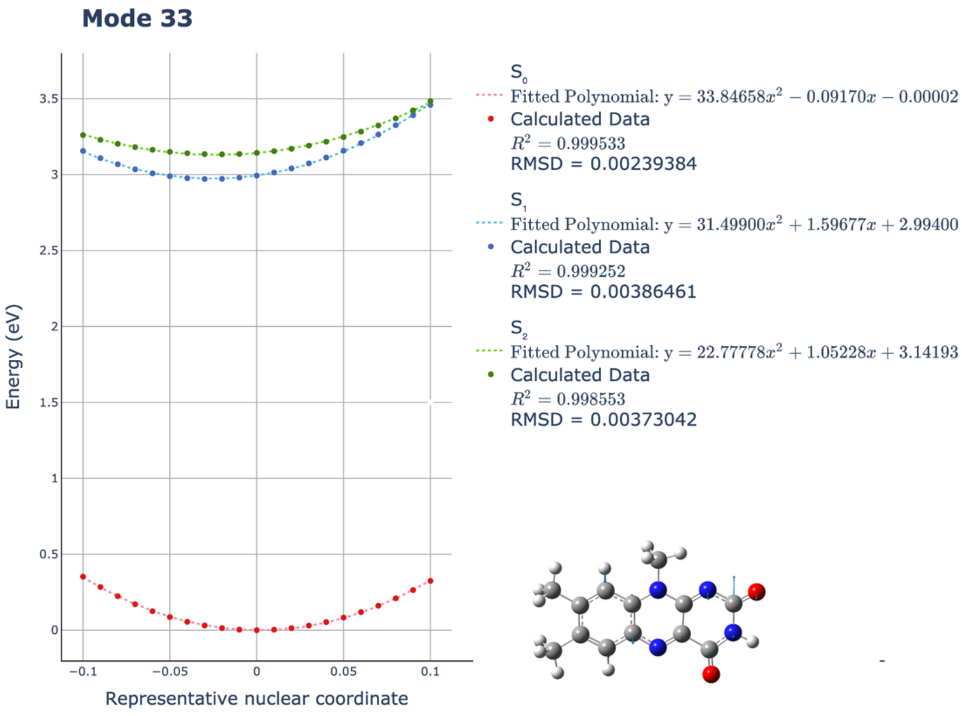

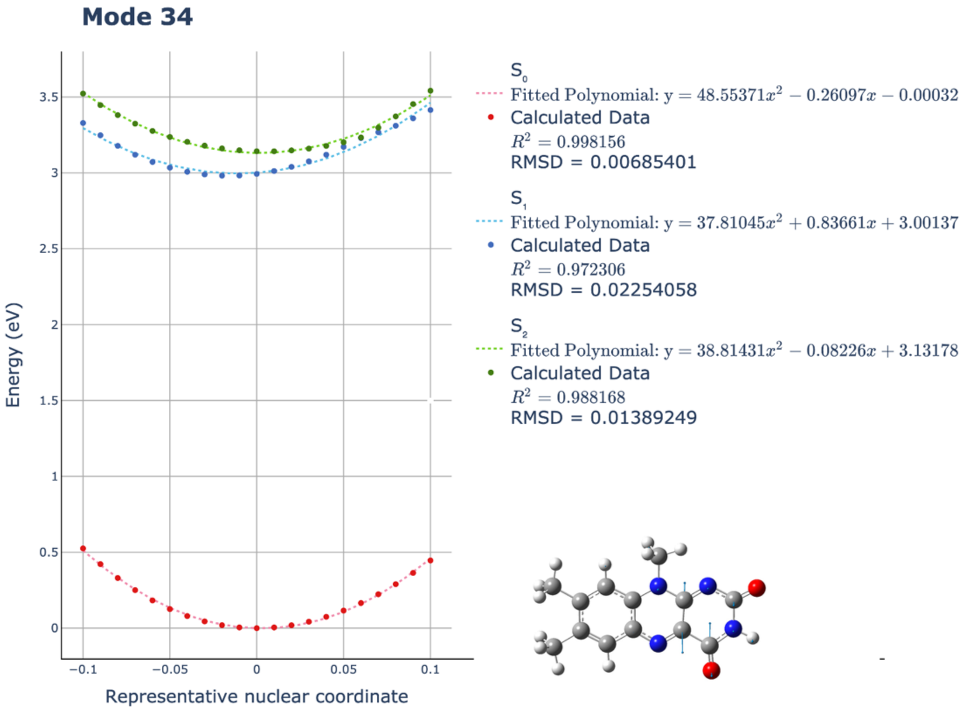

Fig. S5 (continued)

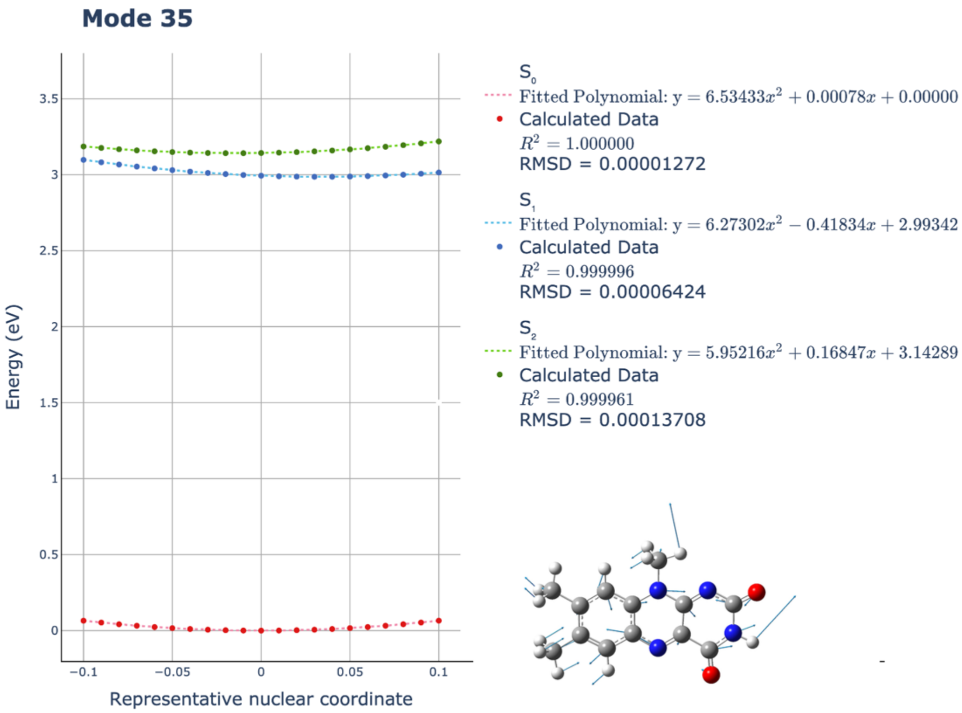

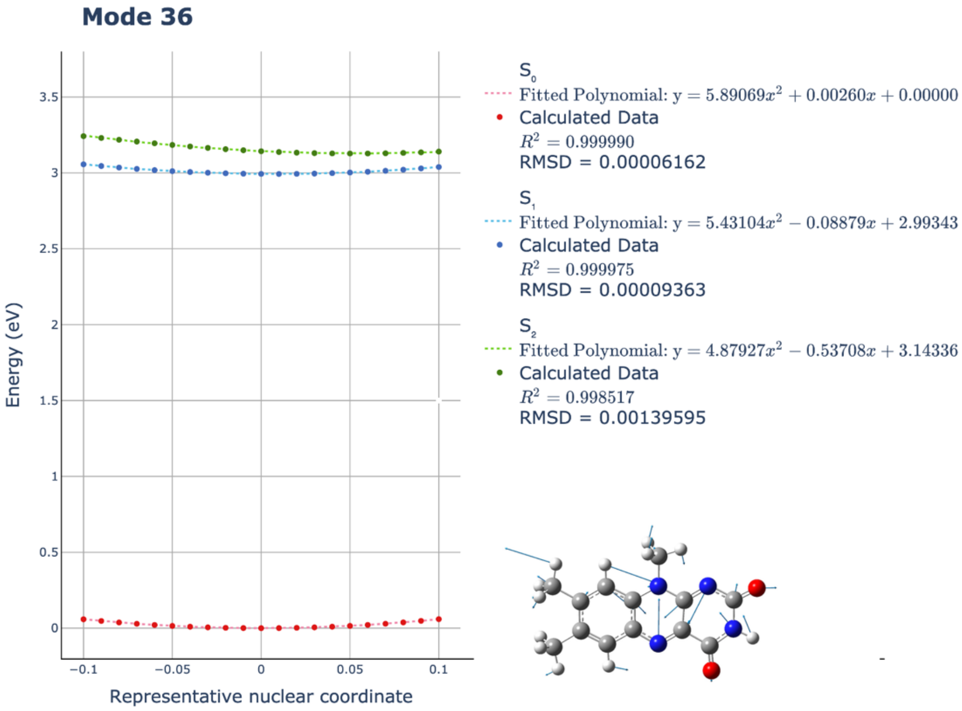

Fig. S5 (continued)

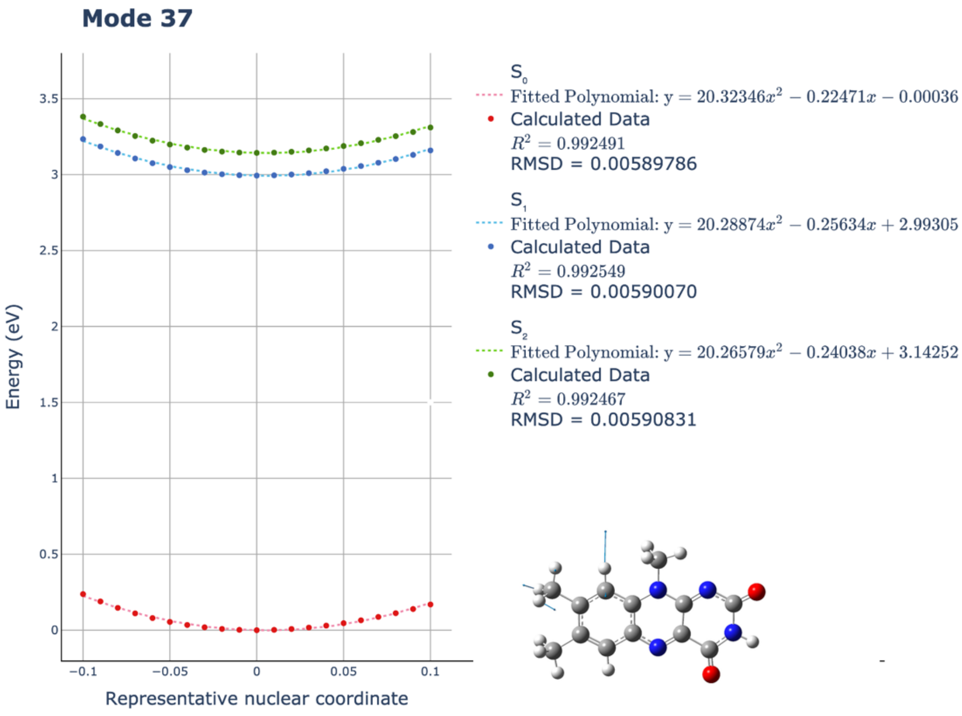

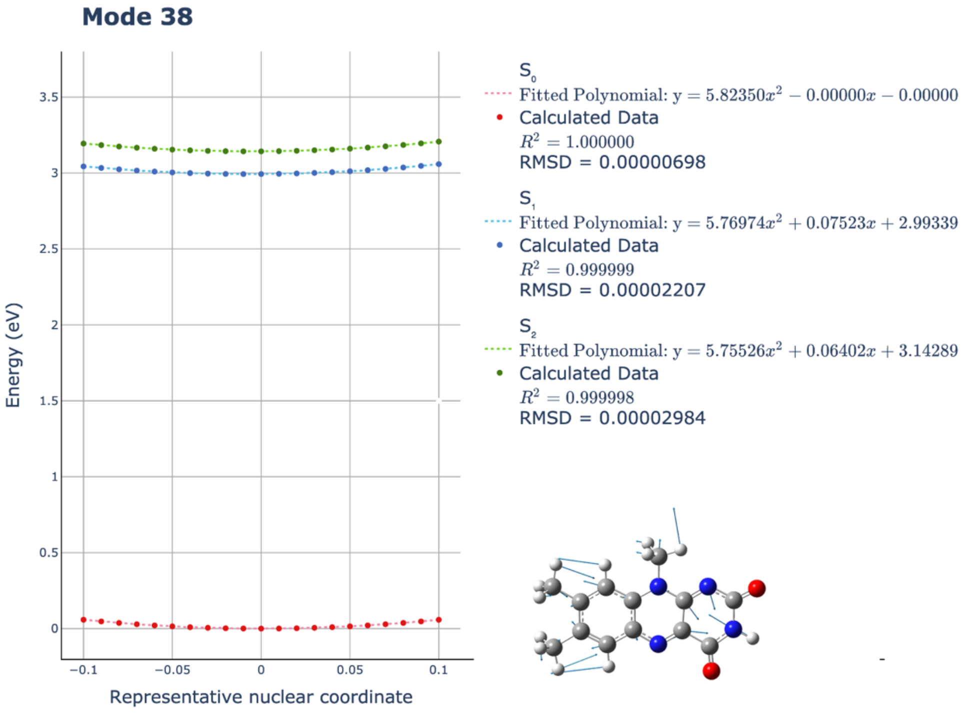

Fig. S5 (continued)

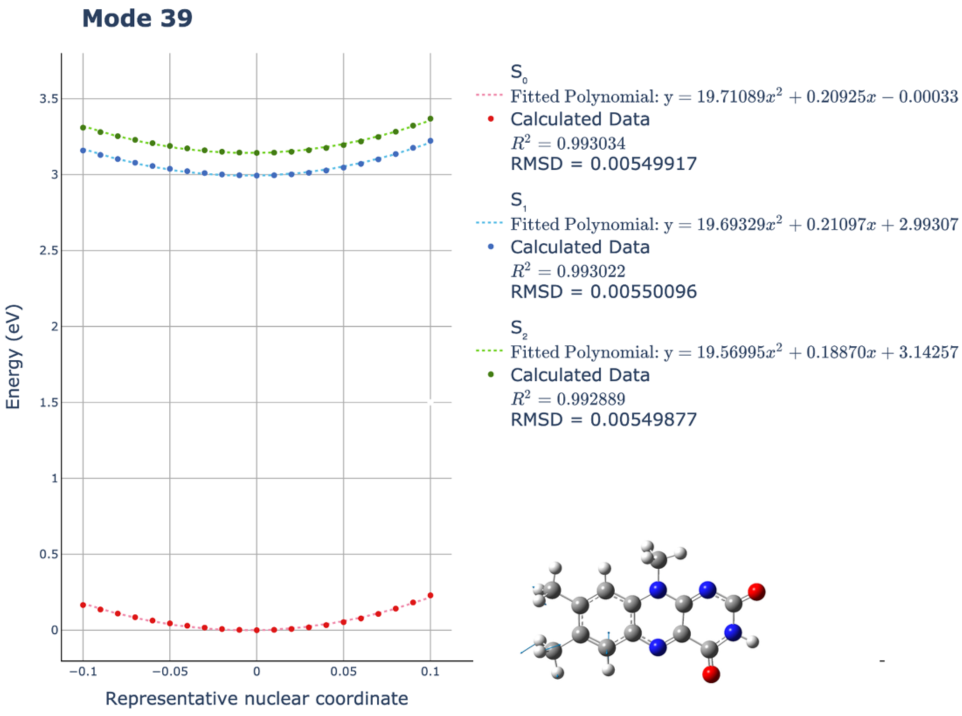

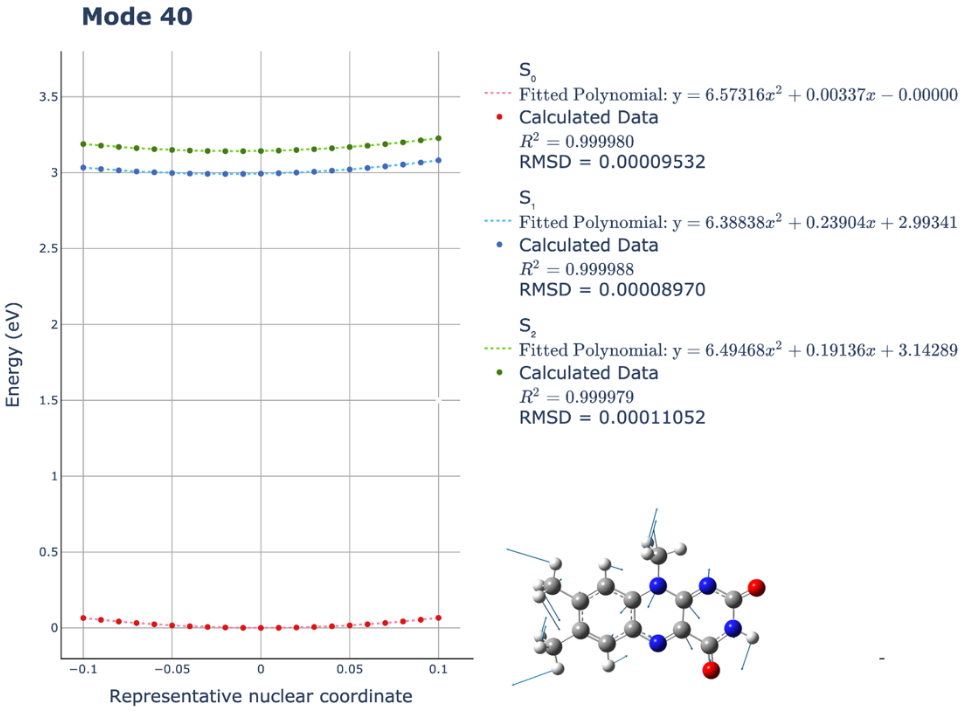

Fig. S5 (continued)

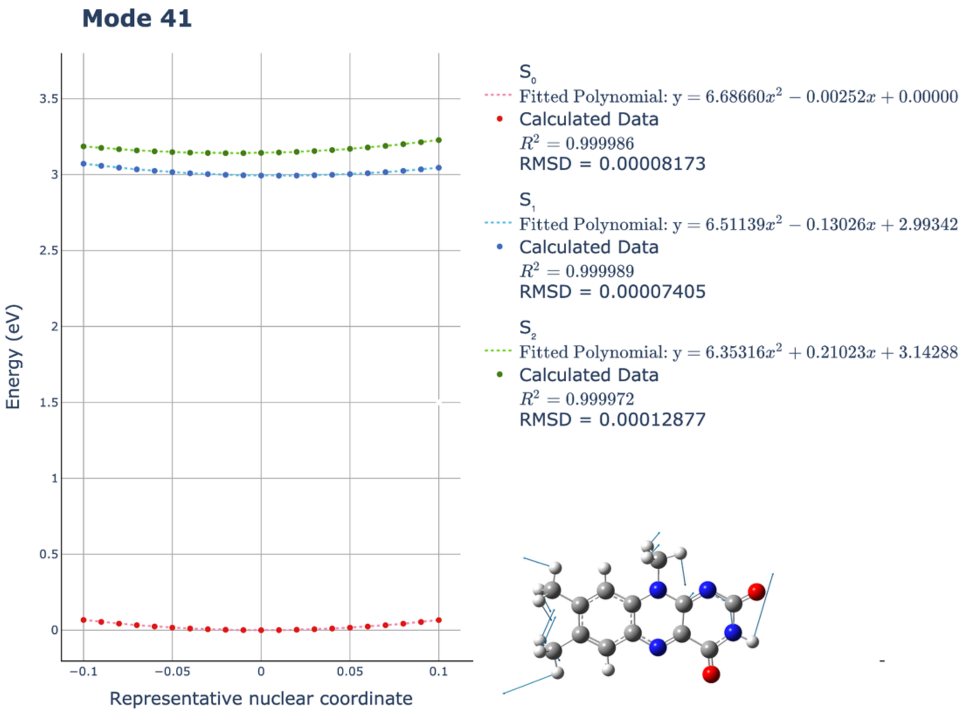

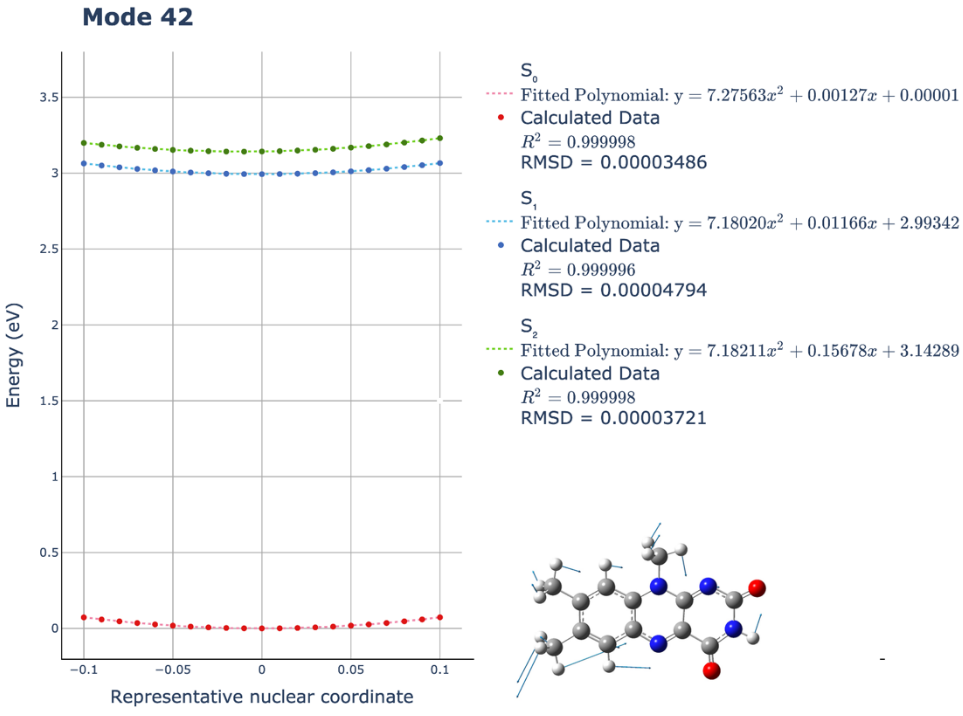

Fig. S5 (continued)

Mode 43

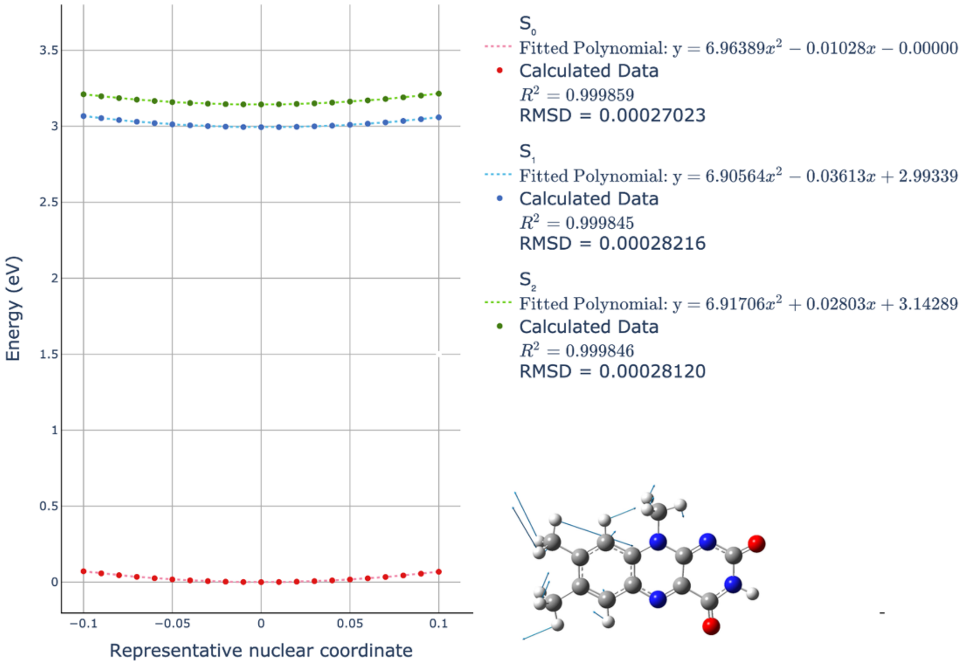

Mode 44

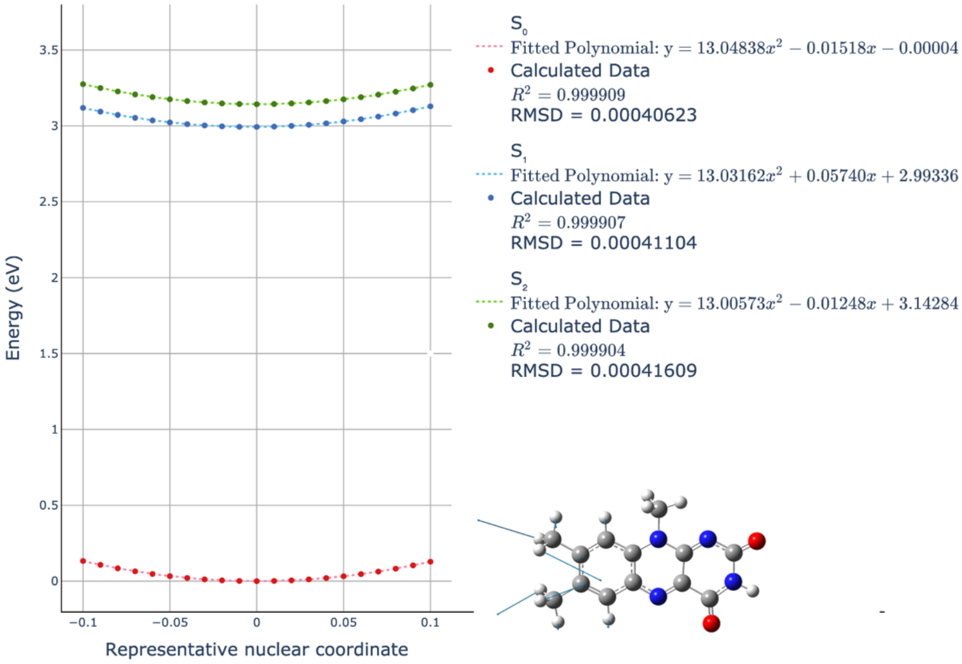

Fig. S5 (continued)

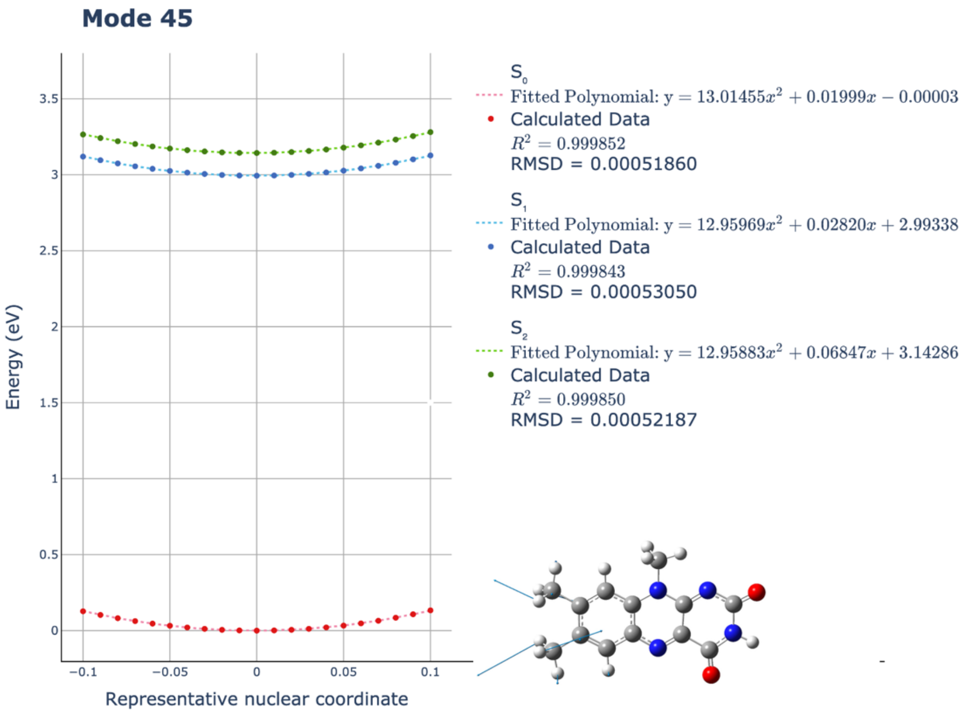

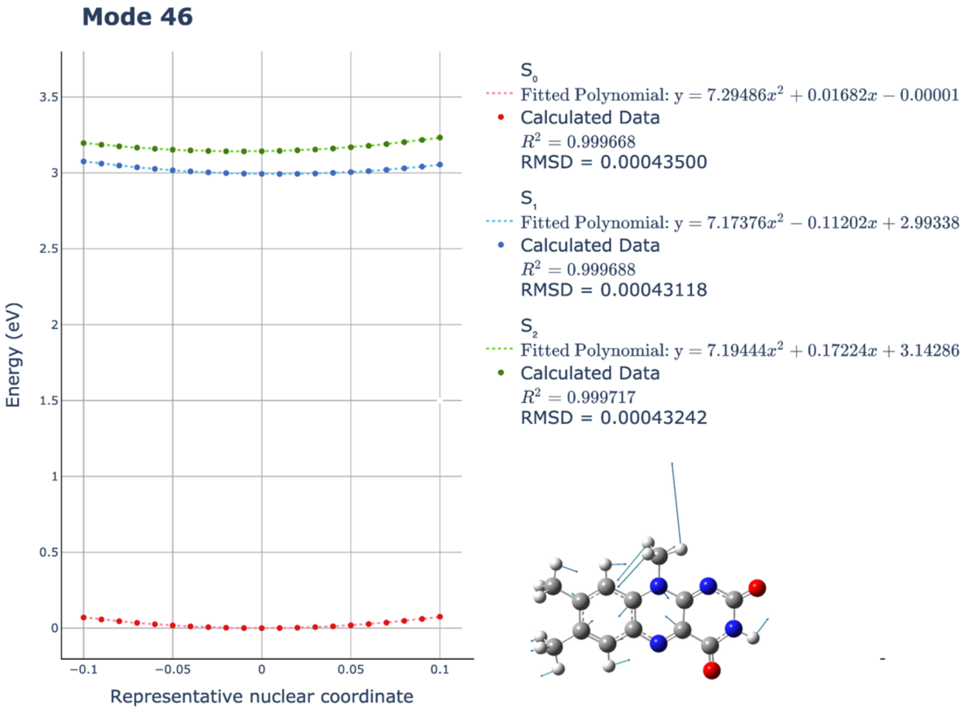

Fig. S5 (continued)

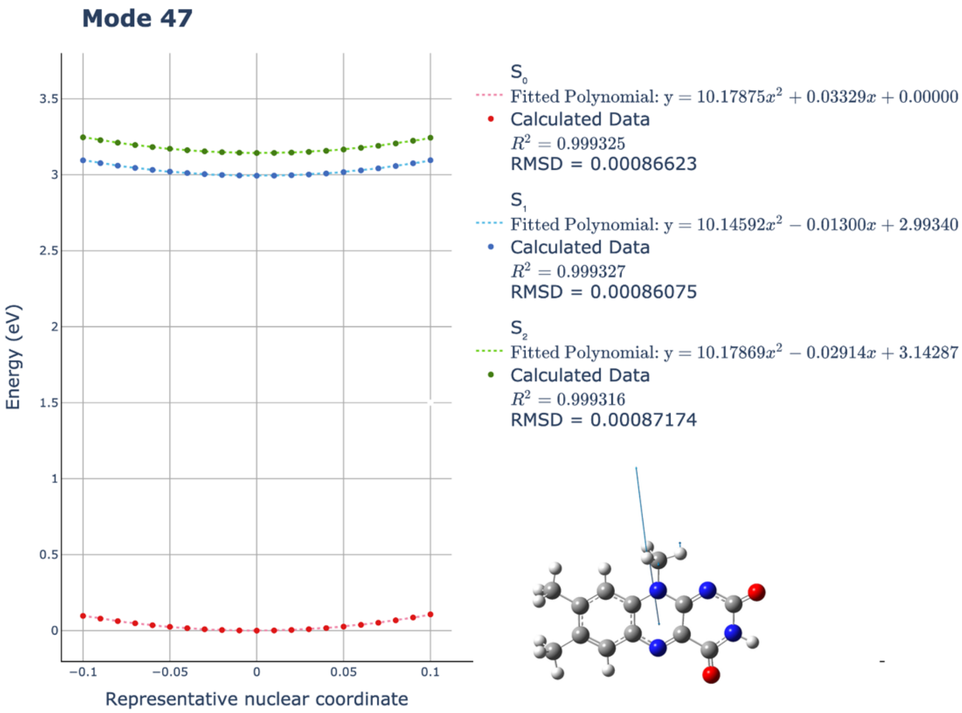

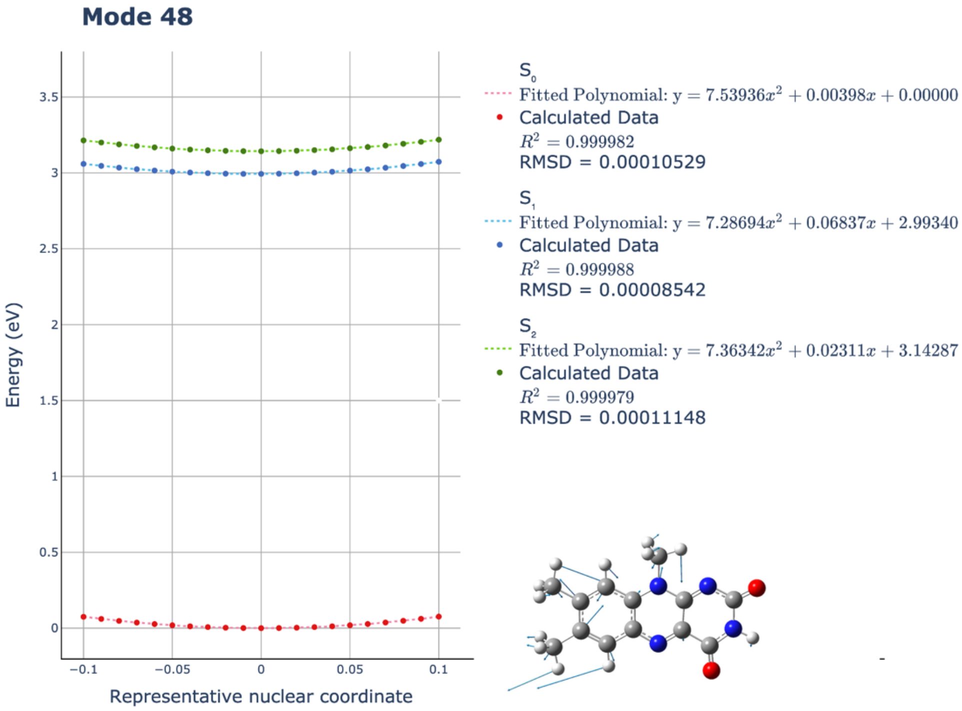

Fig. S5 (continued)

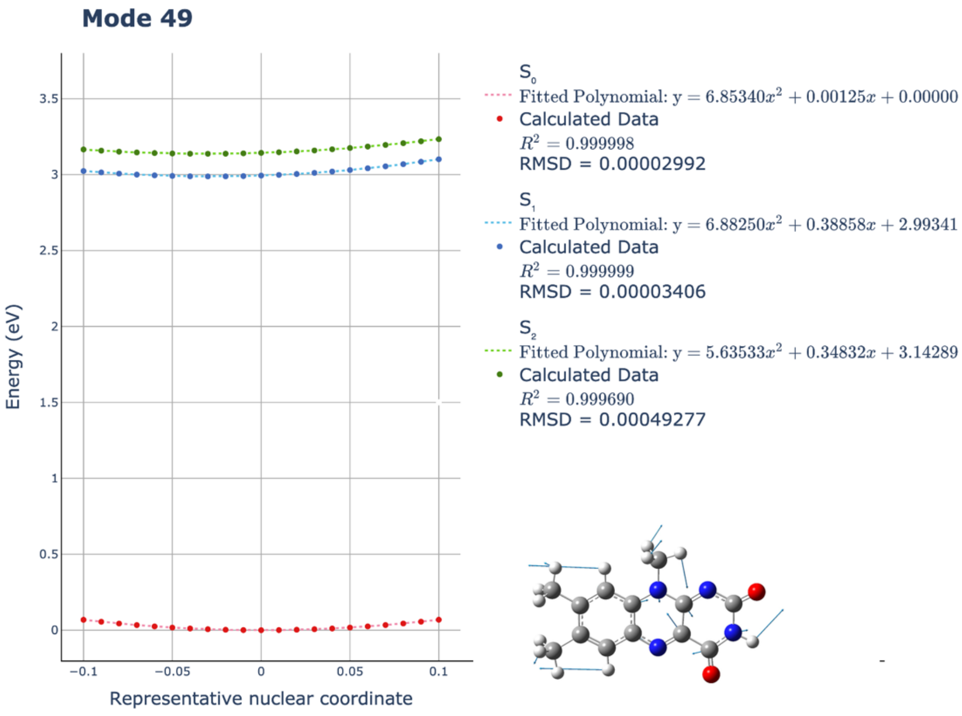

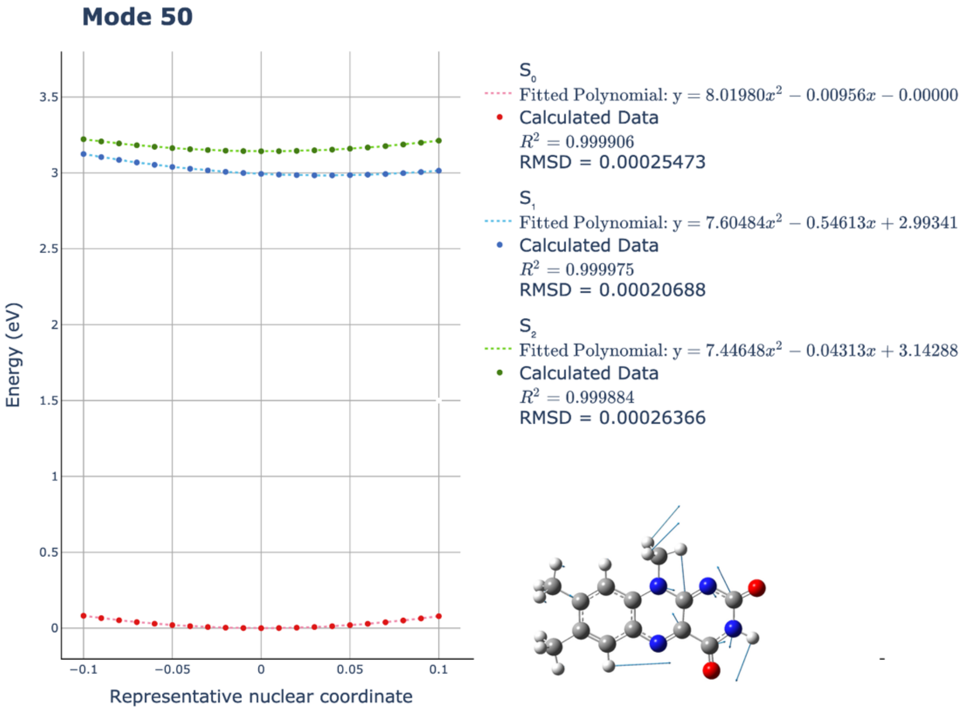

Fig. S5 (continued)

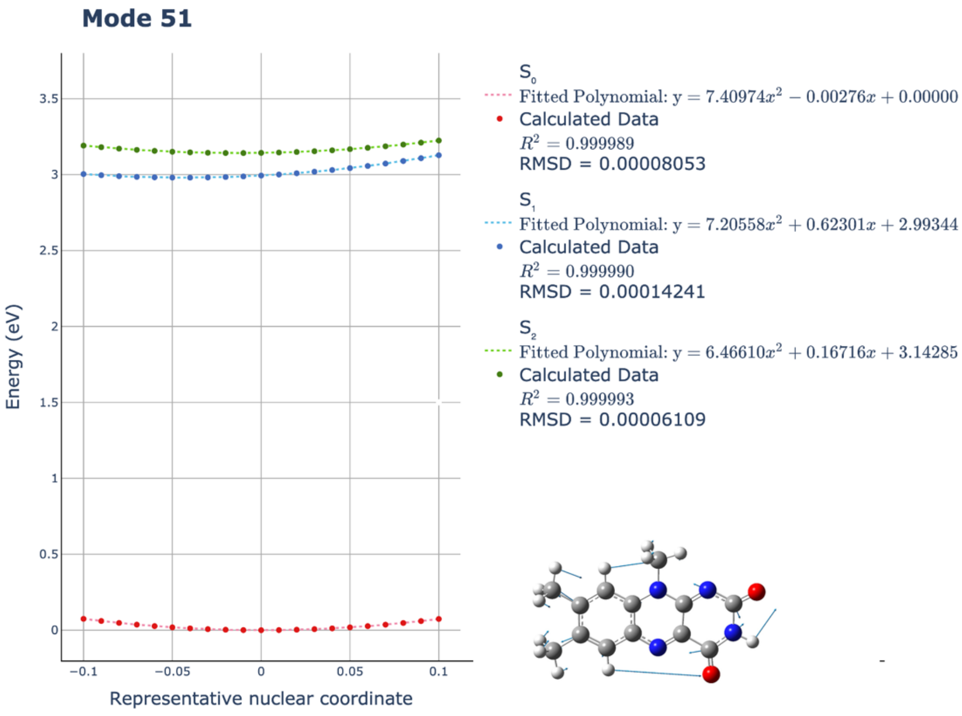

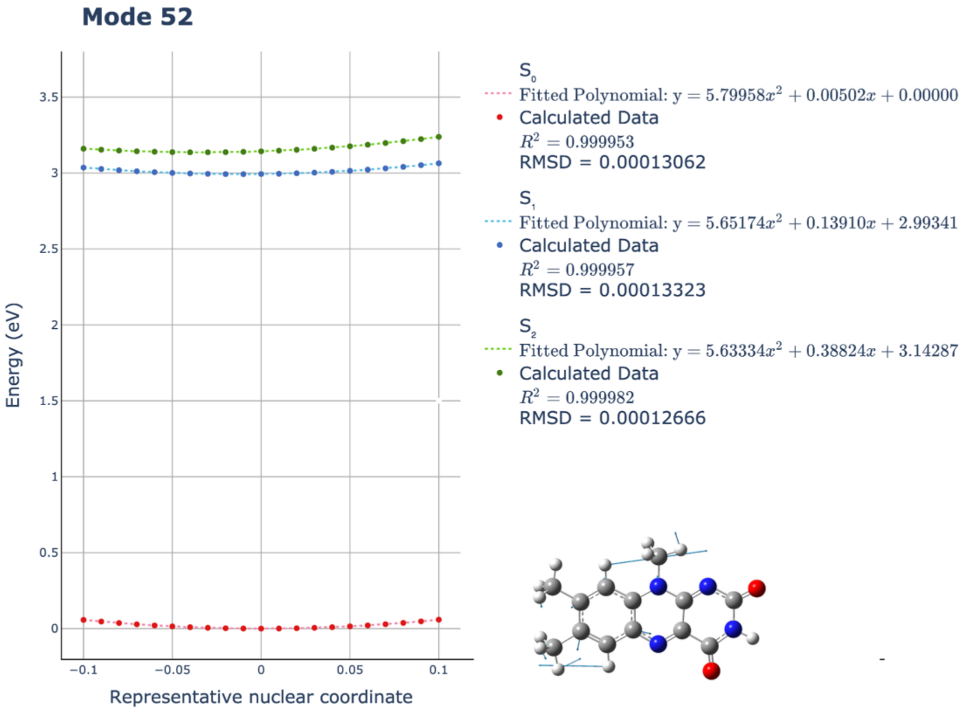

Fig. S5 (continued)

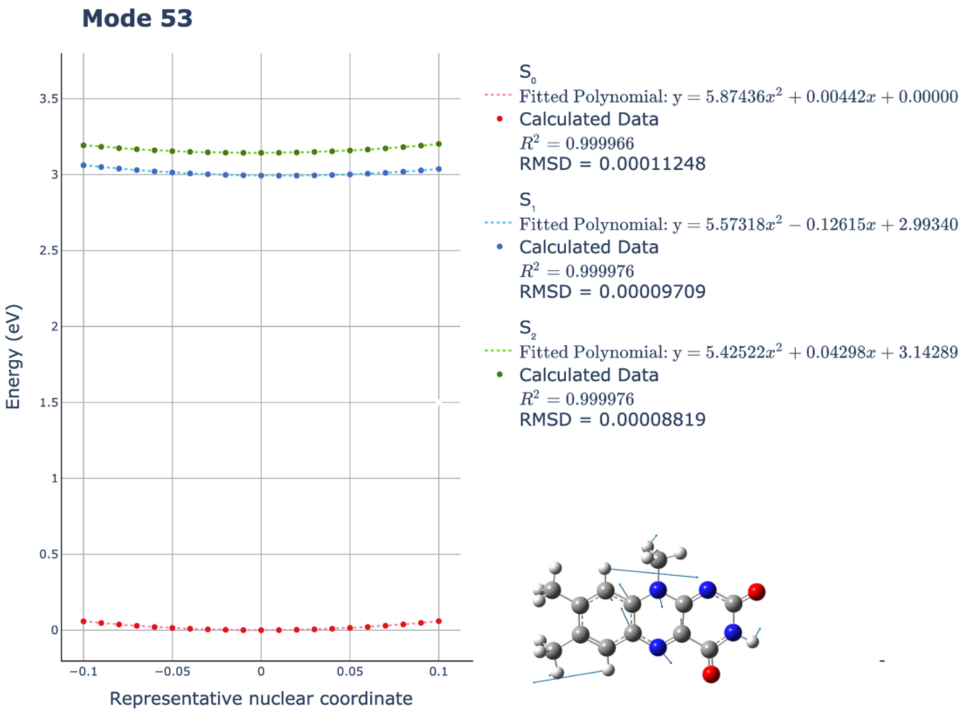

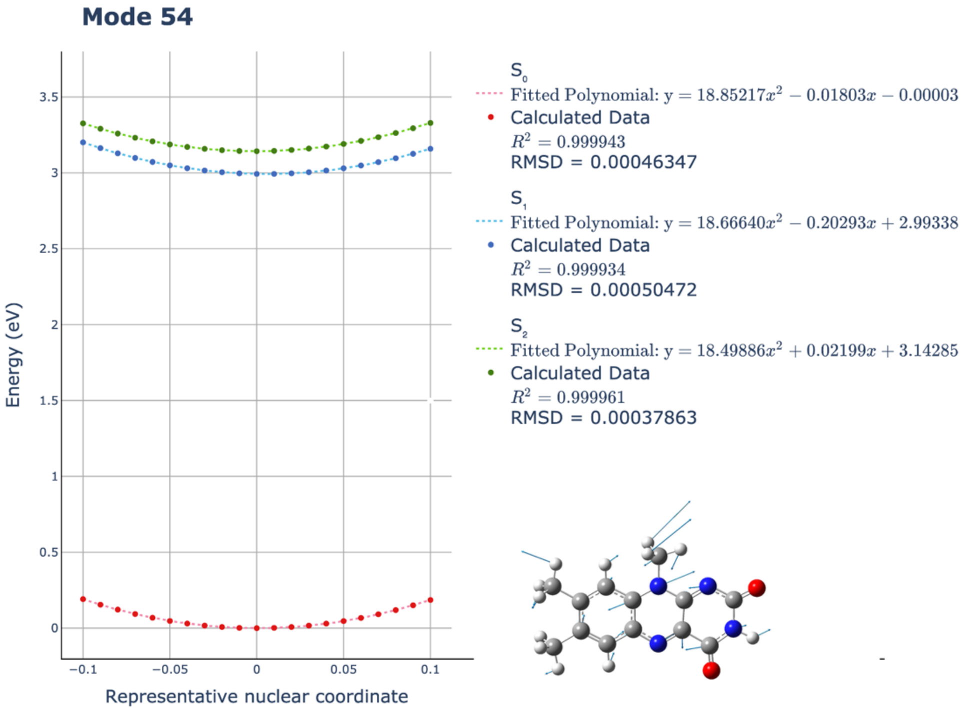

Fig. S5 (continued)

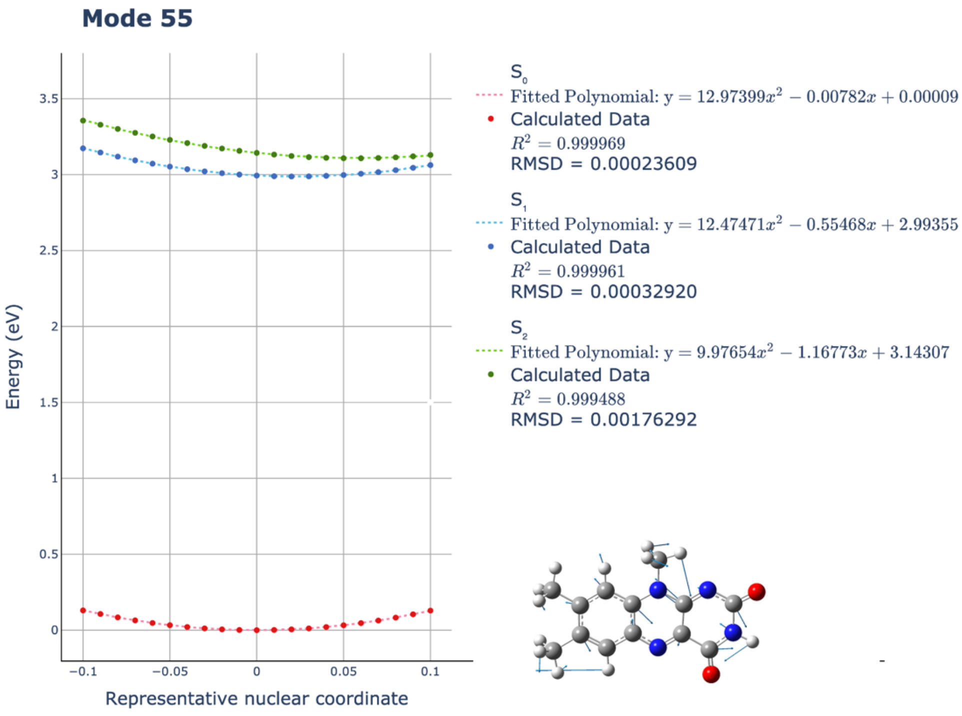

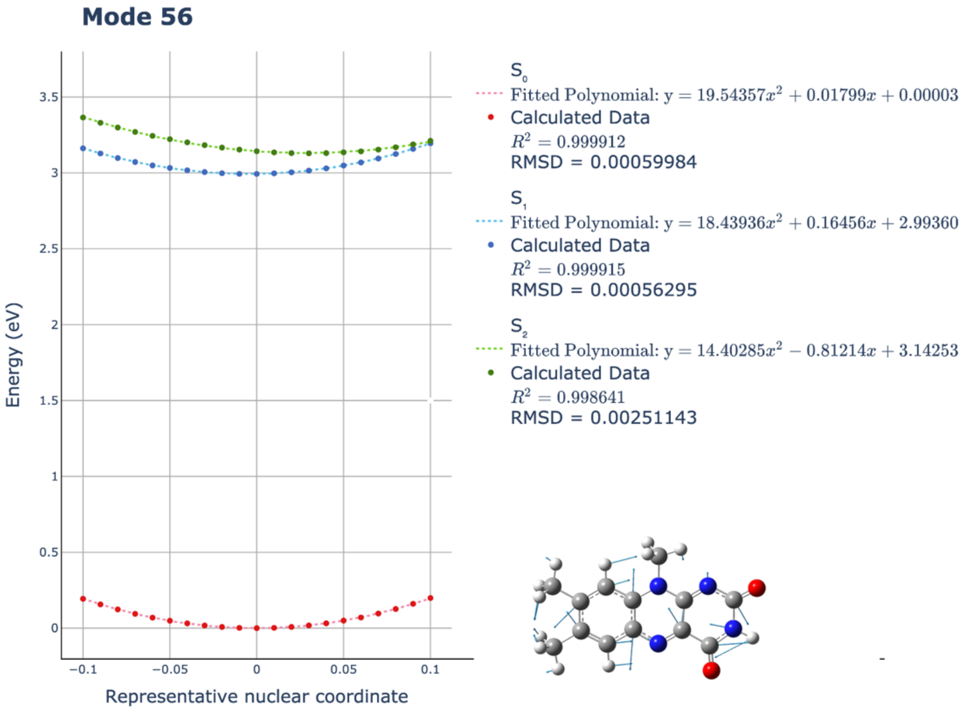

Fig. S5 (continued)

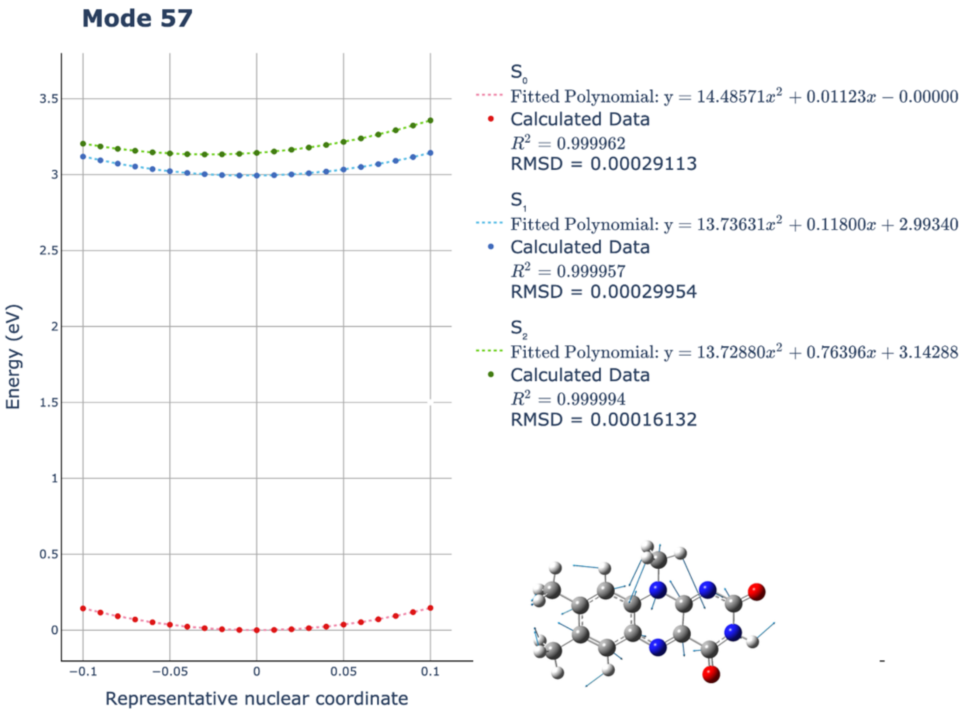

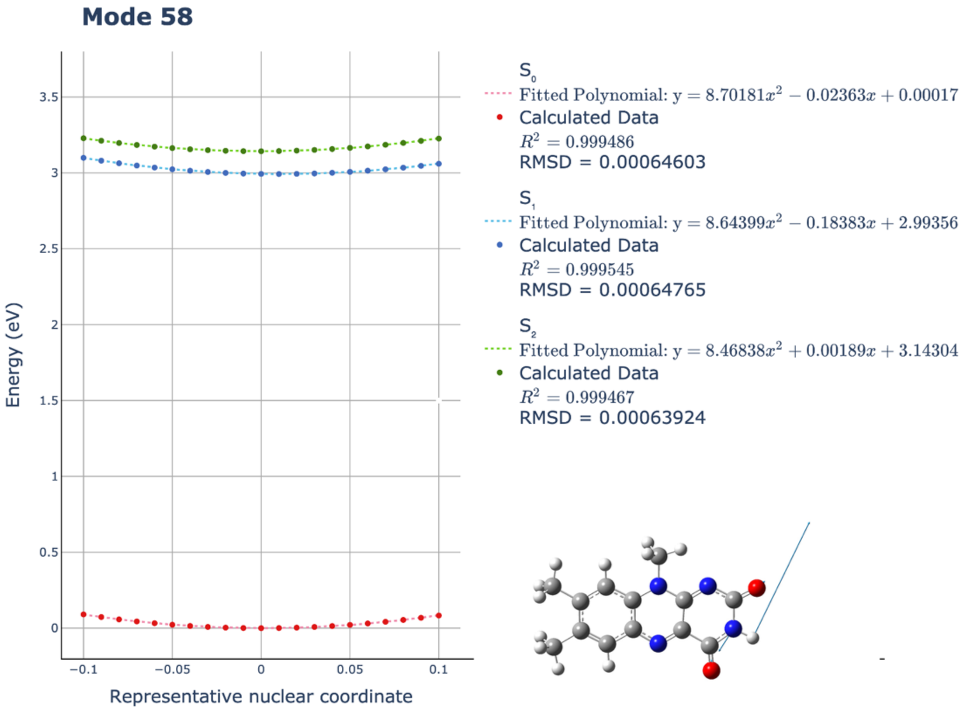

Fig. S5 (continued)

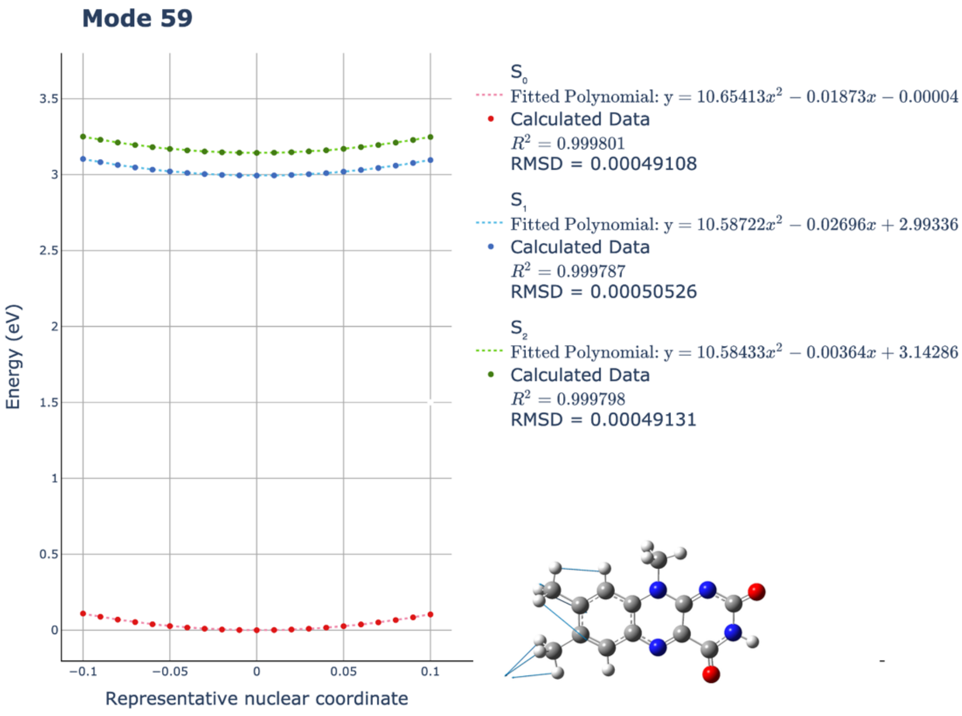

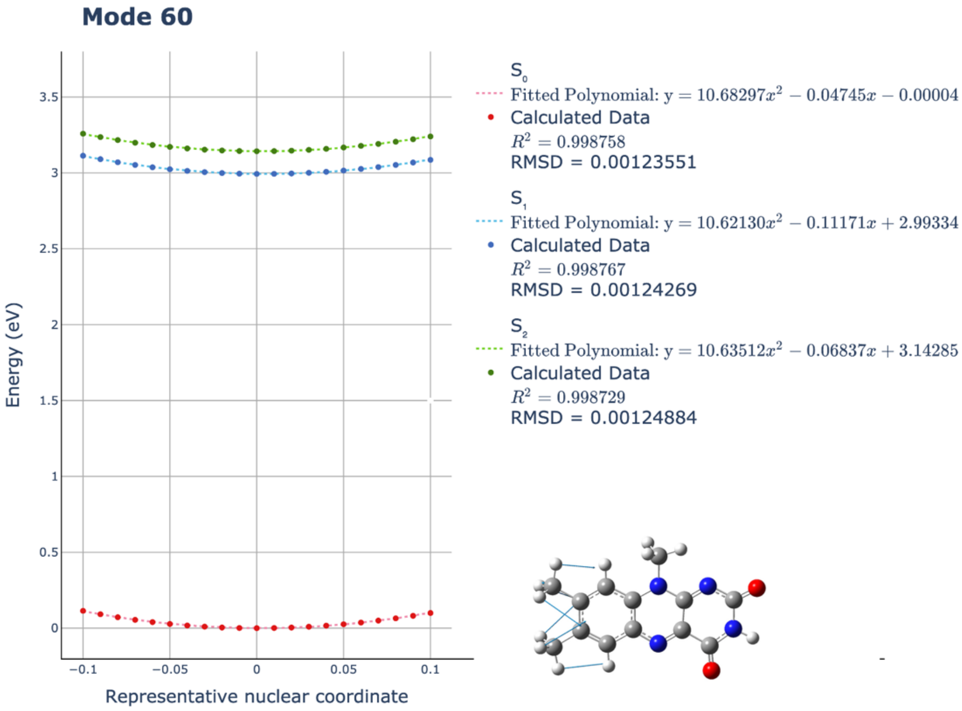

Fig. S5 (continued)

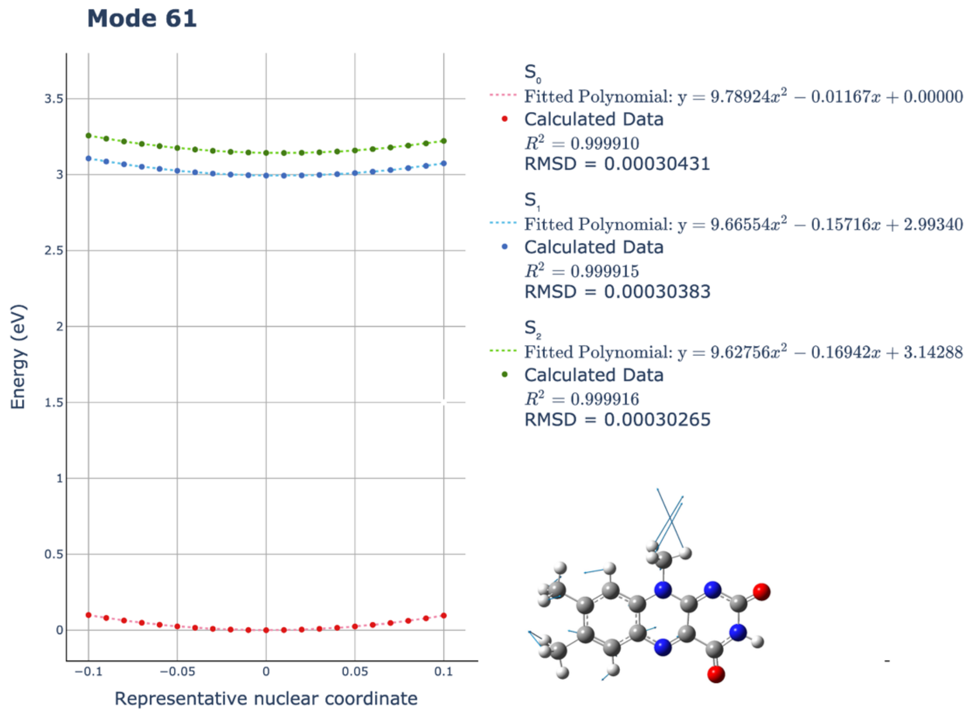

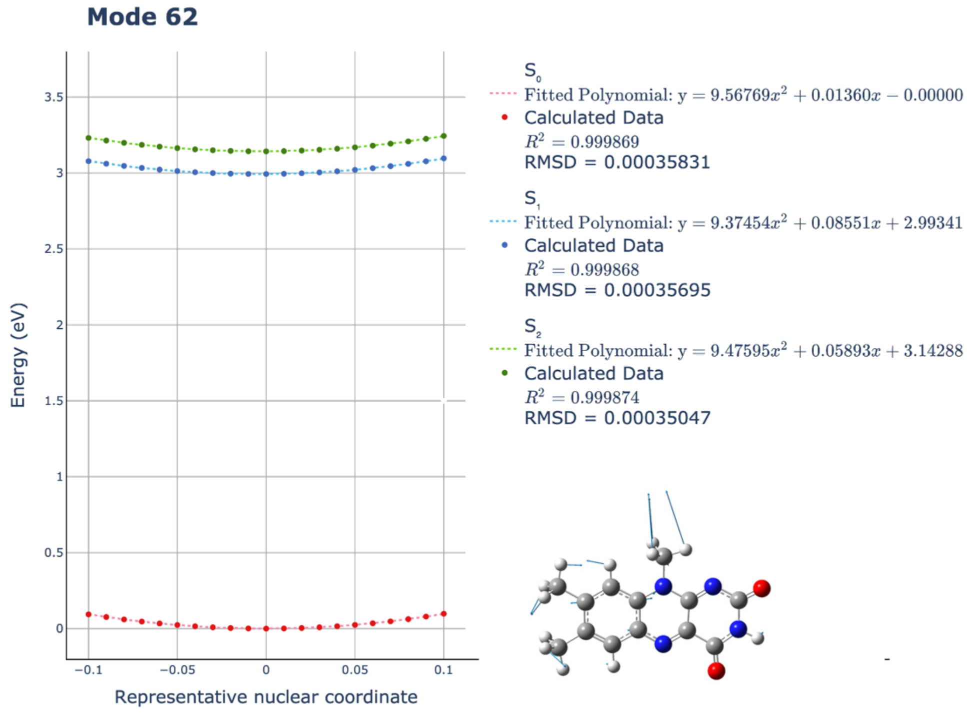

Fig. S5 (continued)

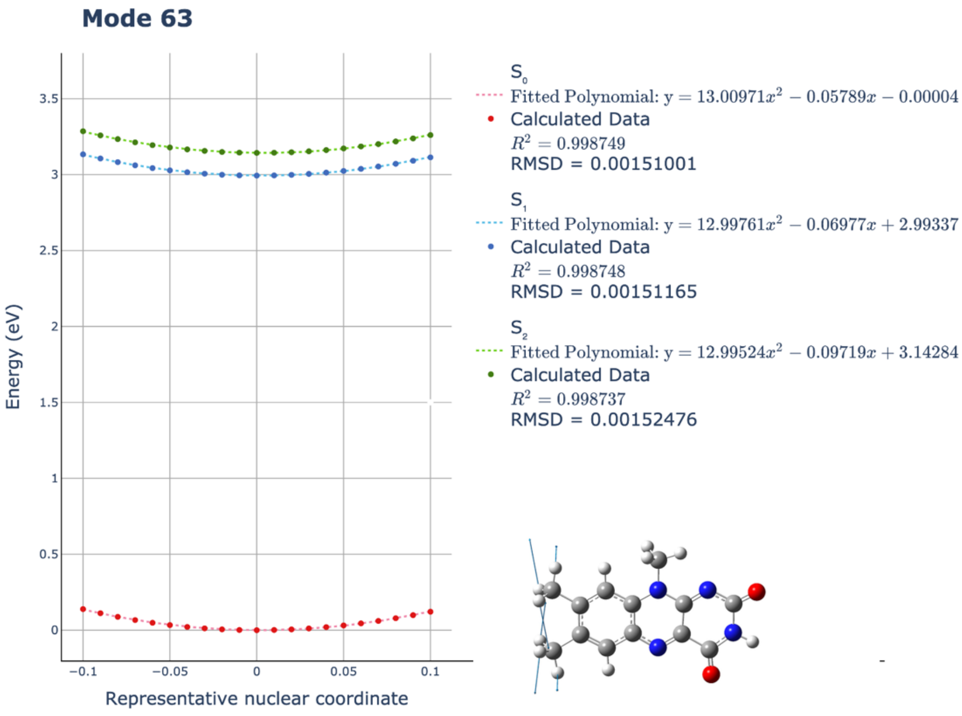

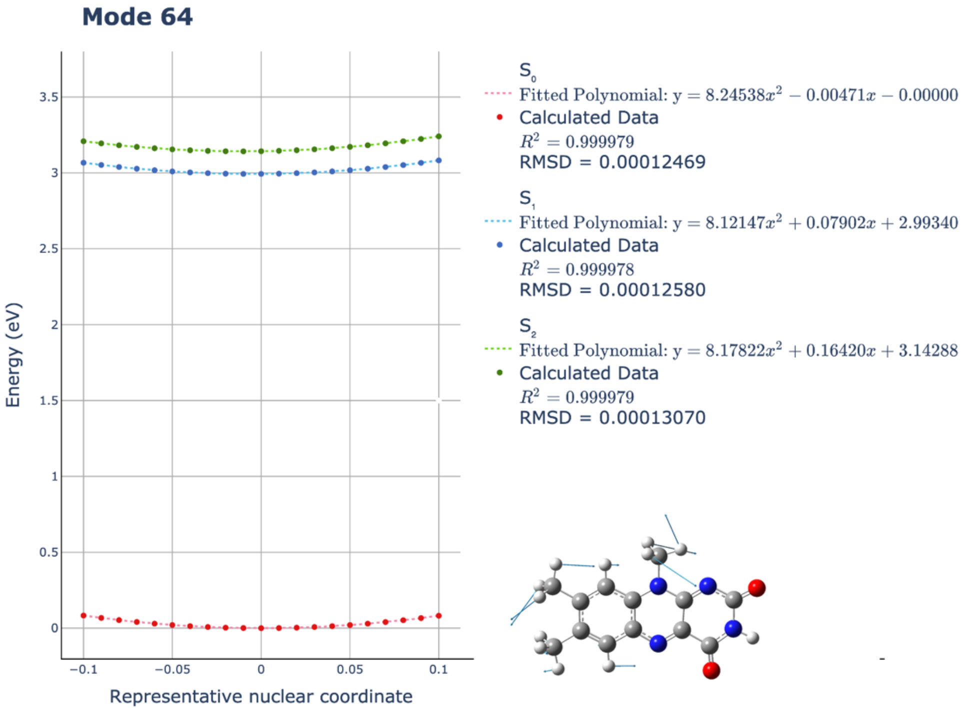

Fig. S5 (continued)

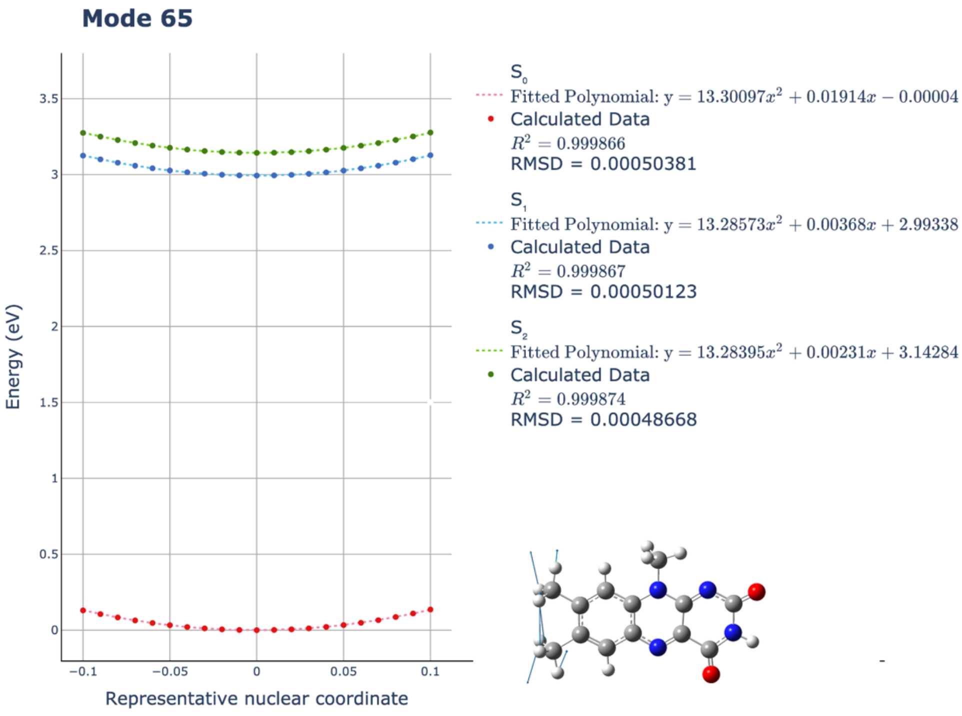

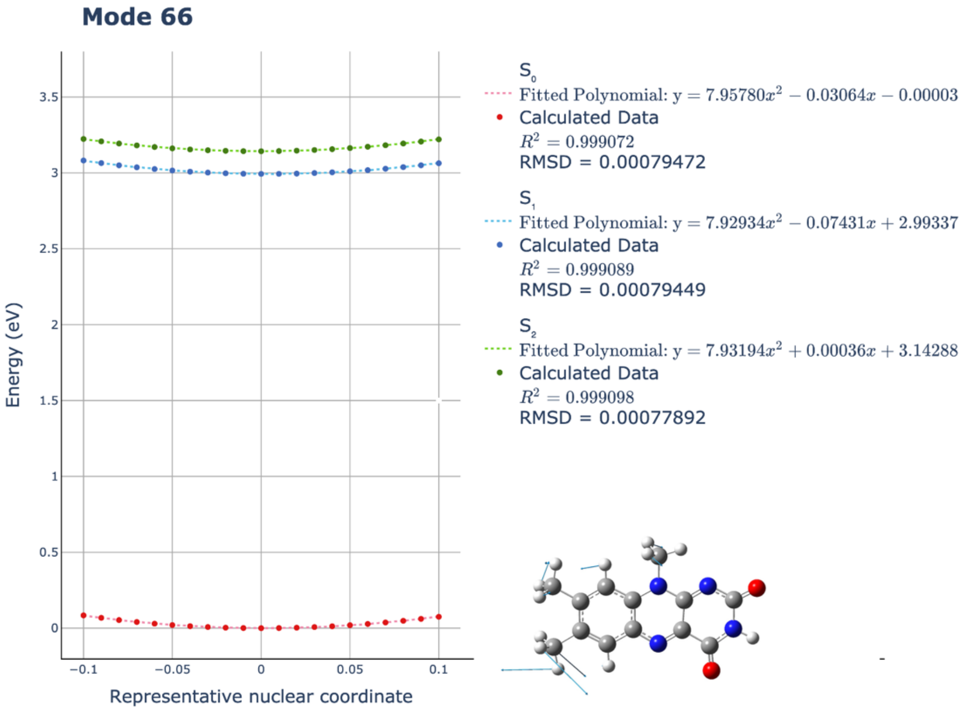

Fig. S5 (continued)

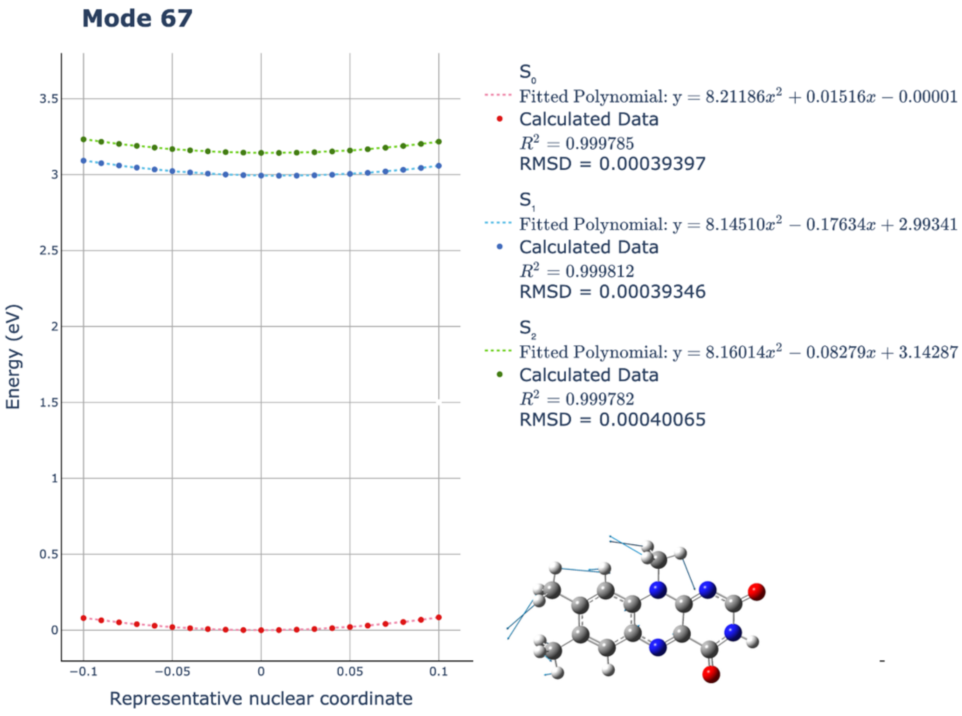

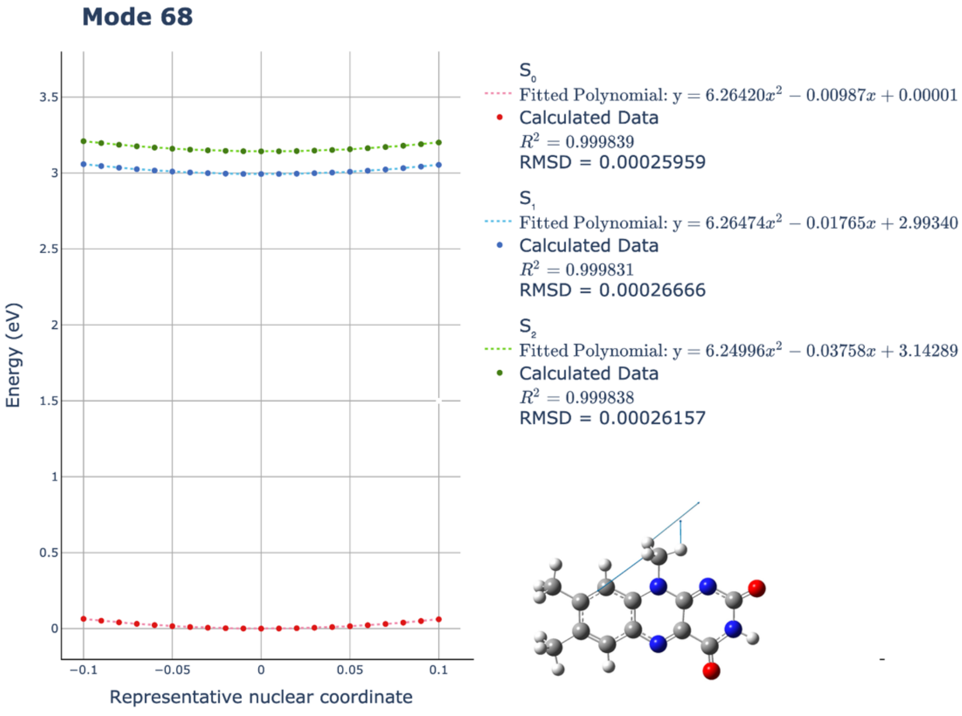

Fig. S5 (continued)

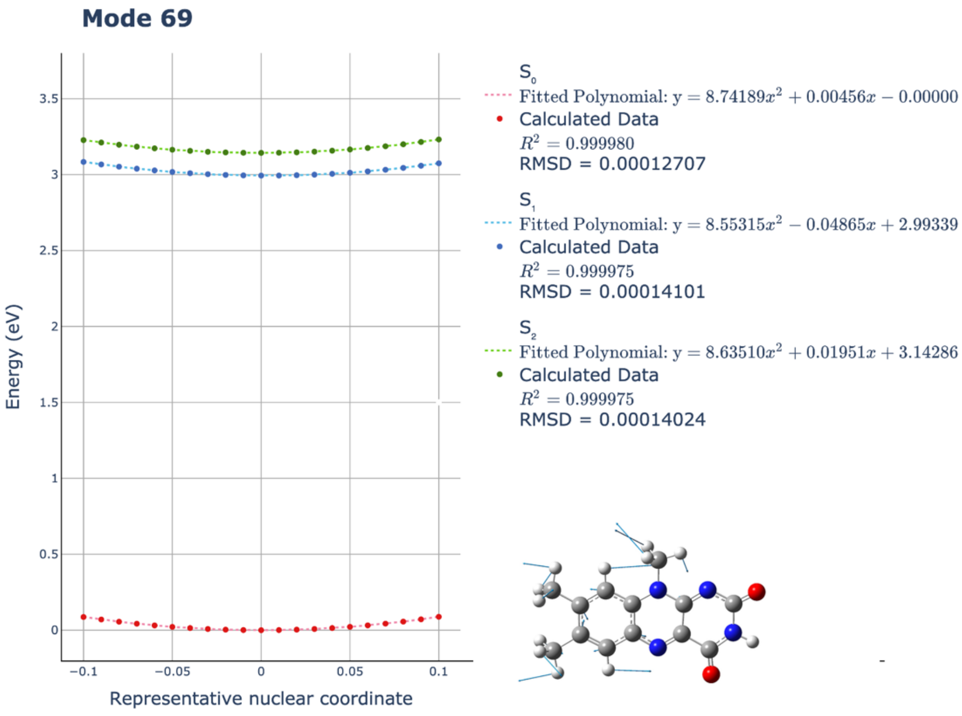

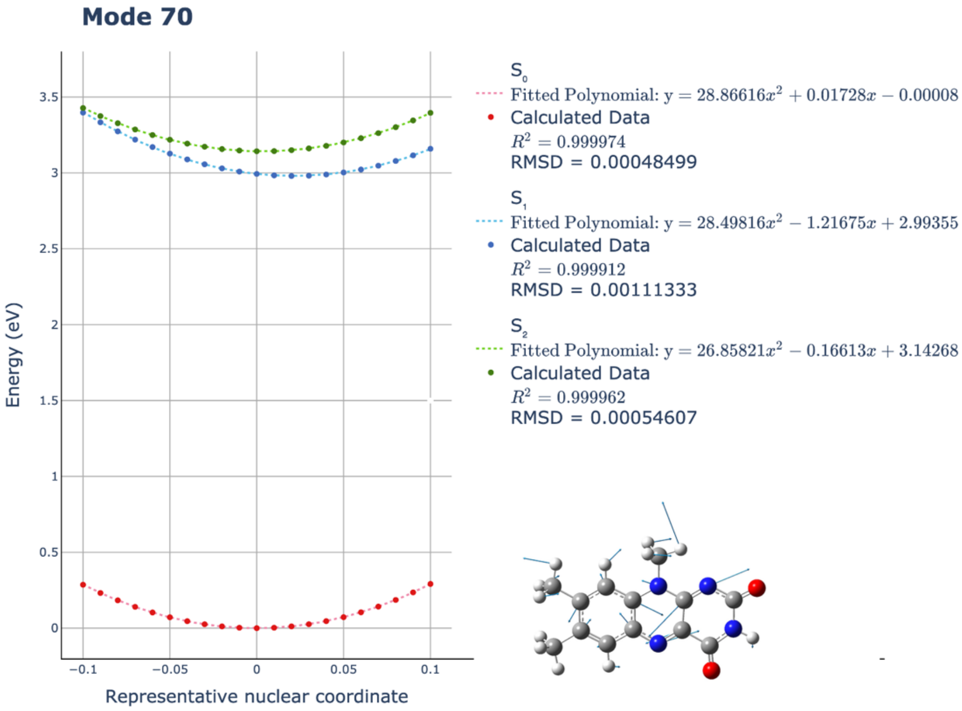

Fig. S5 (continued)

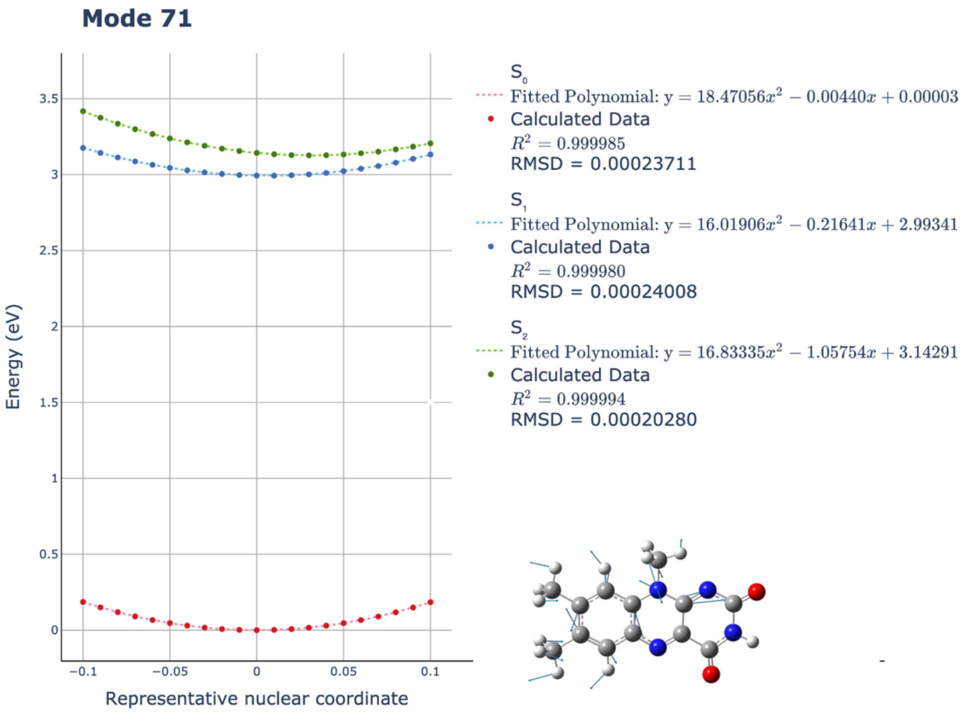

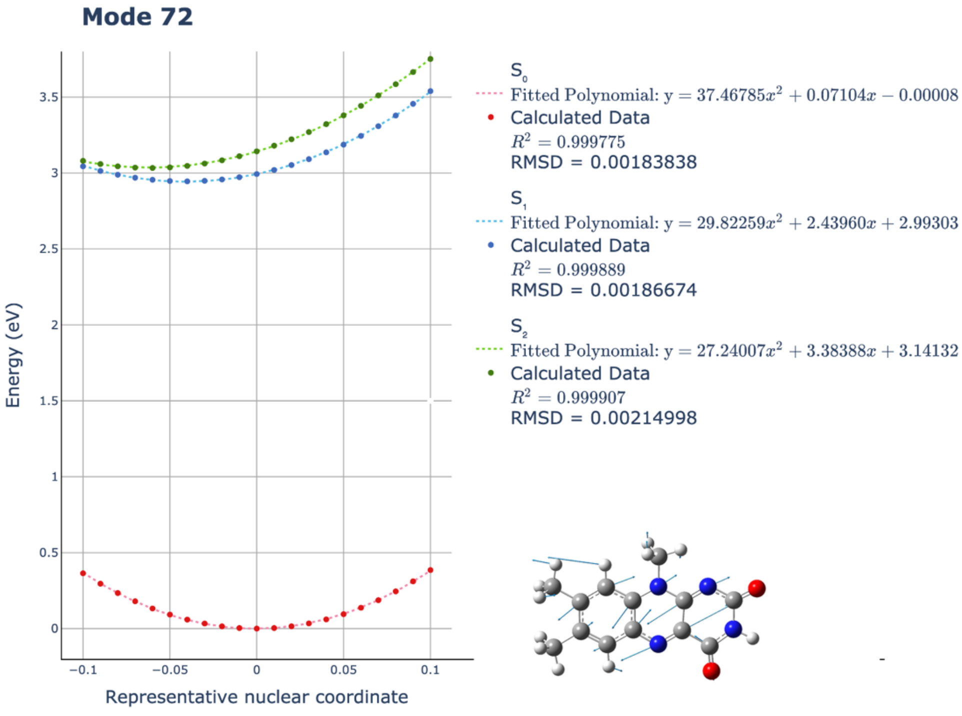

Fig. S5 (continued)

Mode 73

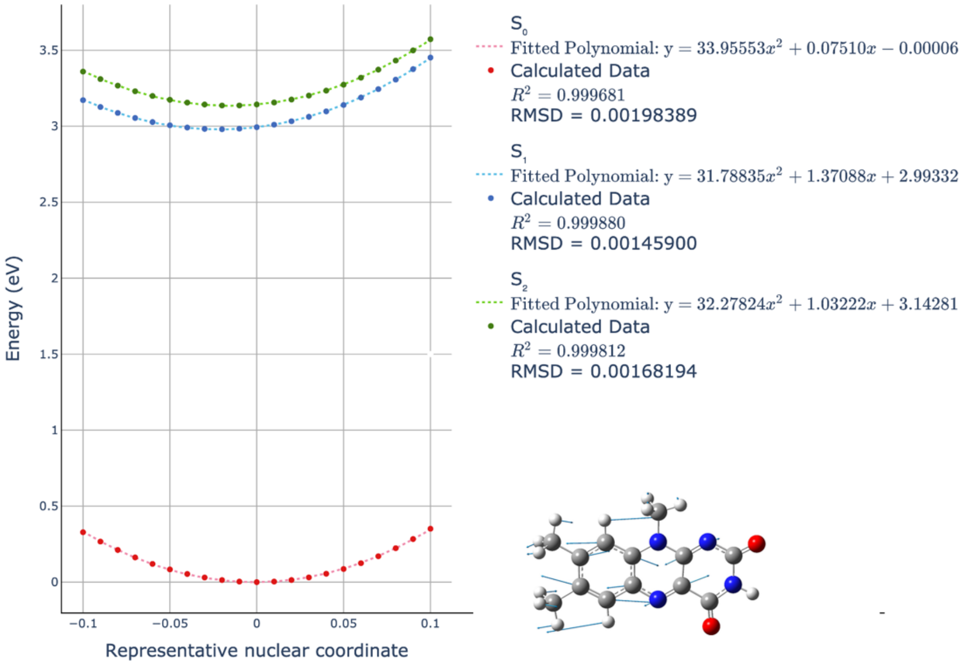

Mode 74

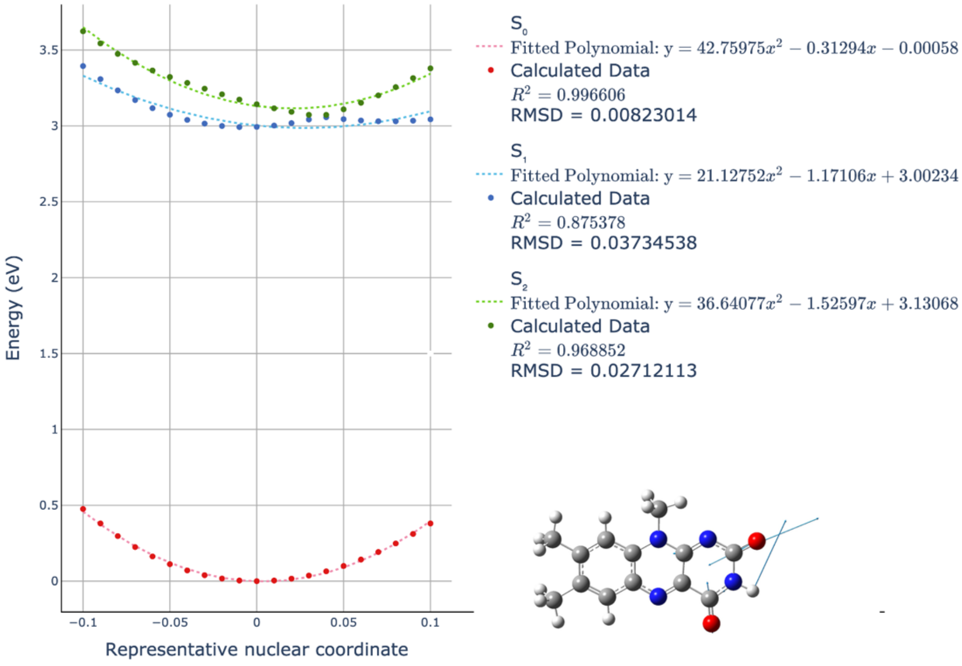

Fig. S5 (continued)

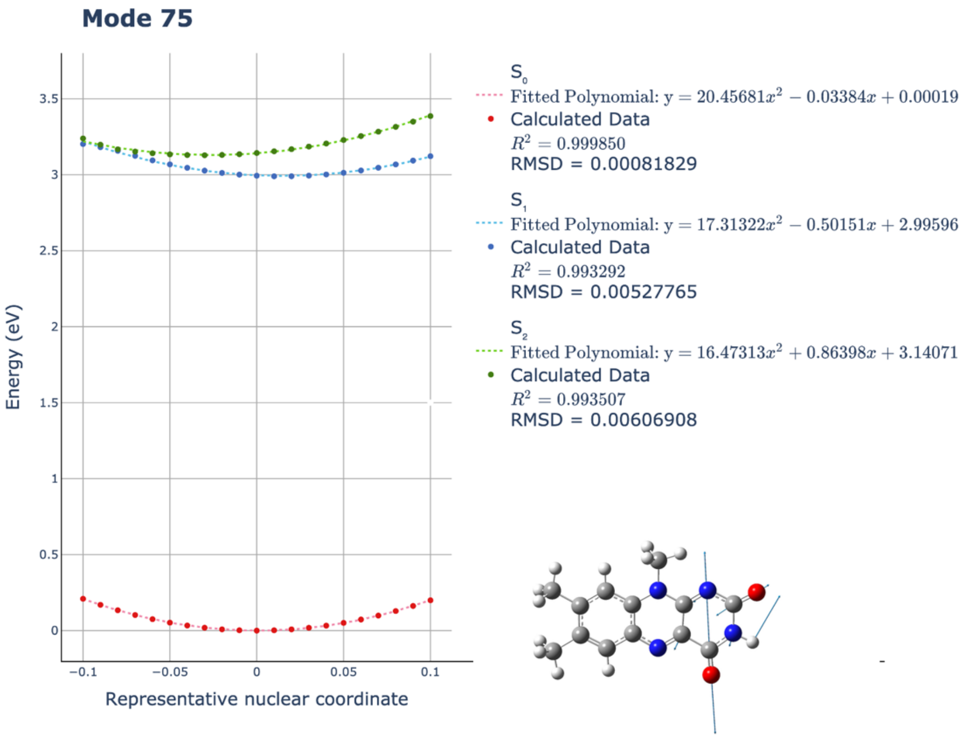

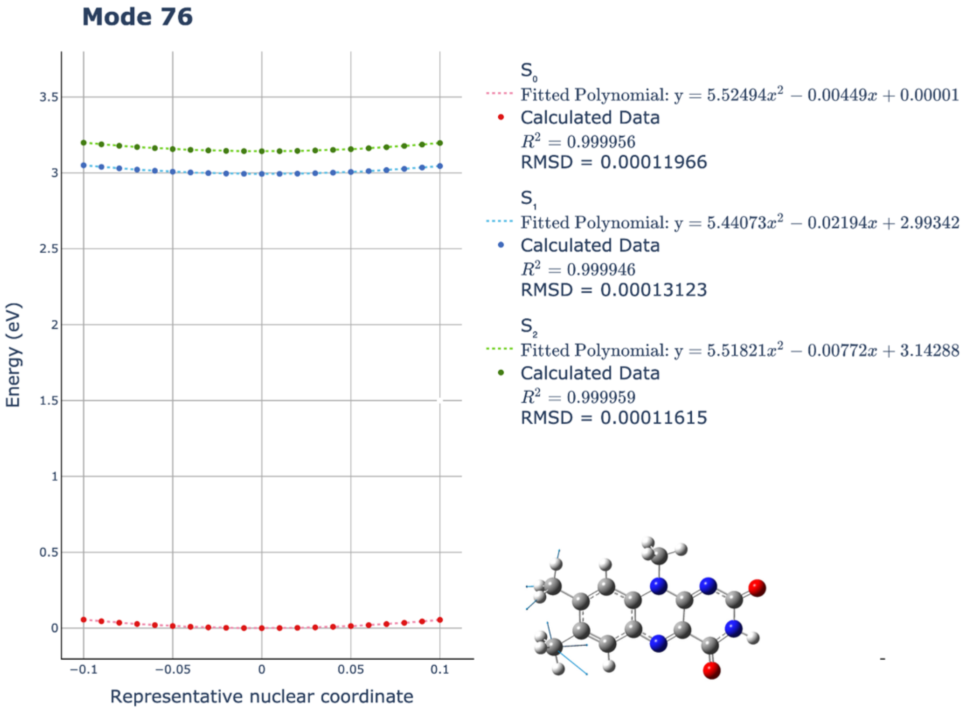

Fig. S5 (continued)

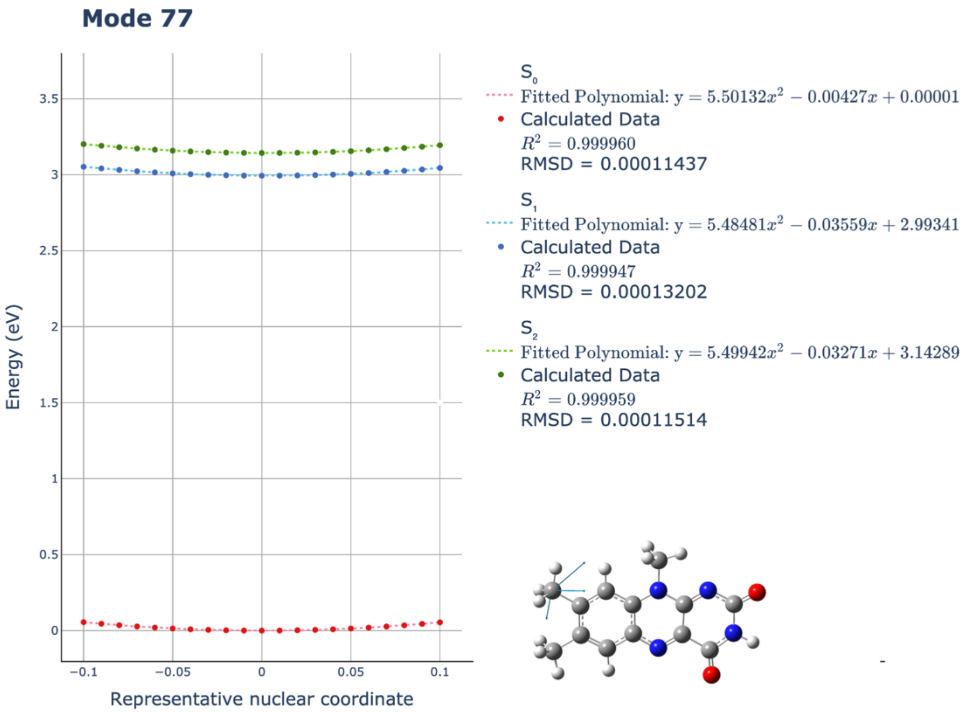

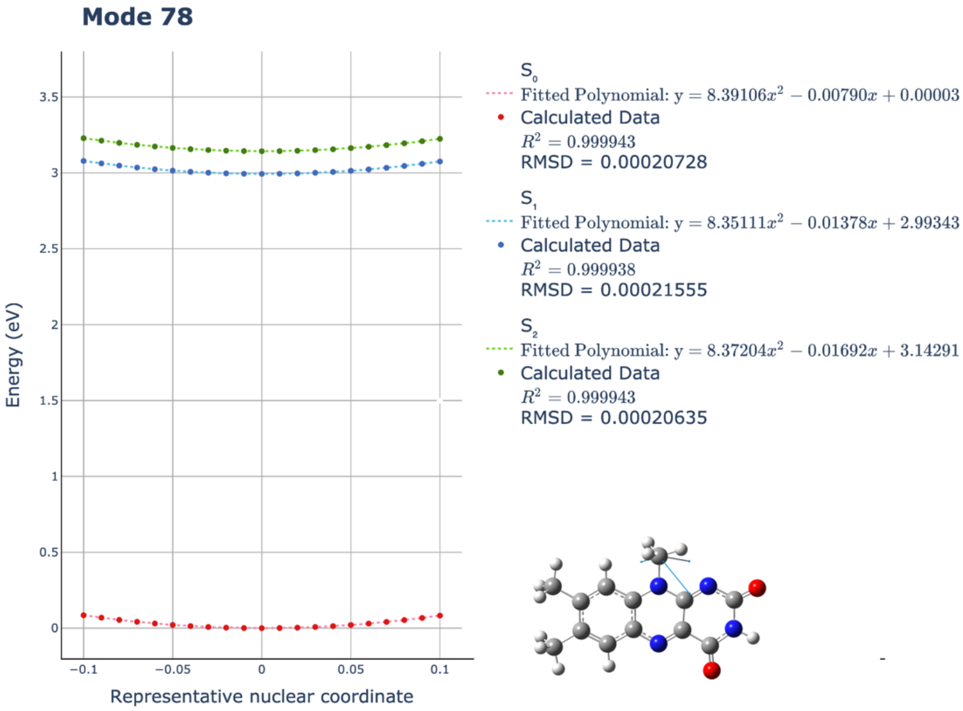

Fig. S5 (continued)

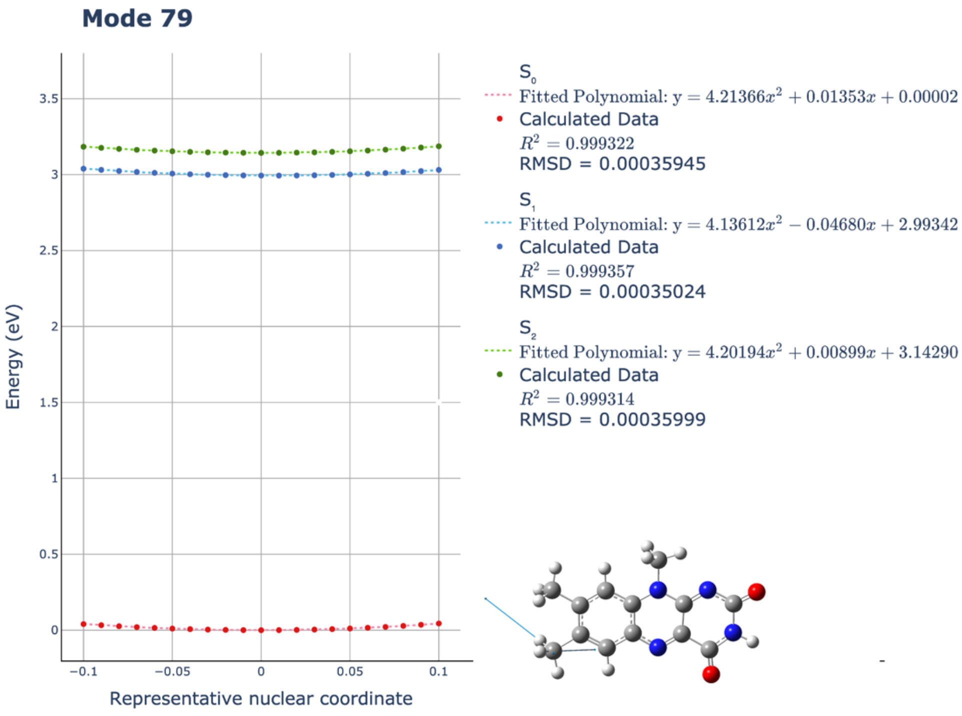

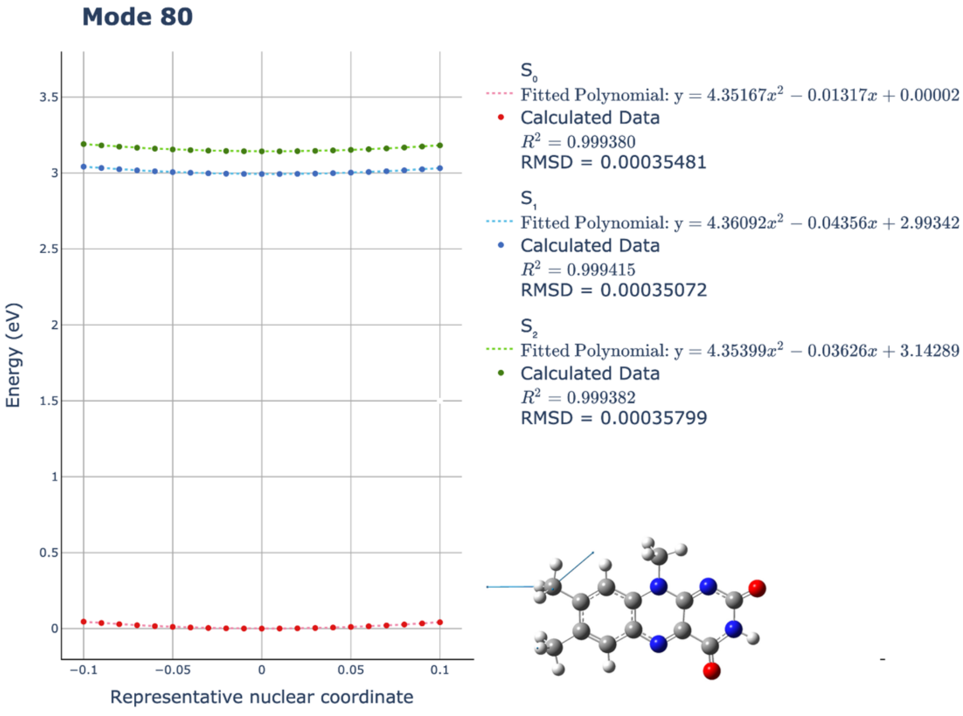

Fig. S5 (continued)

Mode 81

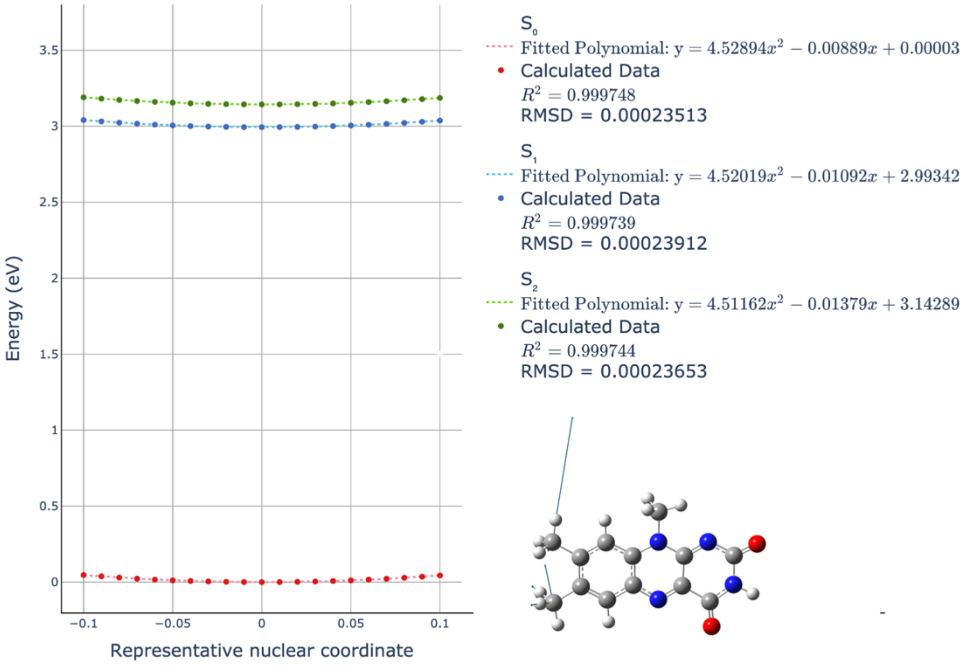

Mode 82

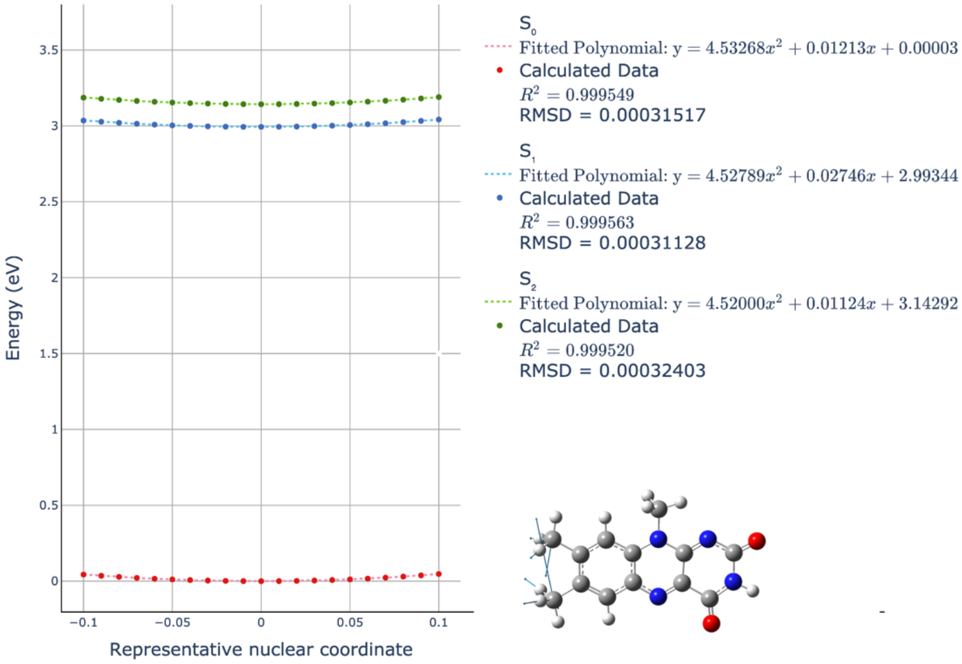

Fig. S5 (continued)

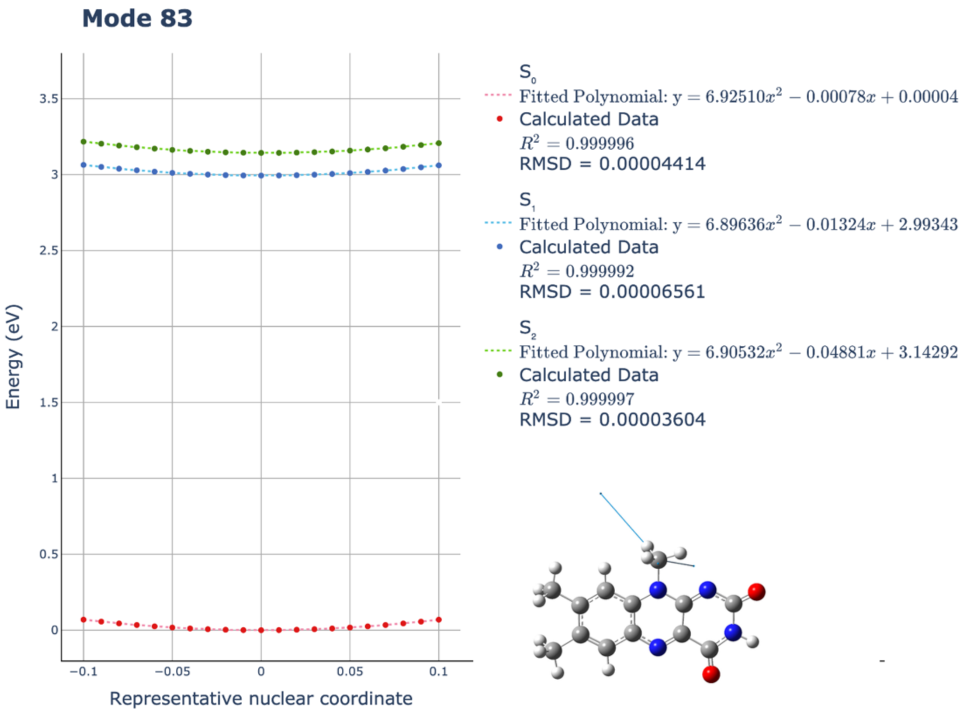

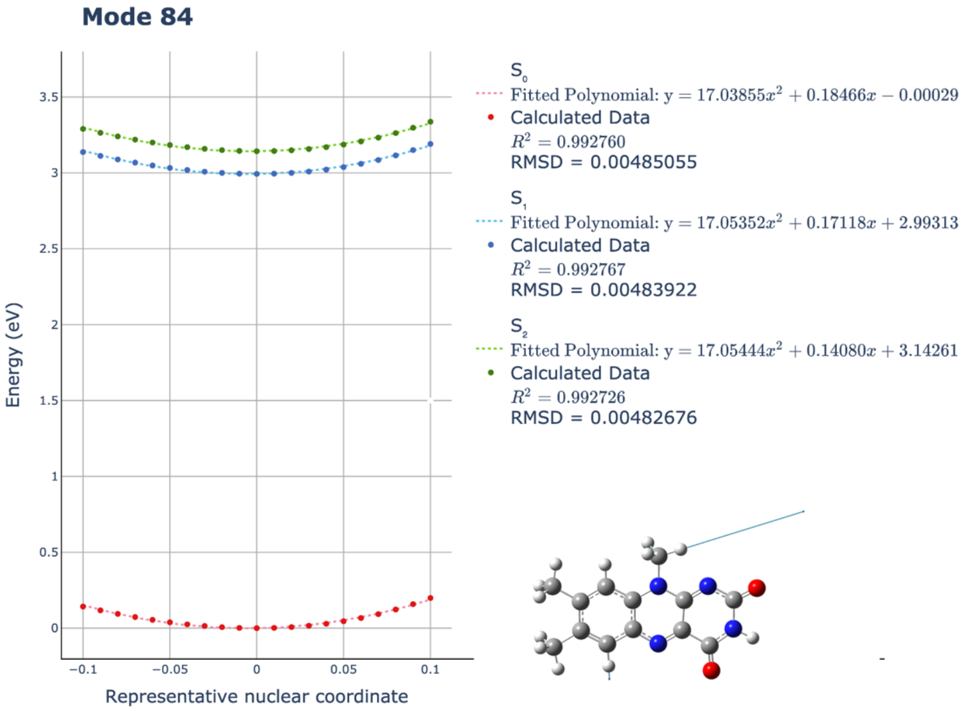

Fig. S5 (continued)

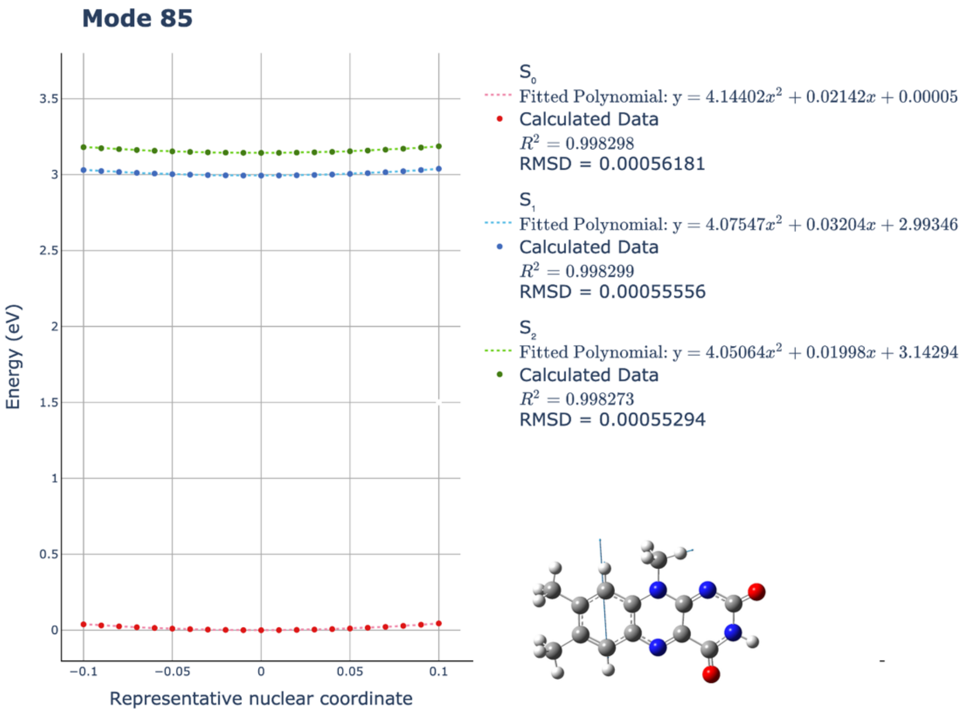

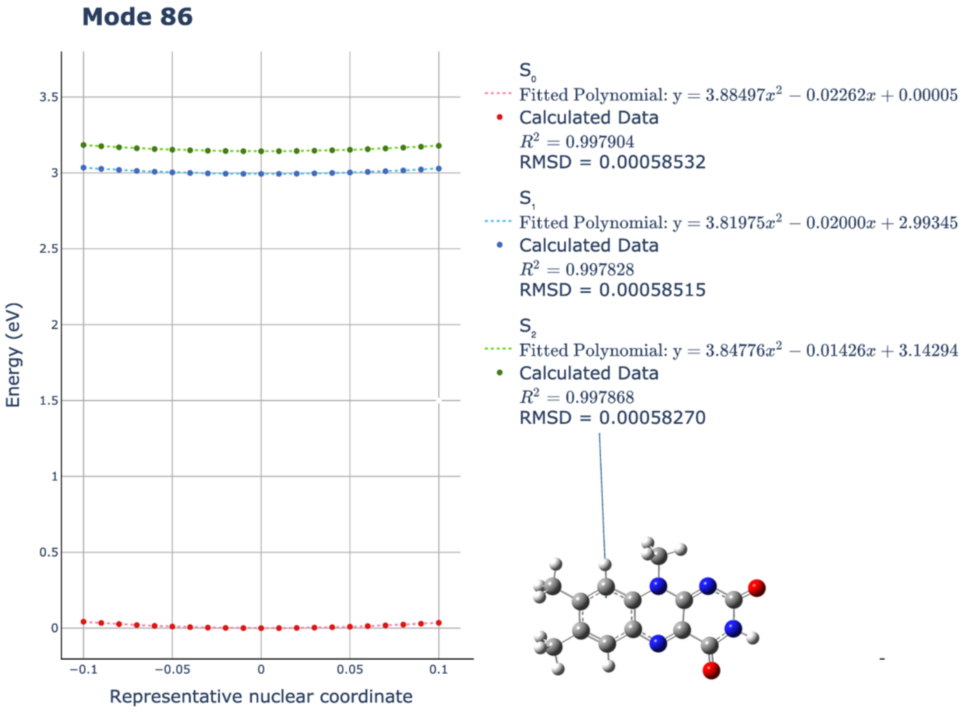

Fig. S5 (continued)

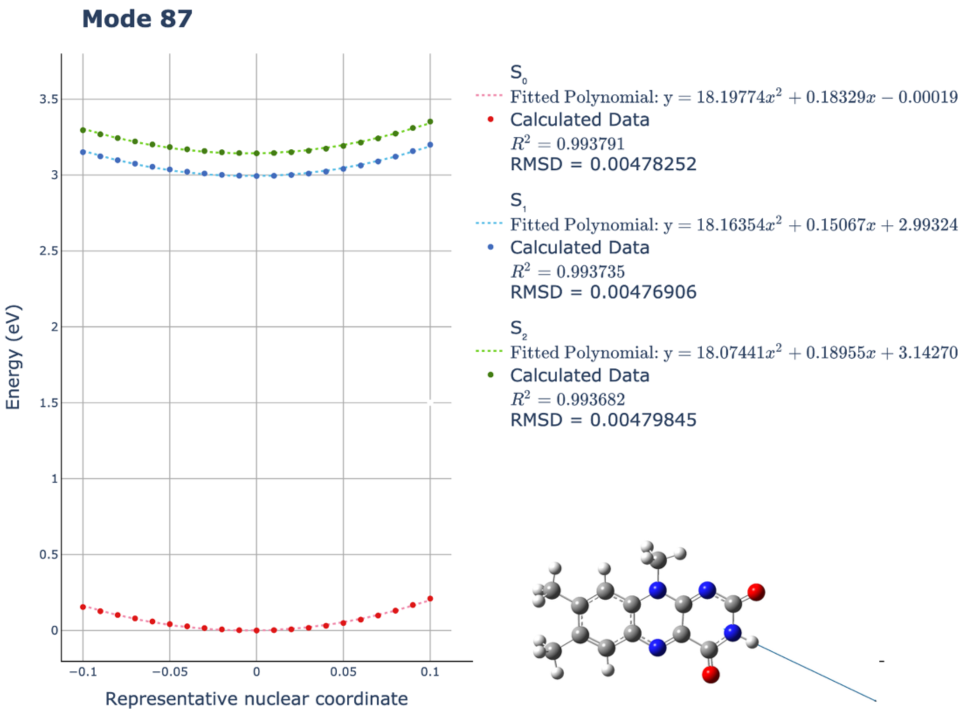

Fig. S5 (continued)

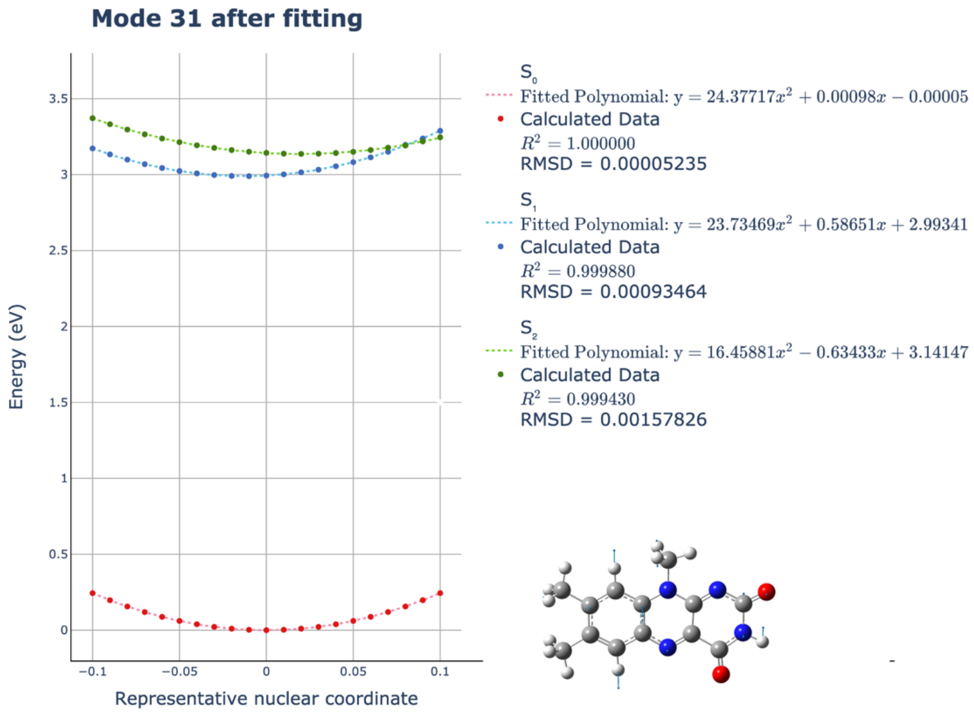

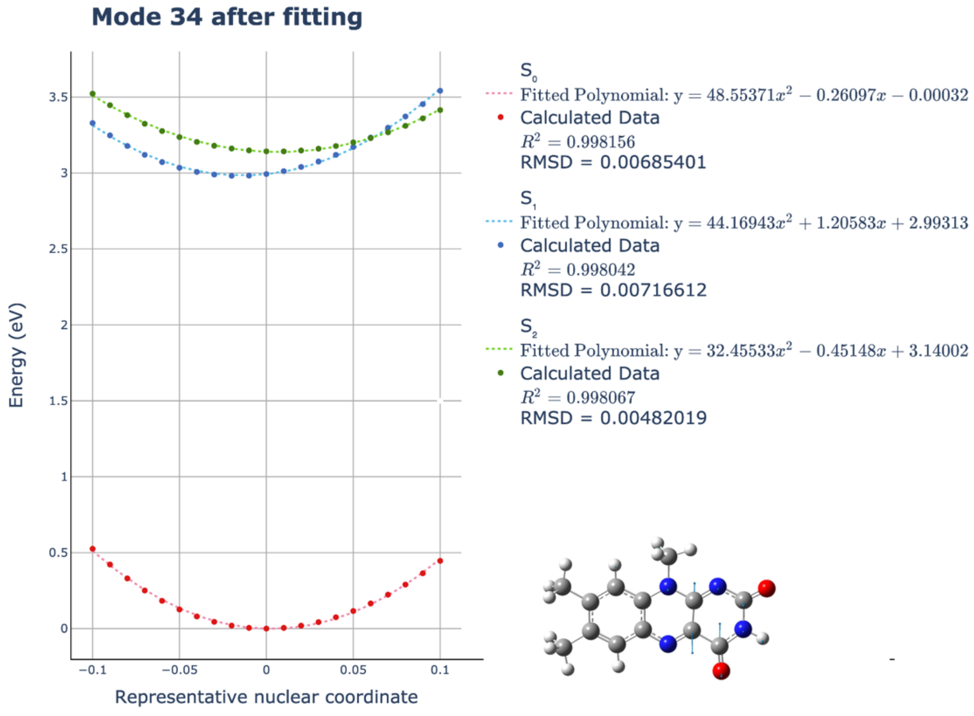

Fig. S6 *Lumiflavin potential energy scans (scaling factor -0.1 ≤ i ≤ 0.1) at ground state (S_0), first excited state (S_1), and second excited states (S_2) with vibrational motion, quadratic equation, R^2, and RMSD values for mode 31, 34, 74, and 75 after fitting at CI point.* *(continued on next page)*

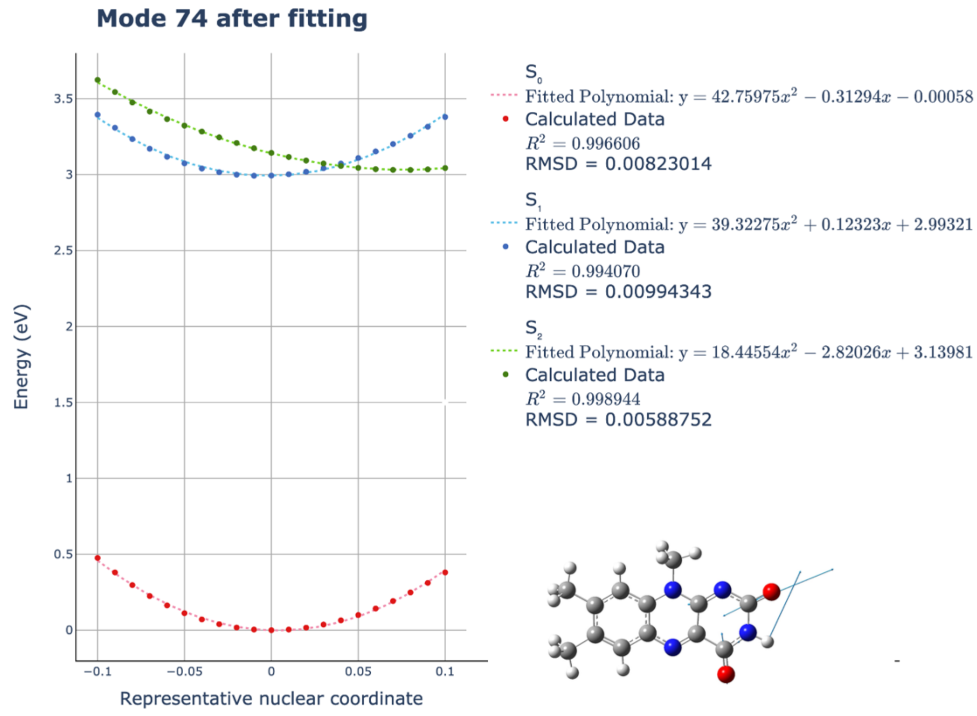

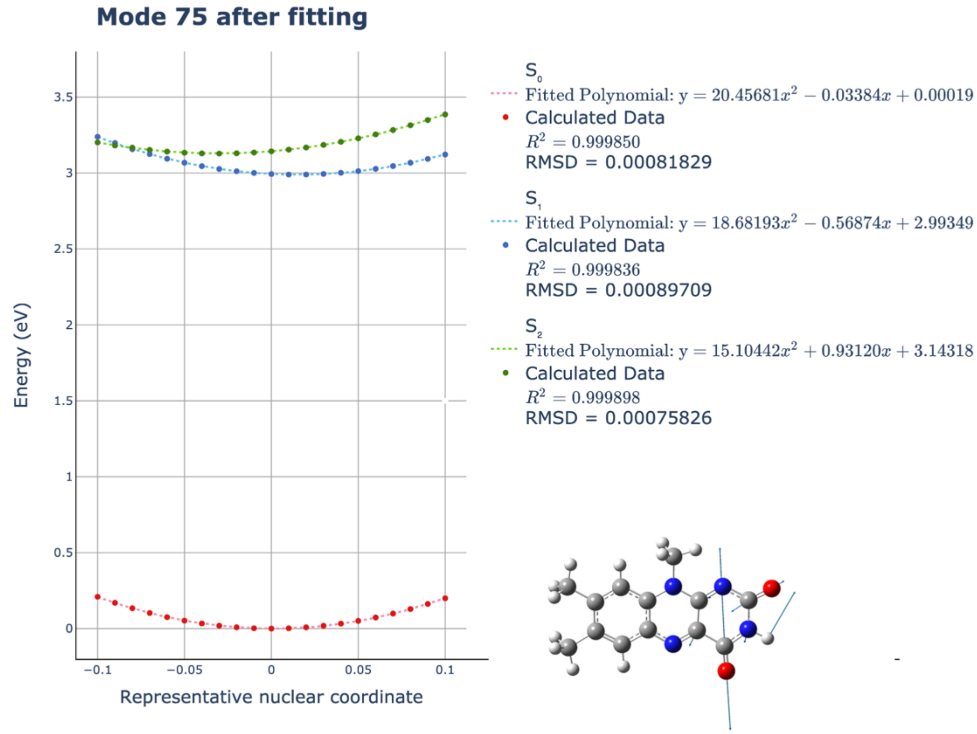

Fig. S6 *(continued)*

Table S1 *Vibrational frequencies, infrared (IR) intensities, and molar extinction coefficient (ε) of lumiflavin computed at the B3LYP/6-31+G* level of theory. In each row, the last column displays the same full simulated IR spectrum with a small broadening factor, but with a small red triangle that shifts in each row to indicate the position of the corresponding mode.*

**Mode**	**Frequency (cm^{-1})**	**IR intensity (km/mole)**	**ε (M^{-1} cm^{-1})**	**Corresponding IR band (red highlighted)**
1	40.9305	0.6829	66.5606	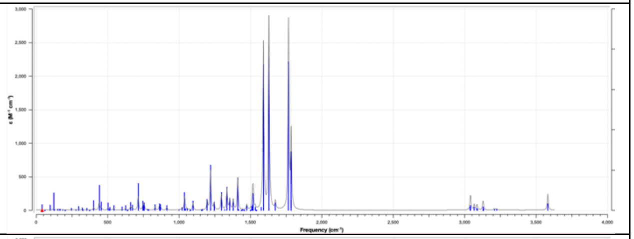
2	60.9545	0.0201	1.3155	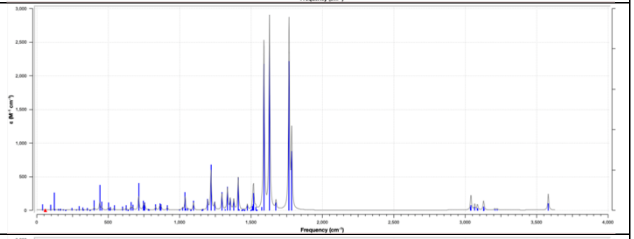
3	97.8601	1.5210	62.0056	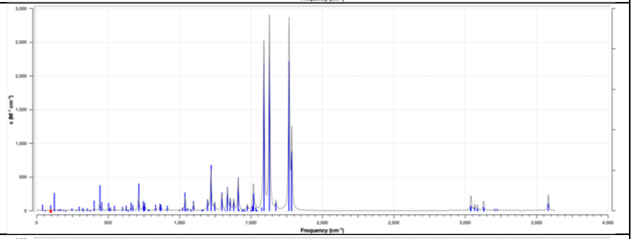
4	123.1494	6.6404	215.1143	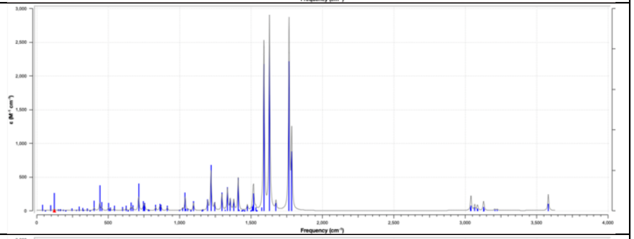
5	127.5000	0.2838	8.8799	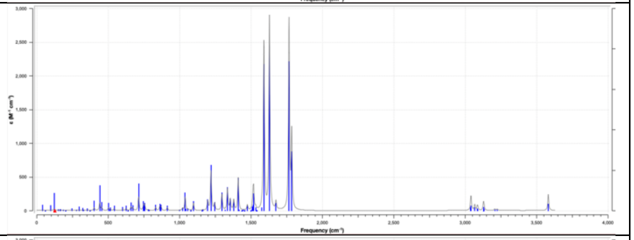
6	150.6148	0.4112	10.8916	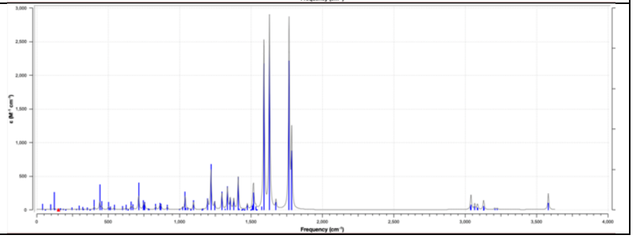

7	164.1921	0.4450	10.8122	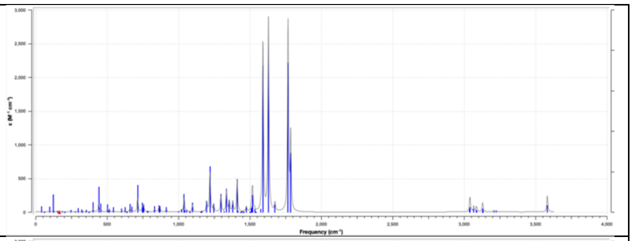
8	167.1454	0.5534	13.2085	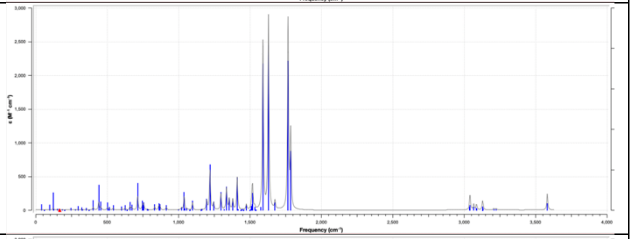
9	185.2688	0.3053	6.5740	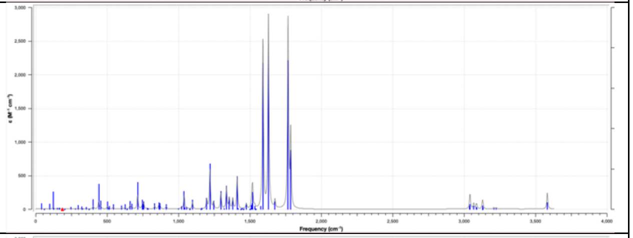
10	201.6627	0.0145	0.2868	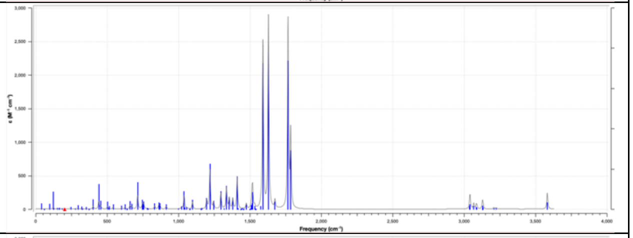
11	245.9944	1.5083	24.4608	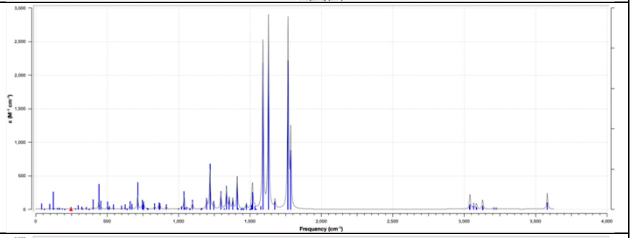
12	279.2596	0.4599	6.5700	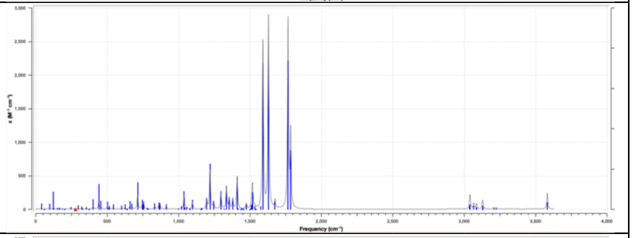
13	296.6244	3.2706	43.9874	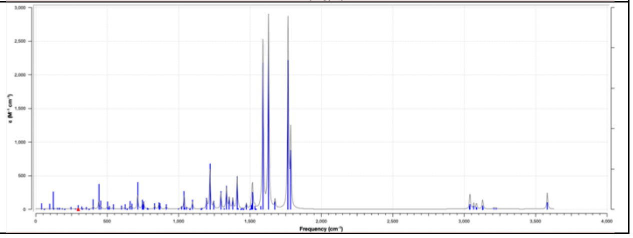

14	323.5108	2.2854	28.1826	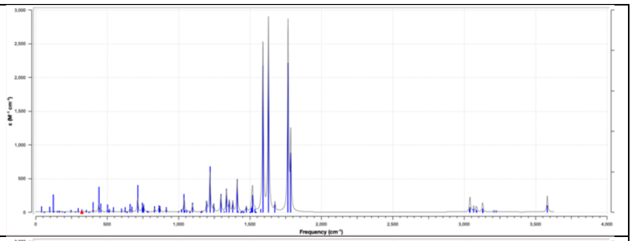
15	328.1495	0.3187	3.8745	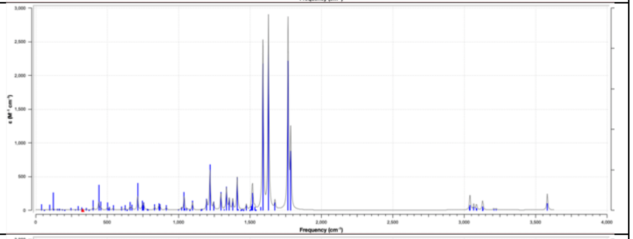
16	355.4505	2.1885	24.5626	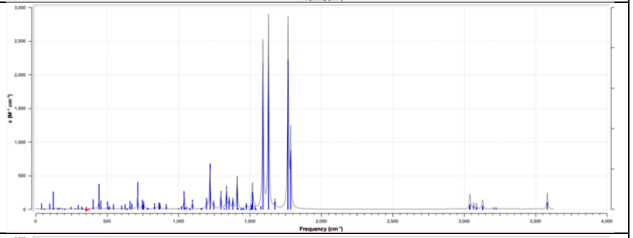
17	374.1469	0.0001	0.0011	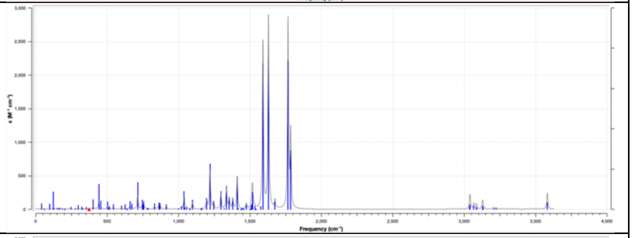
18	402.4906	12.1095	120.0267	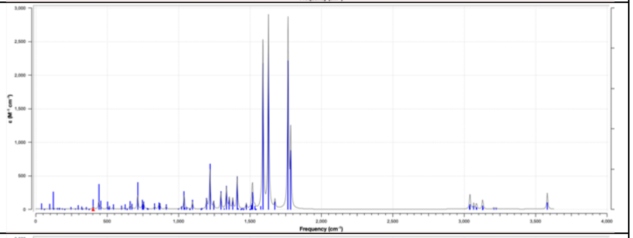
19	441.5384	34.0140	307.3240	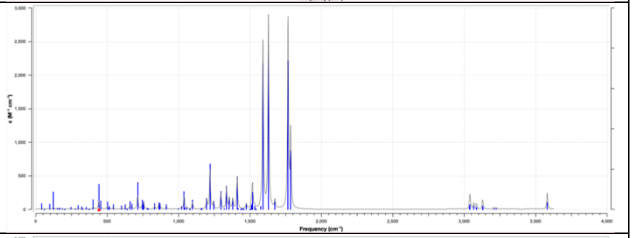
20	454.9173	11.6580	102.2348	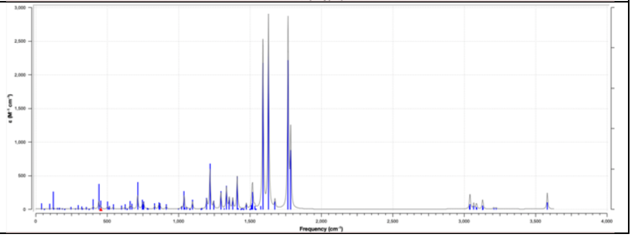

21	502.1481	11.1785	88.8094	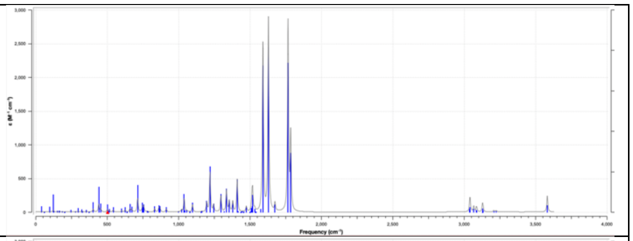
22	514.0203	2.0038	15.5518	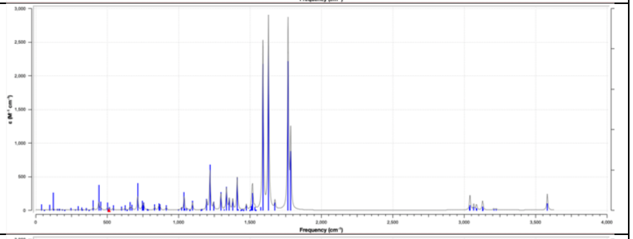
23	515.7642	4.1190	31.8602	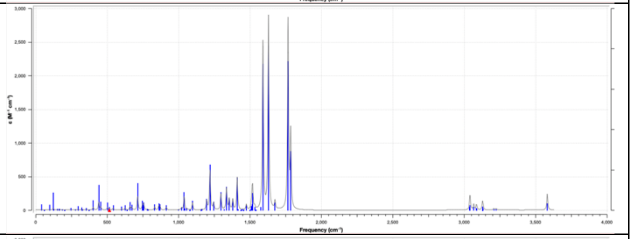
24	543.1326	7.6097	55.8945	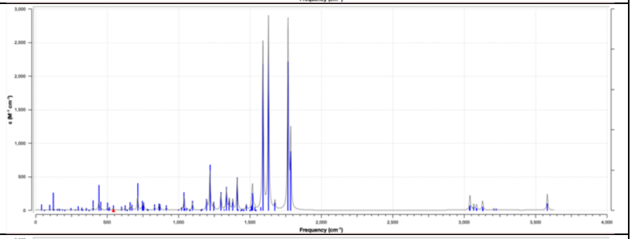
25	601.9323	5.6067	37.1593	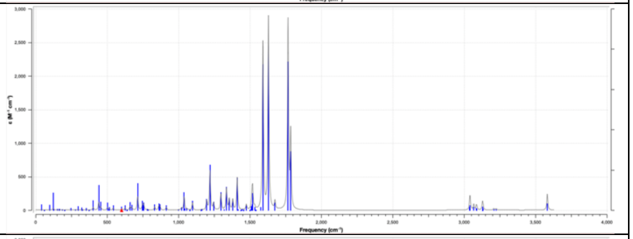
26	625.8865	8.9835	57.2608	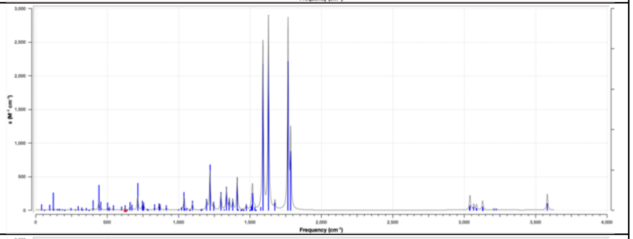
27	638.4217	0.0497	0.3106	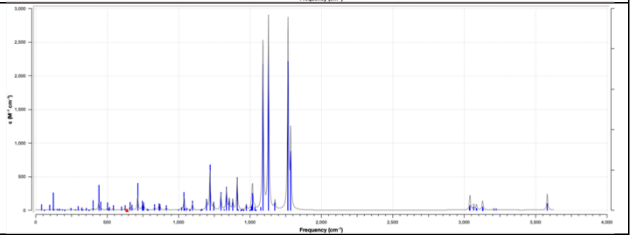

28	660.7533	15.2881	92.3042	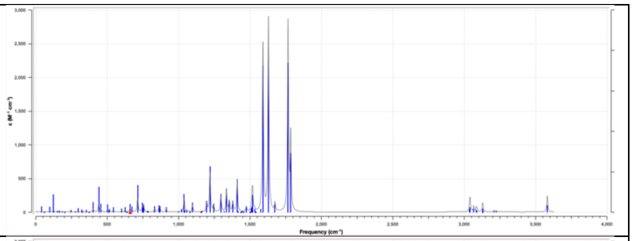
29	672.6403	10.8304	64.2346	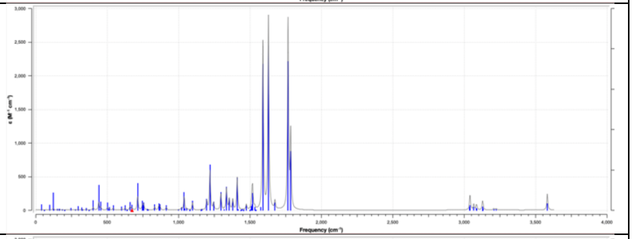
30	713.7040	59.2989	331.4636	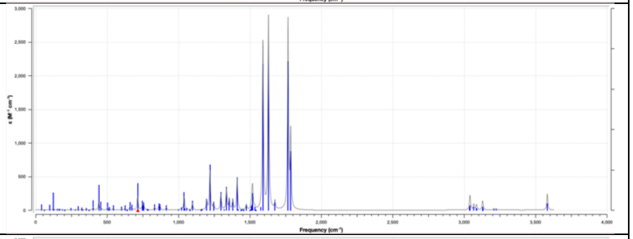
31	747.8530	21.0302	112.1849	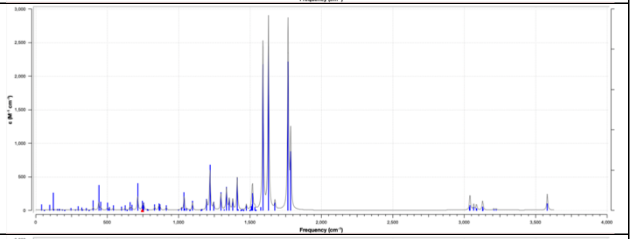
32	752.8403	11.1825	59.2575	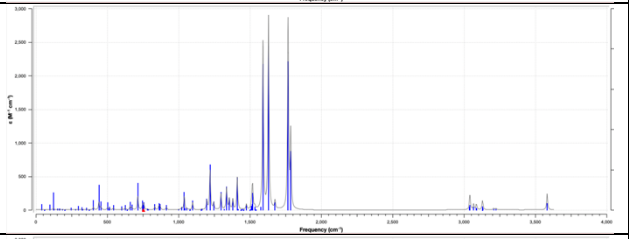
33	756.7400	16.6429	87.7384	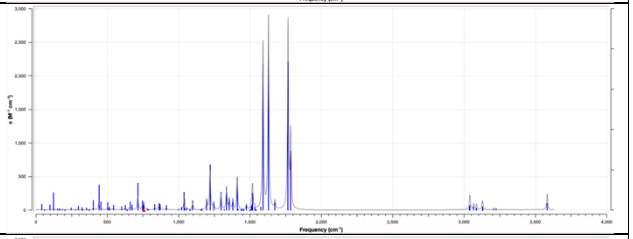
34	781.2296	1.9172	9.7903	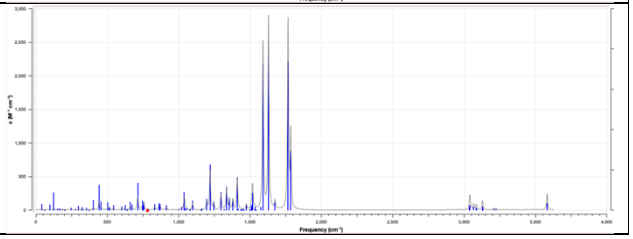

35	787.8614	0.7067	3.5784	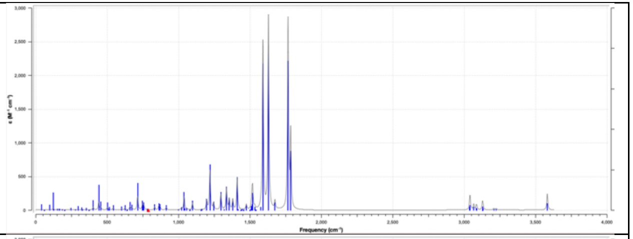
36	833.2059	13.5857	65.0485	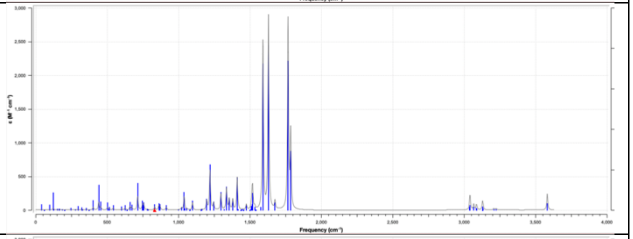
37	862.6750	17.4778	80.8252	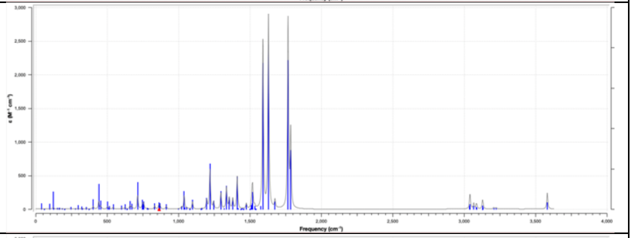
38	870.7014	15.1530	69.4283	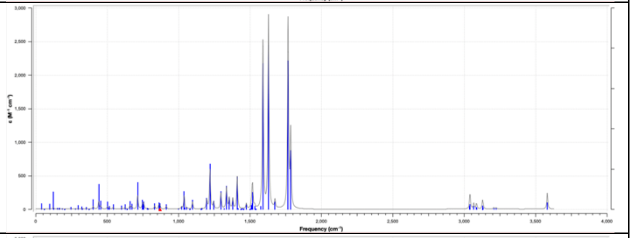
39	914.5787	12.3664	53.9423	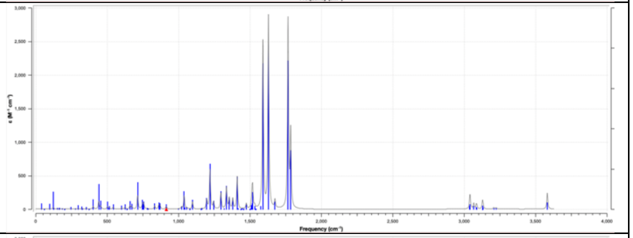
40	999.8854	1.4025	5.5958	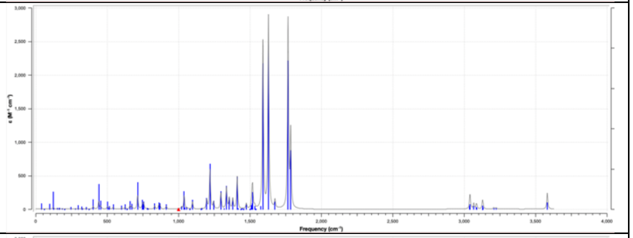
41	1021.7343	8.6832	33.9039	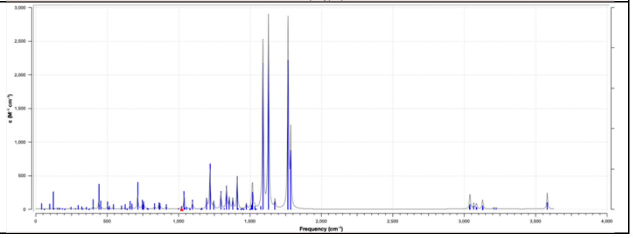

42	1039.5273	56.9113	218.4087	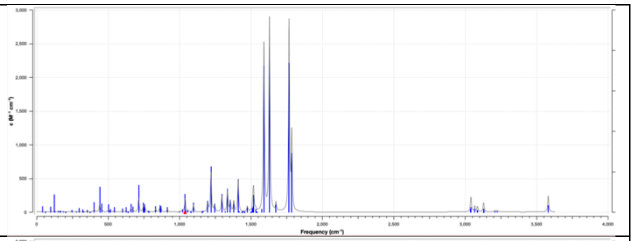
43	1045.1574	4.0687	15.5304	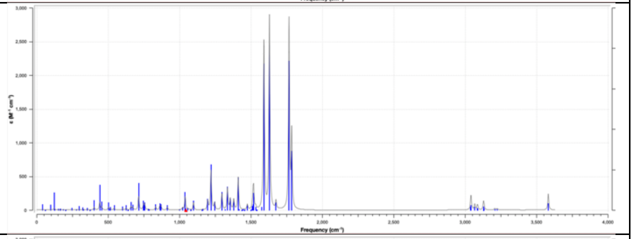
44	1058.4406	5.2993	19.9737	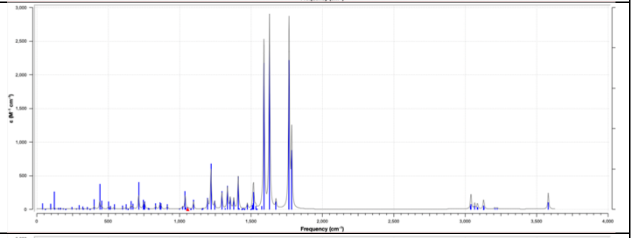
45	1079.1630	0.0259	0.0957	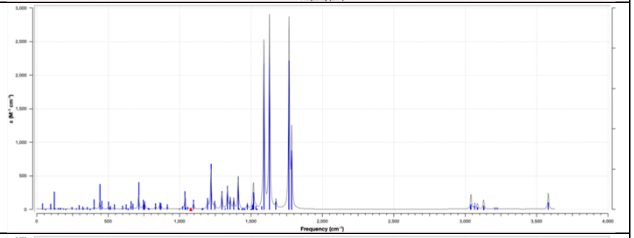
46	1097.9303	31.1056	113.0241	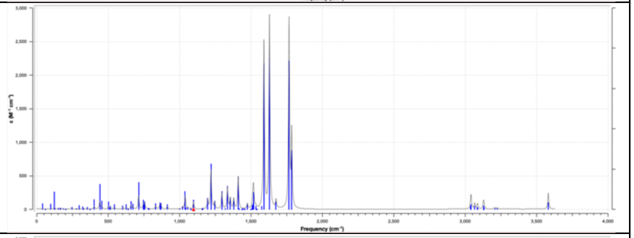
47	1157.0801	0.4278	1.4750	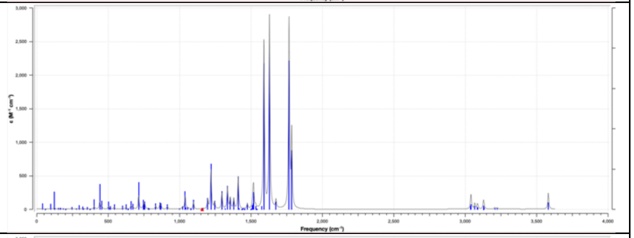
48	1165.3036	2.0714	7.0914	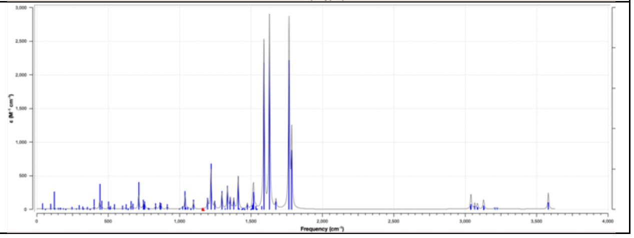

49	1197.4625	40.8618	136.1328	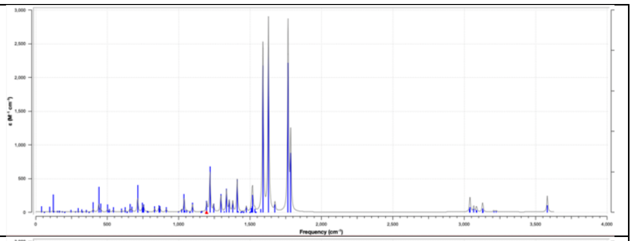
50	1220.3590	170.8631	558.5576	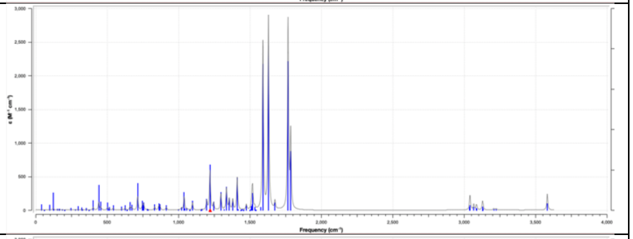
51	1245.7587	31.1685	99.8135	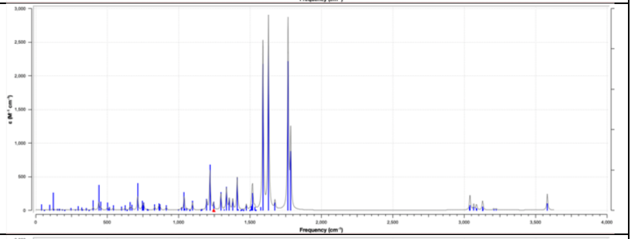
52	1296.6995	70.2287	216.0641	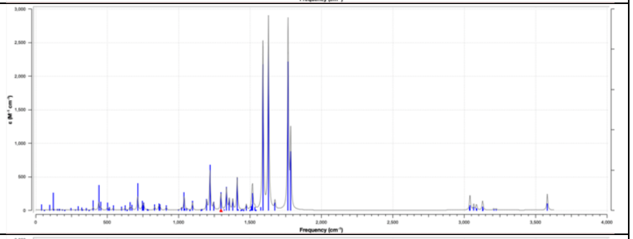
53	1319.4541	1.0618	3.2104	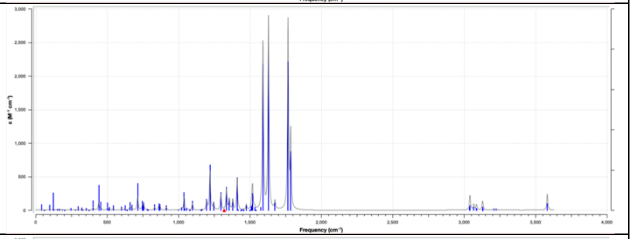
54	1335.7209	95.5755	285.4553	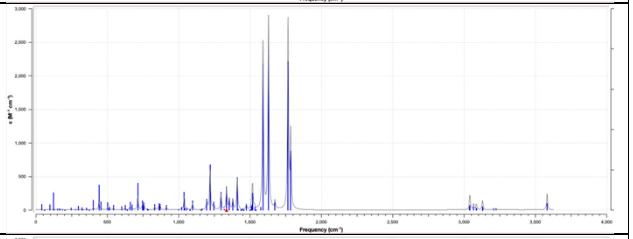
55	1355.3653	48.3428	142.2927	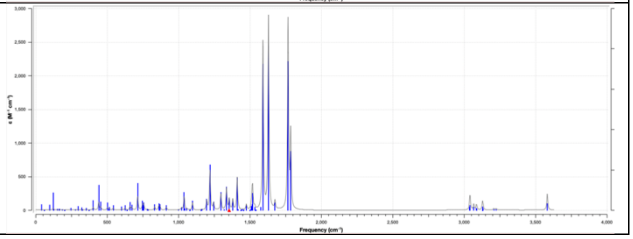

56	1379.8646	44.5661	128.8473	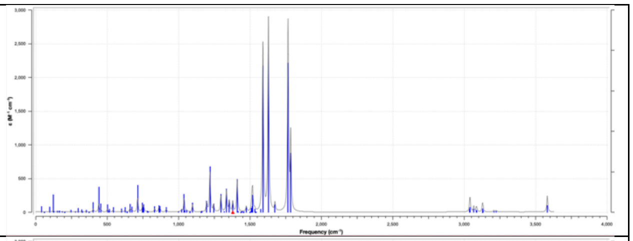
57	1410.9146	140.0535	396.0049	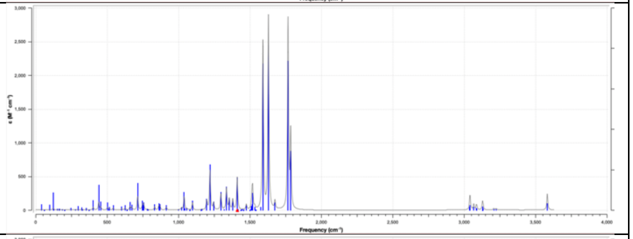
58	1416.6341	2.6848	7.5607	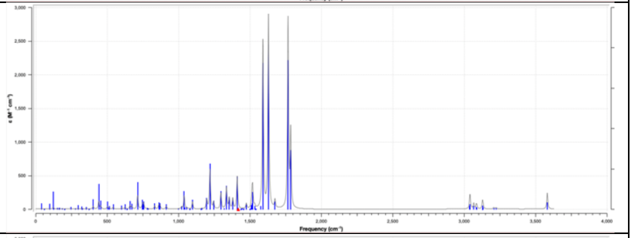
59	1435.1676	0.1686	0.4687	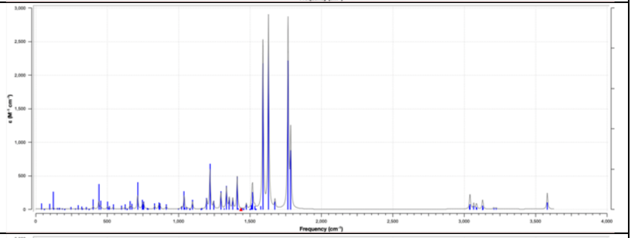
60	1447.1111	1.3655	3.7644	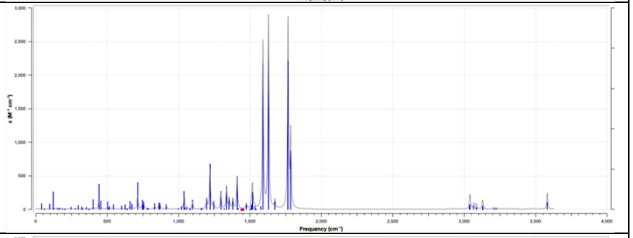
61	1455.9467	0.1001	0.2743	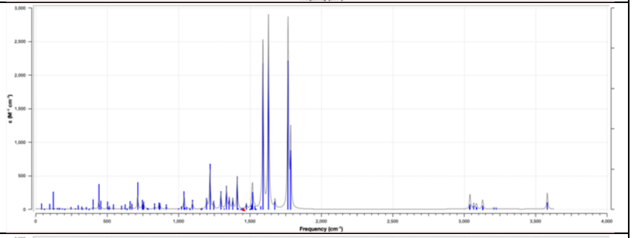
62	1474.6973	22.1483	59.9163	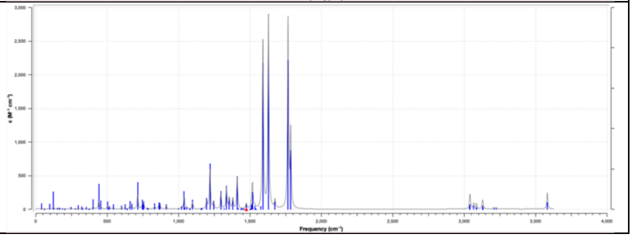

63	1498.8524	0.2041	0.5432	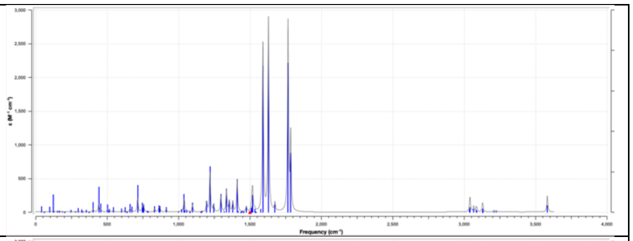
64	1510.2673	16.7702	44.2988	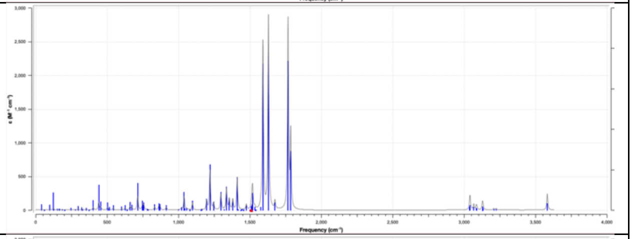
65	1514.9599	20.9064	55.0536	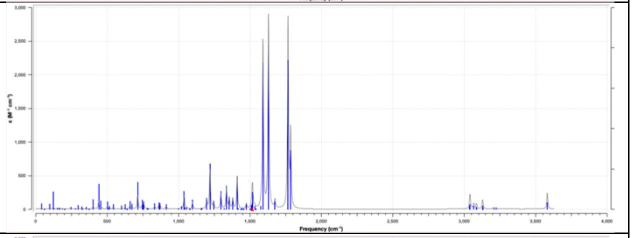
66	1515.8885	26.2385	69.0524	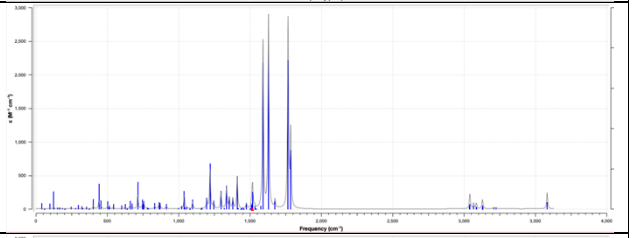
67	1518.7429	79.2066	208.0580	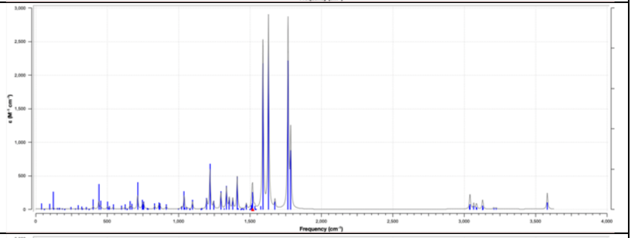
68	1533.9345	12.4952	32.4970	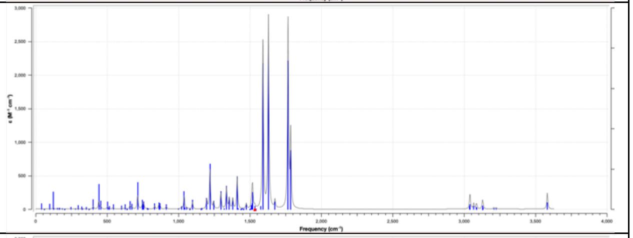
69	1539.9672	0.0018	0.0047	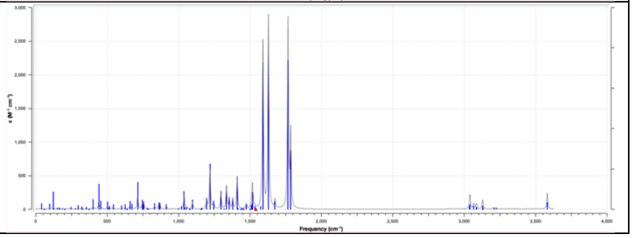

70	1577.3121	14.0277	35.4794	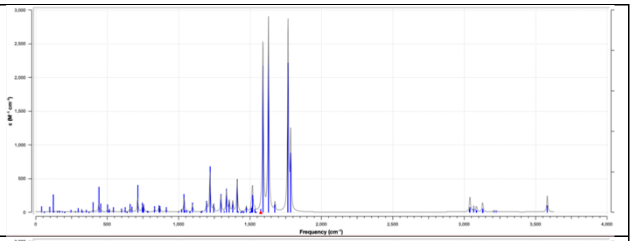
71	1590.9552	720.9223	1807.7477	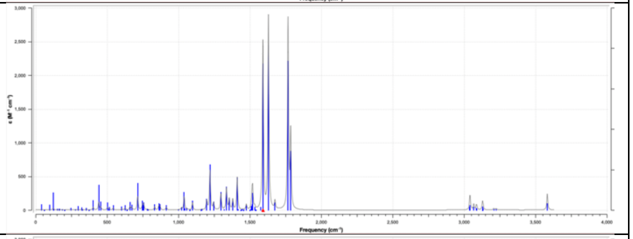
72	1629.5647	830.3150	2032.7248	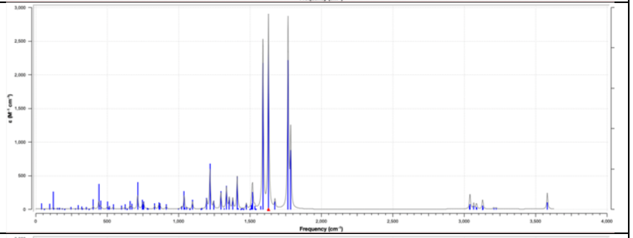
73	1675.4201	37.1206	88.3891	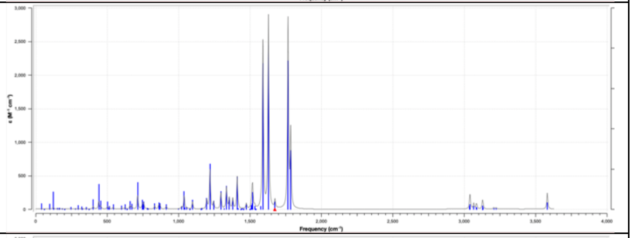
74	1767.0230	815.3195	1840.7420	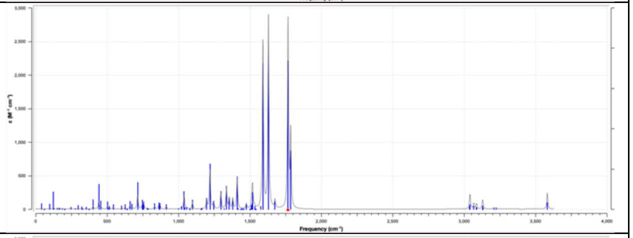
75	1785.3518	325.2143	726.6966	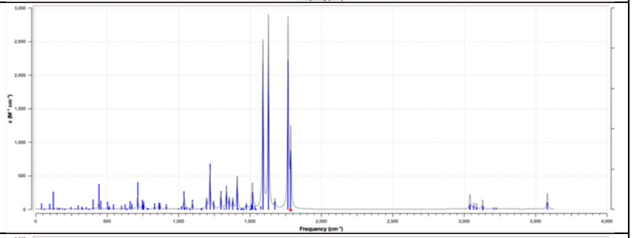
76	3039.1138	23.7180	31.1342	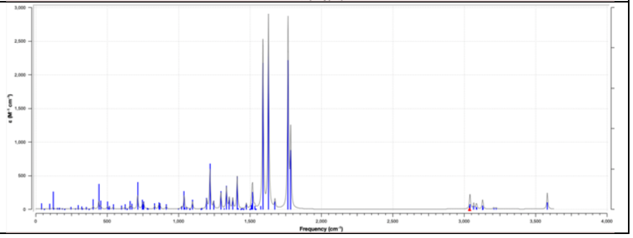

77	3041.9395	44.2560	58.0402	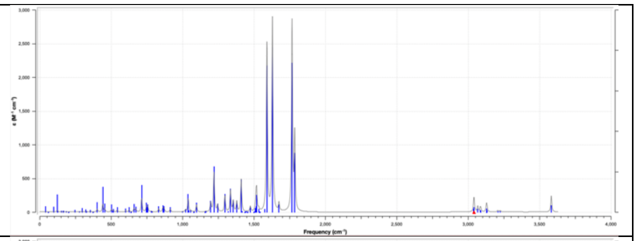
78	3067.5568	25.1797	32.7465	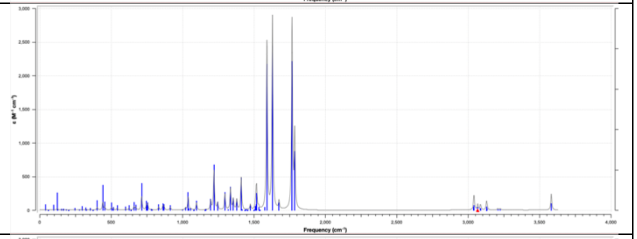
79	3085.8509	17.2298	22.2747	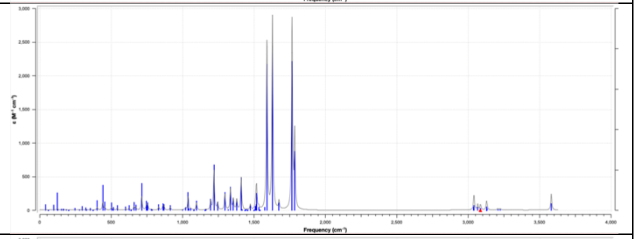
80	3089.5078	8.7493	11.2977	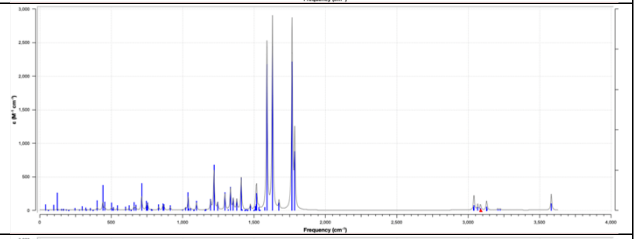
81	3128.6053	30.1985	38.5072	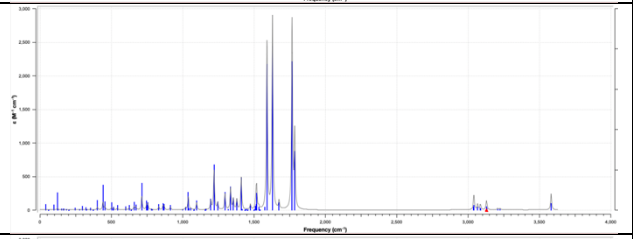
82	3129.8895	1.4745	1.8794	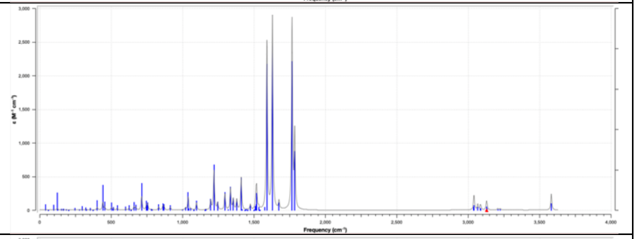
83	3132.1025	12.5878	16.0332	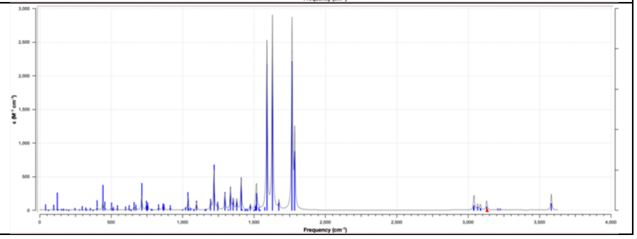

84	3207.6210	3.4281	4.2636	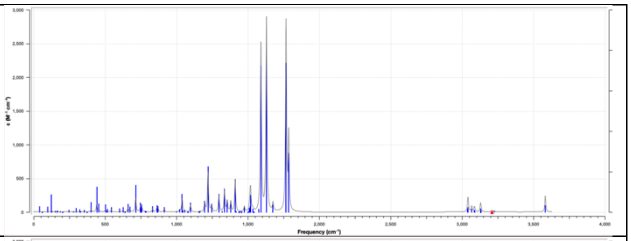
85	3207.9529	3.5001	4.3527	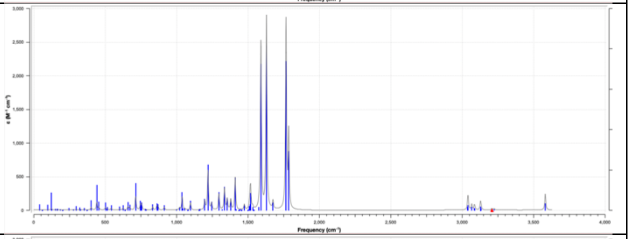
86	3224.7044	6.1958	7.6650	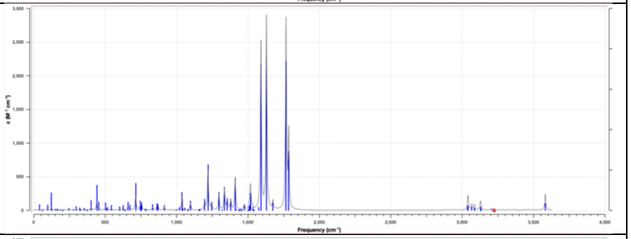
87	3582.6111	68.6621	76.4583	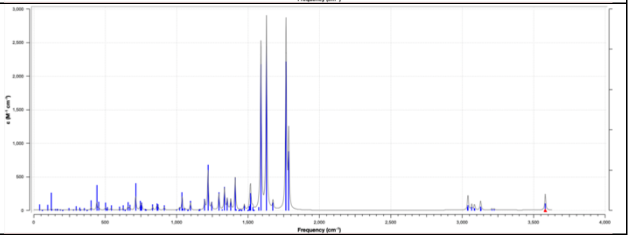

Table S2 *Statistical analysis of ΔRMSD/$E_{average}$ along 87 vibrational modes.*

**Mode**	**Δ RMSD/$E_{average}$**	**| Δ RMSD/$E_{average}$ |**	**S_1 more anharmonic | Δ RMSD/$E_{average}$ | > 0.00444**
1	0.00124099	0.00124099	NO
2	-0.00007956	0.00007956	NO
3	0.00122685	0.00122685	NO
4	-0.00041485	0.00041485	NO
5	0.00037531	0.00037531	NO
6	0.00027646	0.00027646	NO
7	0.01137812	0.01137812	YES
8	0.00025177	0.00025177	NO
9	0.00049045	0.00049045	NO
10	0.00061616	0.00061616	NO
11	0.00023860	0.00023860	NO
12	0.00432950	0.00432950	NO
13	0.00478096	0.00478096	YES
14	0.00341357	0.00341357	NO
15	-0.00022280	0.00022280	NO
16	0.00486795	0.00486795	YES
17	0.00073146	0.00073146	NO
18	0.00106613	0.00106613	NO
19	0.00207395	0.00207395	NO
20	0.00020079	0.00020079	NO
21	0.00227851	0.00227851	NO
22	0.00017735	0.00017735	NO
23	0.00003101	0.00003101	NO
24	0.00260871	0.00260871	NO
25	0.00109432	0.00109432	NO
26	-0.00007077	0.00007077	NO
27	-0.00036971	0.00036971	NO
28	0.00046981	0.00046981	NO
29	0.00151321	0.00151321	NO
30	-0.00054300	0.00054300	NO
31	0.01015283	0.01015283	YES
32	0.00150080	0.00150080	NO
33	0.01399649	0.01399649	YES
34	0.00575299	0.00575299	YES
35	0.00225981	0.00225981	NO
36	0.00184265	0.00184265	NO

37	0.00016691	0.00016691	NO
38	0.00071685	0.00071685	NO
39	0.00010172	0.00010172	NO
40	-0.00012641	0.00012641	NO
41	-0.00023347	0.00023347	NO
42	0.00051296	0.00051296	NO
43	0.00056334	0.00056334	NO
44	0.00011009	0.00011009	NO
45	0.00029651	0.00029651	NO
46	0.00013475	0.00013475	NO
47	-0.00007261	0.00007261	NO
48	-0.00061148	0.00061148	NO
49	0.00015876	0.00015876	NO
50	-0.00124629	0.00124629	NO
51	0.00241885	0.00241885	NO
52	0.00028515	0.00028515	NO
53	-0.00046947	0.00046947	NO
54	0.00066873	0.00066873	NO
55	0.00221968	0.00221968	NO
56	-0.00006462	0.00006462	NO
57	0.00046539	0.00046539	NO
58	0.00018982	0.00018982	NO
59	0.00044285	0.00044285	NO
60	0.00038209	0.00038209	NO
61	0.00009595	0.00009595	NO
62	0.00016871	0.00016871	NO
63	0.00005993	0.00005993	NO
64	0.00009922	0.00009922	NO
65	-0.00004468	0.00004468	NO
66	0.00009100	0.00009100	NO
67	0.00008331	0.00008331	NO
68	0.00030788	0.00030788	NO
69	0.00053390	0.00053390	NO
70	0.00605384	0.00605384	YES
71	0.00058720	0.00058720	NO
72	0.00373948	0.00373948	NO
73	-0.00341582	0.00341582	NO
74	0.01636748	0.01636748	YES
75	0.00219766	0.00219766	NO

76	0.00066975	0.00066975	NO
77	0.00089506	0.00089506	NO
78	0.00030332	0.00030332	NO
79	-0.00017568	0.00017568	NO
80	-0.00029408	0.00029408	NO
81	0.00027388	0.00027388	NO
82	-0.00022300	0.00022300	NO
83	0.00085637	0.00085637	NO
84	-0.00027067	0.00027067	NO
85	0.00018186	0.00018186	NO
86	0.00068452	0.00068452	NO
87	-0.00009887	0.00009887	NO
**Average**		0.00154	
**Standard deviation**		0.00290	
**Average + Standard deviation**		0.00444	

Table S3 *Statistical analysis at ground state (S_0) of RMSD/$E_{average}$ along 87 vibrational modes.*

**Mode**	**RMSD/$E_{average}$ (S_0)**	**S_0 is anharmonic (RMSD/$E_{average}$ (S_0) > 0.029659)**
1	0.003833048	NO
2	0.001674407	NO
3	0.001038101	NO
4	0.002092279	NO
5	0.001355052	NO
6	0.001463351	NO
7	0.000487733	NO
8	0.001844311	NO
9	0.001779226	NO
10	0.000385118	NO
11	0.001413321	NO
12	0.000835946	NO
13	0.001116258	NO
14	0.001698077	NO
15	0.002875967	NO
16	0.000204451	NO
17	0.002121431	NO
18	0.003139728	NO
19	0.002264373	NO
20	0.001663805	NO
21	0.000403977	NO
22	0.002710029	NO
23	0.003362813	NO
24	0.000229279	NO
25	0.000911943	NO
26	0.01019242	NO
27	0.002362099	NO
28	0.000356742	NO
29	0.001723141	NO
30	0.019512991	NO
31	0.000585977	NO
32	0.00403239	NO
33	0.019291706	NO
34	0.038569248	YES
35	0.000530687	NO
36	0.00285254	NO

37	0.079529361	YES
38	0.00032676	NO
39	0.076435178	YES
40	0.003954946	NO
41	0.00333315	NO
42	0.001306319	NO
43	0.010584836	NO
44	0.008498583	NO
45	0.010873246	NO
46	0.016269775	NO
47	0.023209221	NO
48	0.003808604	NO
49	0.001190515	NO
50	0.008663271	NO
51	0.002963912	NO
52	0.006142191	NO
53	0.005221133	NO
54	0.006707314	NO
55	0.004953737	NO
56	0.008366753	NO
57	0.005481464	NO
58	0.020141766	NO
59	0.01258463	NO
60	0.031574239	YES
61	0.008477479	NO
62	0.010214057	NO
63	0.031681896	YES
64	0.004124615	NO
65	0.010338429	NO
66	0.027259861	NO
67	0.013086633	NO
68	0.011298493	NO
69	0.003964667	NO
70	0.004585524	NO
71	0.003499385	NO
72	0.013389229	NO
73	0.015941617	NO
74	0.052686257	YES
75	0.010882048	NO

76	0.005903173	NO
77	0.005666473	NO
78	0.006730223	NO
79	0.023236973	NO
80	0.022206594	NO
81	0.014137767	NO
82	0.018932364	NO
83	0.001735257	NO
84	0.07800239	YES
85	0.036859499	YES
86	0.040955431	YES
87	0.07188469	YES
**Average**	0.011962	
**Standard deviation**	0.017696963	
**Average + Standard deviation**	0.029659	

Table S4 *Statistical analysis of relaxation energy (λ) along 87 vibrational modes.*

**Mode**	**λ**	**FC active (λ > 0.009087)**
1	0.0007293	NO
2	0.0050205	NO
3	0.0044382	NO
4	0.003962	NO
5	0	NO
6	0	NO
7	0.0007402	NO
8	0.0000545	NO
9	0	NO
10	0.0016354	NO
11	0	NO
12	0.0015919	NO
13	0.000264	NO
14	0.0005552	NO
15	0.0003837	NO
16	0	NO
17	0.0034423	NO
18	0.0008953	NO
19	0.0000599	NO
20	0.0012436	NO
21	0	NO
22	0	NO
23	0.0008463	NO
24	0.00003	NO
25	0.0102016	YES
26	0.0082043	NO
27	0.0057253	NO
28	0.0061063	NO
29	0.0005334	NO
30	0.0086288	NO
31	0.0034668	NO
32	0.0004137	NO
33	0.0225556	YES
34	0.0115594	YES
35	0.006909	NO
36	0.0004082	NO

37	0	NO
38	0.0002205	NO
39	0	NO
40	0.0021715	NO
41	0.0006259	NO
42	0	NO
43	0	NO
44	0	NO
45	0	NO
46	0.0005688	NO
47	0	NO
48	0	NO
49	0.0054396	NO
50	0.0094614	YES
51	0.0135241	YES
52	0.0008218	NO
53	0.0007103	NO
54	0.0000245	NO
55	0.0059131	NO
56	0	NO
57	0	NO
58	0.0007157	NO
59	0	NO
60	0	NO
61	0.0005225	NO
62	0	NO
63	0	NO
64	0	NO
65	0	NO
66	0	NO
67	0.0010776	NO
68	0	NO
69	0	NO
70	0.013584	YES
71	0.0005443	NO
72	0.0481778	YES
73	0.0137146	YES
74	0.0010314	NO
75	0.0035756	NO

76	0	NO
77	0	NO
78	0	NO
79	0.0001769	NO
80	0	NO
81	0	NO
82	0	NO
83	0	NO
84	0	NO
85	0	NO
86	0	NO
87	0	NO
**Average**	0.00268	
**Standard deviation**	0.00641	
**Average + Standard deviation**	0.00908	
